# SPIOMET4HEALTH—efficacy, tolerability and safety of lifestyle intervention plus a fixed dose combination of spironolactone, pioglitazone and metformin (SPIOMET) for adolescent girls and young women with polycystic ovary syndrome: study protocol for a multicentre, randomised, double-blind, placebo-controlled, four-arm, parallel-group, phase II clinical trial

**DOI:** 10.1186/s13063-023-07593-6

**Published:** 2023-09-15

**Authors:** Cristina Garcia-Beltran, Rita Malpique, Marianne S. Andersen, Firdevs Bas, Judit Bassols, Feyza Darendeliler, Marta Díaz, Barbara Dieris, Flaminia Fanelli, Elke Fröhlich-Reiterer, Alessandra Gambineri, Dorte Glintborg, Abel López-Bermejo, Christopher Mann, Silvia Marin, Barbara Obermayer-Pietsch, Rønnaug Ødegård, Pernille Ravn, Thomas Reinehr, Matteo Renzulli, Cristina Salvador, Viola Singer, Eszter Vanky, Juan Vicente Torres, Melek Yildiz, Francis de Zegher, Lourdes Ibáñez

**Affiliations:** 1 Paediatric Endocrinology, Paediatric Research Institute Sant Joan de Déu, University of Barcelona, 08950 Esplugues, Barcelona, Spain; 2 Centro de Investigación Biomédica en Red de Diabetes y Enfermedades Metabólicas Asociadas (CIBERDEM), ISCIII, Madrid, 28029 Spain; 3 Department of Gynaecology and Obstetrics and Department of Endocrinology, University of Southern Denmark, Odense University Hospital, Odense, Denmark; 4 Pediatric Endocrinology Unit, Istanbul University, Istanbul, Turkey; 5 Maternal-Fetal Metabolic Research Group, Girona Institute for Biomedical Research (IDIBGI), Girona, Spain; 6 Department of Paediatric Endocrinology, Diabetes and Nutrition Medicine, Vestische Hospital for Children and Adolescents Datteln, University of Witten-Herdecke, Datteln, Germany; 7 Department of Medical and Surgical Science-DIMEC, Division of Endocrinology and Diabetes Prevention and Care, University of Bologna - S. Orsola-Hospital, Bologna, Italy; 8 Division of General Paediatrics, Department of Paediatrics and Adolescent Medicine, Medical University of Graz, Graz, Austria; 9 Paediatric Endocrinology Research Group, Girona Institute for Biomedical Research (IDIBGI), Paediatrics, Dr. Josep Trueta Hospital, Department of Medical Sciences, University of Girona, Girona, Spain; 10 Asphalion, Barcelona, Spain; 11 Division of Endocrinology and Diabetology, Department of Internal Medicine, Medical University of Graz, Graz, Austria; 12 Department of Clinical and Molecular Medicine, Norwegian University of Science and Technology, Trondheim, Norway; 13 Centre for Obesity Research, St. Olavs Hospital, Trondheim University Hospital, Torgarden, Trondheim, Norway; 14 Department of Radiology, IRCCS Azienda Ospedaliero-Universitaria Di Bologna, Bologna, Italy; 15 Department of Obstetrics and Gynaecology, St. Olavs Hospital, Trondheim University Hospital, 7006 Trondheim, Norway; 16 Optimapharm, Palma de Mallorca, Palma, Spain; 17 Leuven Research & Development, University of Leuven, 3000 Louvain, Belgium

**Keywords:** Androgen excess, Polycystic ovary syndrome, PCOS, Ectopic fat, Liver fat, Central obesity, Anovulation, Pioglitazone, Spironolactone, Metformin

## Abstract

**Background:**

Polycystic ovary syndrome (PCOS) is the most prevalent, chronic endocrine-metabolic disorder of adolescents and young women (AYAs), affecting 5–10% of AYAs worldwide. There is no approved pharmacological therapy for PCOS. Standard off-label treatment with oral contraceptives (OCs) reverts neither the underlying pathophysiology nor the associated co-morbidities. Pilot studies have generated new insights into the pathogenesis of PCOS, leading to the development of a new treatment consisting of a fixed, low-dose combination of two so-called insulin sensitisers [pioglitazone (PIO), metformin (MET)] and one mixed anti-androgen and anti-mineralocorticoid also acting as an activator of brown adipose tissue [spironolactone (SPI)], within a single tablet (SPIOMET). The present trial will evaluate the efficacy, tolerability and safety of SPIOMET, on top of lifestyle measures, for the treatment of PCOS in AYAs.

**Methods:**

In this multicentre, randomised, double-blind, placebo-controlled, four-arm, parallel-group, phase II clinical trial, AYAs with PCOS will be recruited from 7 clinical centres across Europe. Intention is to randomise a total of 364 eligible patients into four arms (1:1:1:1): Placebo, PIO, SPI + PIO (SPIO) and SPI + PIO + MET (SPIOMET). Active treatment over 12 months will consist of lifestyle guidance plus the ingestion of one tablet daily (at dinner time); post-treatment follow-up will span 6 months. Primary endpoint is on- and post-treatment ovulation rate. Secondary endpoints are clinical features (hirsutism, menstrual regularity); endocrine-metabolic variables (androgens, lipids, insulin, inflammatory markers); epigenetic markers; imaging data (carotid intima-media thickness, body composition, abdominal fat partitioning, hepatic fat); safety profile; adherence, tolerability and acceptability of the medication; and quality of life in the study participants. Superiority (in this order) of SPIOMET, SPIO and PIO will be tested over placebo, and if present, subsequently the superiority of SPIOMET versus PIO, and if still present, finally versus SPIO.

**Discussion:**

The present study will be the first to evaluate—in a randomised, double-blind, placebo-controlled way—the efficacy, tolerability and safety of SPIOMET treatment for early PCOS, on top of a lifestyle intervention.

**Trial registration:**

EudraCT 2021–003177-58. Registered on 22 December 2021.

https://www.clinicaltrialsregister.eu/ctr-search/search?query=%092021-003177-58.

**Supplementary Information:**

The online version contains supplementary material available at 10.1186/s13063-023-07593-6.

## Administrative information

**Table:** 

Title {1}	SPIOMET4HEALTH – Efficacy, Tolerability and Safety of a Fixed Dose Combination of Spironolactone, Pioglitazone & Metformin (SPIOMET) plus Lifestyle Intervention for Adolescent Girls and Young Women with Polycystic Ovary Syndrome: Study Protocol for a Multicentre, Randomised, Double-Blind, Placebo-Controlled, Four-Arm Parallel Groups Clinical Trial
Trial registration {2a and 2b}	EudraCT: 2021–003177-58
Protocol version {3}	Version 4.0; 15 November 2022
Funding {4}	The SPIOMET4HEALTH project received funding from the European Union’s Horizon 2020 research and innovation programme under grant agreement No 899671. The funding body had no role in the design of the study, in the collection, analysis and interpretation of data and in writing the manuscript
Author details {5a}	^1^Paediatric Endocrinology, Paediatric Research Institute Sant Joan de Déu, University of Barcelona, 08950 Esplugues, Barcelona, Spain ^2^Centro de Investigación Biomédica en Red de Diabetes y Enfermedades Metabólicas Asociadas (CIBERDEM), ISCIII, 28029 Madrid, Spain ^3^Department of Gynaecology and Obstetrics and Department of Endocrinology, University of Southern Denmark, Odense University Hospital, Odense, Denmark ^4^Pediatric Endocrinology Unit, Istanbul University, Istanbul, Turkey ^5^Maternal-Fetal Metabolic Research Group, Girona Institute for Biomedical Research (IDIBGI), Girona, Spain ^6^Department of Paediatric Endocrinology, Diabetes and Nutrition Medicine, Vestische Hospital for Children and Adolescents Datteln, University of Witten-Herdecke, Datteln, Germany ^7^Department of Medical and Surgical Science-DIMEC, Division of Endocrinology and Diabetes Prevention and Care, University of Bologna—S. Orsola-Hospital, Bologna, Italy ^8^Division of General Paediatrics, Department of Paediatrics and Adolescent Medicine, Medical University of Graz, Graz, Austria ^9^Paediatric Endocrinology Research Group, Girona Institute for Biomedical Research (IDIBGI); Paediatrics, Dr. Josep Trueta Hospital; and Department of Medical Sciences, University of Girona, Girona, Spain ^10^Asphalion, Barcelona, Spain ^11^Division of Endocrinology and Diabetology, Department of Internal Medicine, Medical University of Graz, Graz, Austria ^12^Department of Clinical and Molecular Medicine, Norwegian University of Science and Technology, Trondheim, Norway ^13^Centre for Obesity Research, St. Olavs Hospital, Trondheim University Hospital, Torgarden, Trondheim, Norway ^14^Department of Radiology, IRCCS Azienda Ospedaliero-Universitaria di Bologna, Italy ^15^Department of Obstetrics and Gynaecology, St. Olavs Hospital, Trondheim University Hospital, 7006 Trondheim, Norway ^16^Optimapharm, Palma de Mallorca, Spain ^17^Leuven Research & Development, University of Leuven, 3000 Leuven, Belgium
Name and contact information for the trial sponsor {5b}	Fundació de Recerca Sant Joan de Déu Carrer de Santa Rosa 39–57 08950 Esplugues de Llobregat, Barcelona Spain
Role of sponsor {5c}	The funding instance has no role in the study design, data collection, data analysis and interpretation, writing the report or decision to submit the report for publication. The Sponsor will act as recruiting partner and as the coordinator of the clinical trial

## Introduction

### Background and rationale {6a}

Polycystic ovary syndrome (PCOS) is the most common endocrine disorder in women of reproductive age. The overall prevalence of PCOS is around 5–10% [1] but could be as high as 26% in some populations [2]. The most common features of PCOS include clinical and/or biochemical hyperandrogenism, menstrual irregularities and oligo-anovulation [3, 4]. Women with PCOS also have an elevated prevalence of obesity, and a significant proportion also display insulin resistance, even in the absence of obesity [5].

PCOS is the most frequent cause of anovulatory subfertility and is associated with lifelong co-morbidities including type 2 diabetes (T2D), premature vascular ageing, premenopausal cancer, non-alcoholic fatty liver disease, gestational complications and anxiety/depression, with a negative impact on the health-related quality of life (HRQoL) of these subjects and their offspring [6–11]. Overweight and obesity amplify the metabolic-reproductive abnormalities and the risks for T2D and cardiovascular complications [5].

Over a quarter of a century, Ibáñez and de Zegher et al. have generated new insights into the pathophysiology of PCOS, including on the developmental sequence of events leading to the disorder [12, 13]. Those evolving concepts have prompted a shift in the therapeutic approach towards interventions guided by the pathophysiology [14, 15]. Nowadays, PCOS is thought to be, in essence, the result of a mismatch between prenatal adipogenesis and postnatal lipogenesis, which can be inferred from a mismatch between prenatal weight gain and postnatal weight gain, and results in a chronic need to store more fat than is safely feasible in the subcutaneous fraction of adipose tissue [12, 13, 16, 17]. The excess fat tends to be stored in ectopic depots, especially in the liver and viscera (hepato-visceral or central fat) [12, 13]. The extent of such storage may be influenced by genetic and epigenetic factors and may be raised by a reduced activity of brown adipose tissue (BAT) that contributes to a more positive energy balance [18–20].

So far, no treatment for PCOS in adolescent girls or young women (AYAs) has been approved by the European Medicines Agency (EMA) or the US Food and Drug Administration (FDA). Oral contraceptives (OCs) are frequently prescribed off-label as first-line treatment in adolescent girls, including in those who do not need contraception [4, 21, 22]. OCs alleviate the clinical symptoms by inducing a combination of anovulatory infertility, regular pseudo-menses and pharmacological elevations of sex hormone-binding globulin (SHBG) [23, 24]. However, they do not revert the underlying pathophysiology so that, upon treatment discontinuation, the entire PCOS phenotype reappears, with risk for subfertility and for potentially lifelong comorbidities that are relevant for public health [25, 26]. Indeed, OC treatment in AYAs with PCOS fails to normalise insulin resistance, the circulating levels of ultra-sensitive C-reactive protein (us-CRP, a marker of low-grade inflammation), the carotid intima-media thickness (cIMT, a marker of subclinical atherosclerosis) or the ectopic fat [13, 27–30]. Likewise, in the general population, the use of contemporary OCs has been associated with increased risk for breast cancer, glioma and venous thromboembolism, which may vary with oestrogen dose and progestogen type [31–33]. In women with PCOS, the use of OCs with purportedly low thrombogenic potential has nevertheless been linked to risk for inflammatory and coagulation disorders [34]. Given the hypothesised key role of ectopic (hepato-visceral) fat or central obesity in the pathogenesis of PCOS, the focus of the treatment should be to reduce ectopic fat. In turn, such reduction should have normalising effects on the entire PCOS phenotype.

Pilot studies have been conducted by Ibáñez’ group in AYAs with PCOS and without obesity, leading to the identification of a treatment that consists of a fixed low-dose combination of two insulin sensitisers [pioglitazone (PIO) and metformin (MET), acting through different pathways] and one mixed anti-androgen and anti-mineralocorticoid [spironolactone (SPI)], also acting as BAT activator and thus as driver of energy expenditure. In those studies, the triple low-dose combination of SPI, PIO and MET—so-called SPIOMET—proved to be superior to an OC in normalising the PCOS phenotype, including post-treatment ovulation rate and hepato-visceral fat [14, 15]. The chronological build-up towards SPIOMET started with MET, continued with the addition of a pure anti-androgen (flutamide), and then with the further addition of PIO; in a final step, flutamide was replaced by SPI, in order to take advantage of its mixed anti-androgen, anti-mineralocorticoid and BAT-activating properties [14, 15, 35–45].

So far, the effects of SPIOMET administered in three separate tablets vs an OC have been investigated in two randomised, controlled, open-label, pilot studies [ISRCTN29234515 and ISRCTN11062950; [14, 15]] performed between 2012 and 2019, that were based in a single centre in Barcelona and that were conducted in adolescents with PCOS and without obesity who did not need contraception [total *N* = 62; age 16 yr; body mass index (BMI) 24 kg/m^2^; treatment for 1 year; ovulation assessment during the post-treatment year]. The pooled results from those pilot studies disclosed that SPIOMET has more normalising effects than OCs, particularly in decreasing hepato-visceral fat and insulin resistance, and results in threefold more ovulations after discontinuation of the intervention [14, 15]. In the present clinical trial, SPIOMET will be administered in a single tablet of immediate release containing the three active substances: SPI, 50 mg; PIO, 7.5 mg; MET, 850 mg. The bioequivalence of SPIOMET in a single tablet vs the co-intake of the three separate compounds has been shown in a phase I study [46]. The treatment simplification into the intake of a single daily tablet is expected to raise compliance particularly over the longer term. Studies of other medical conditions have shown that adherence rates are higher with fixed-dose combinations (FDC) than with multiple-tablet therapies [47, 48].

The active substances in the new fixed, low-dose SPIOMET combination (SPI, PIO and MET) are medications that have long been approved for other indications. All three are nowadays available as generic drugs that have been in clinical use for > 25 years, and have been given to millions of humans.

SPI is a steroidal aldosterone antagonist marketed as diuretic but can serve as an anti-androgen if given in higher doses (up to 200 mg/d). SPI has recently been identified as a potent activator of BAT which, in turn, may raise energy expenditure and ultimately result in a lower amount of ectopic fat [45]. SPI was first approved in 1960 and has thus been used in human medicine for > 60 years, primarily for heart failure but also for other cardiovascular conditions. In Europe, SPI has been licensed for oedematous conditions in children at doses of ~ 3 mg/kg. So far, SPI treatment in younger individuals has not been associated with safety concerns [49]. In Europe and in the USA, SPI has for decades been the safe anti-androgen of choice in the treatment of hirsutism with or without hyperandrogenism [50]. SPI treatment in higher dose (100 mg/d or more) is frequently accompanied by menstrual irregularity, and more rarely by abdominal pain, polyuria or dryness of the mouth [51]. There are essentially no safety concerns when spironolactone is dosed at only 50 mg/d (equal or less than 1 mg/kg/d), as will be the case in the present trial. Epidemiologic and cohort-based studies show no evidence of an increased cancer risk associated with SPI use [52].

PIO is a thiazolidinedione (TZD) acting as insulin sensitiser in adipose tissue, liver and muscle. It raises circulating high molecular weight adiponectin (HMW-adip) [43] and also insulin sensitivity via preferentially subcutaneous adipogenesis [53]. In 1999, PIO was approved in monotherapy and, in 2006, in a FDC with MET [54]. In women with PCOS, PIO improves insulin sensitivity, menstrual cyclicity and ovulation rates [55]. Peroxisome proliferator-activated receptor (PPAR)-gamma is generally viewed as the target of TZD class; however, at low dose (7.5 mg/d), PIO may act as an inhibitor of cyclin-dependent kinase 5 (CDK5)-mediated phosphorylation of PPAR-gamma rather than as a PPAR-gamma agonist [56]. The use of PIO has been questioned due to safety concerns, particularly those associated with a purported higher risk for bladder cancer in older men with diabetes. However, FDA performed a 10-year prospective study to establish the connection between pioglitazone and bladder cancer, concluding that it was non-existing [57]. In studies performed in adolescents with PCOS, low-dose PIO (7.5 mg), combined either with MET or with MET plus an anti-androgen, has shown an excellent safety profile [14, 15, 42]. In addition, PIO appears to be well tolerated by children since no side effects were identified in children with autism receiving high doses [58]. In 2008, PIO was flagged for long-term safety and was granted a class waiver by the EMA for paediatric studies for the treatment of type 2 diabetes despite the existing studies. Therefore, it is not licensed in the paediatric population for this indication. However, the Paediatric Committee (PDCO) of the EMA has left the door open for possible future developments of this active substance in other indications. A proof of that is the number of clinical trials using PIO in the paediatric population nowadays [search for “pioglitazone” (search term) and “age group: childbirth – 17 yr” in clinicaltrials.gov]. The safety and efficacy data of PIO in the paediatric population is limited and dosing recommendations are not available; however, the dose ranges investigated in adolescents are 15–45 mg. The proposed dose in the present study (7.5 mg/d) is lower and has been previously used by the present investigators [14, 15, 27, 42, 43]. Therefore, this dose appears to be acceptable in the absence of a standard dose. So far, a total of 31 girls aged 15.7 ± 0.2 years have been exposed to the SPIOMET combination (with three separate tablets) for 1 year [15] and there were no noteworthy adverse events.

MET has anti-diabetic properties by acting via multiple mechanisms. MET is known to raise AMP-activated protein kinase (AMPK) activity and the circulating concentrations of growth-and-differentiation factor 15 (GDF15), a peptide hormone that may reduce appetite by acting via a specific receptor in the brainstem [59–64]. MET was first approved in 1959, and since then several FDCs containing MET have been approved for the treatment of type 2 diabetes as first and/or second-line therapies [65]. MET is the drug most widely used for the treatment of T2D in adults and in children aged > 10.0 years; its use has significantly increased in non-diabetic younger children and adolescents [66–68]. Although MET has a limited effect on hirsutism, its benefits on other PCOS-associated features and co-morbidities have long been recognised in adult women; some of these benefits have also been reported in girls and younger women, including for ovulation induction [35, 69]; consequently, MET is commonly used off-label in women with PCOS (https://www.nhs.uk/conditions/polycystic-ovary-syndrome-pcos/treatment/). Extensive experience has been gathered over the past 60 years in relation to the clinical use and safety of MET [70]. In 2001, the EMA issued a favourable benefit/risk ratio for MET that outlines its safety for use in humans [65]. The main undesirable effects are gastrointestinal symptoms such as nausea, vomiting, diarrhoea, abdominal pain and loss of appetite (> 10%), which usually resolve shortly after initiation of the treatment [69]. Lactic acidosis has been only described in case of inappropriate use, i.e. in the presence of renal, cardiac and hepatic failure, or after intentional overdose [71]. A decrease of vitamin B12 serum levels has been observed in patients treated long-term with metformin but is rarely of clinical significance [72]. The proposed dose of MET (850 mg/d) is at the lower limit of the recommended range (850–2000 mg/d) [73].

Table 1 summarises the targeted efficacy and safety of the separate SPIOMET components and of their ensemble in AYAs with PCOS. The present study aims to strengthen the evidence that SPIOMET can confer additive benefits without eliciting safety concerns [14, 15, 43, 45, 59–64, 74]. The benefits of each compound on outcomes partly overlap and are essentially present with low doses, whereas the side effects differ and are essentially absent with low doses.

**Table 1:** Main targets and safety of SPIOMET

	**Main targets**	**Side effects to be avoided**	**Efficacy with low doses**	**Safety concerns with low doses**
SPI (50 mg/d)	Less androgen effect More BAT activity (CXCL14)	Hyperkalaemia	+	None
PIO (7.5 mg/d)	Less hepatic fat More HMW-adip	Weight gain	+	None
MET (850 mg/d)	Multiple mechanisms Less appetite (GDF15)	Abdominal discomfort	+	None
SPIOMET	Improved metabolic health, including less ectopic fat and more ovulations	Safety concerns	+ + +	None

*Abbreviations*: *BAT* Brown adipose tissue, *CXCL14* C-X-C motif chemokine ligand-14, *GDF15* Growth-and-differentiation factor 15, *HMW-adip* High molecular weight adiponectin, *MET* Metformin, *PIO* Pioglitazone, *SPI* Spironolactone, *SPIOMET* Spironolactone, pioglitazone and metformin

Table 2 provides justification for the dose of each SPIOMET component. Potential interactions of spironolactone and pioglitazone related to cytochrome P450 2C8 (CYP2C8)-mediated metabolism are of little concern, as there is no evidence for clinically relevant drug-drug interactions between these medications [75].

**Table 2:** Justification for the dose of every component of SPIOMET

	**No need for a higher dose in order to augment efficacy**	**No need for a lower dose in order to reduce side effects**
SPI (50 mg/d)	SPI is a BAT activator [45] Circulating concentrations of CXCL14, a marker of BAT activity, are low in adolescent girls with PCOS, and this low SPI dose is enough to raise circulating CXCL14 towards normal [74]	> 50 years of experience do not point to safety concerns with doses ≤ 1 mg/kg/d 50 mg/d is at the lower limit of the recommended range (50–200 mg/d) [14]
PIO (7.5 mg/d)	HMW-adip is an adipokine with insulin-sensitising and anti-inflammatory properties Circulating concentrations of HMW-adip are low in adolescent girls with PCOS, and this low pioglitazone dose is enough to raise circulating HMW-adip towards normal concentrations, and even above normal [43]	> 25 years of experience do not point to safety concerns with a dose of 7.5 mg/d In adolescent girls with PCOS, a dose of 7.5 mg/d corresponds to approximately 0.1 mg/kg/d, whereas children are known to tolerate doses of 0.25, 0.50 and 0.75 mg/kg/d [58]
MET (850 mg/d)	Circulating GDF15 has been unmasked as a key mediator of metformin effects [60] In adolescent girls with PCOS, a metformin dose of 850 mg/d is enough to raise circulating GDF15 nearly threefold above the levels in healthy controls [61]	850 mg/d is at the lower limit of the recommended range (850–2000 mg/d) [14] EMA & FDA authorised the use of metformin in doses up to 2000 mg/d in children with type 2 diabetes

*Abbreviations*: *BAT* Brown adipose tissue, *CXCL14* C-X-C motif chemokine ligand-14, *EMA* European Medicine Agency, *FDA* US Food and Drug Administration, *GDF15* Growth-and-differentiation factor 15, *HMW-adip* High molecular weight adiponectin, *MET* Metformin, *PCOS* Polycystic ovary syndrome, *PIO* Pioglitazone, *SPI* Spironolactone

In parallel with the pharmacological intervention, a specific Lifestyle Intervention Programme (LIP) has been developed for AYAs and will be implemented during the time window of medication intake. This intervention is mostly based on the well-established lifestyle intervention “Obeldicks” [76], which is effective to treat hyperandrogenaemia [77] and PCOS-related symptoms [78] in patients with obesity; it focuses on strengths and not on weaknesses and thus, on motivating participants. The contents of “Obeldicks” have been adapted to fit the target population of the study; this intervention aims at increasing the low adherence rates reported in adolescents and adults with obesity [76]. Intervention techniques include motivational interviewing and behavioural training, aiming at changing (family) health behaviour permanently, by methods tailored to the individual requirements. Nutritional education is based on a simple traffic light system [79]. Besides the nutrition education [79, 80], advice for reducing sedentary habits and increasing physical exercise will also be provided by the nurse/clinician caring for the patient. The design of the LIP intervention aims at minimising the daily time burden for participants, and thus at lowering the risk of lack of adherence because the intervention is perceived as too time consuming.

## Objectives {7}

*Primary objective*: To test the efficacy of SPIOMET in normalising ovulation rate in AYAs with PCOS.

*Secondary objectives*: To test the efficacy of SPIOMET in normalising the endocrine-metabolic state, body composition and abdominal fat distribution, on-treatment and post-treatment; to assess the safety of SPIOMET and the adherence and subjective acceptability, as well as the quality of life of participating subjects.

## Trial design {8}

This is a multi-centre, multi-national, randomised, double-blind, placebo-controlled, four-arm parallel group, phase II clinical trial, with 1:1:1:1 allocation ratio. A total of 364 AYAs with PCOS will be randomly assigned to receive:

1. Placebo (*N* = 91)
2. PIO 7.5 mg (*N* = 91)
3. SPI 50 mg + PIO 7.5 mg (SPIO, *N* = 91)
4. SPI 50 mg + PIO 7.5 mg + MET 850 mg (SPIOMET, *N* = 91)

Patients belonging to the four different subgroups will ingest a tablet once daily at dinner time over 12 months and will be followed for another 6 months post-treatment. In addition, all patients will receive the same lifestyle guidance throughout the active treatment phase.

The analysis will be conducted following a hierarchical approach. The multiplicity will be fully controlled, and the alpha level will be therefore maintained at a predefined two-sided 5% level. A fixed-sequence statistical strategy has been planned to test treatment groups in a predefined order, all at the same significance level alpha (e.g. *α* < 0.05), moving to a next comparison only after a statistically significant difference on the previous comparison (https://www.fda.gov/media/102657/download). The following predefined order has been planned:

1. Placebo vs SPIOMET
2. Placebo vs SPIO
3. Placebo vs PIO
4. PIO vs SPIOMET
5. SPIO vs SPIOMET

In this hierarchical approach, the first comparison will be analysed as first, and the next comparison will be analysed only if there is a statistically significant finding for the first comparison (*p* < 0.05). Hence, if there is no significant finding for the first comparison, no formal statistical conclusion can be reached for the subsequent comparisons (2nd, 3rd and 4th), even if there is a large apparent treatment effect. Therefore, the 5th comparison will only be declared as statistically significant, if the previous 1st, 2nd, 3rd and 4th comparisons were to achieve the significant criteria (*p* < 0.05).

Patients will be randomised (1:1:1:1) with stratification by:

1. Clinical site: 7 sites
2. Gynaecological age (years elapsed since menarche): 2–5 years and > 5 years
3. BMI: < 24.9 and ≥ 25.0 kg/m^2^

Additionally, patients will be allocated with a minimisation method (including an element of randomisation) (https://www.fda.gov/media/87621/download).

The trial has been registered in EudraCT under the ID 2021–003177-58 and Clinicaltrial.gov under the ID NCT05394142. The Ethics Committee (EC) of all participating clinical centres approved the trial.

## Methods: participants, interventions and outcomes

### Study setting {9}

The trial will be conducted in a large and ethnically diverse cohort recruited in 7 institutions from 4 European member states and 2 associated countries.

1. Hospital Sant Joan de Déu (HSJD) – Fundació Sant Joan de Déu (FSJD), Esplugues de Llobregat, Barcelona, Spain
2. Hospital de Girona Dr. Josep Trueta – Fundació Institut d’Investigació Biomèdica de Girona (IDIBGI), Girona, Spain
3. Azienda Ospedaliero – Universitaria S. Orsola, di Bologna (UNIBO), Italy
4. Odense Universitets Hospital (UNIODE), Odense, Denmark
5. St. Olavs Hospital – Norwegian University of Science and Technology (NTNU), Trondheim, Norway
6. Division of General Paediatrics, Department of Paediatrics and Adolescent Medicine, Medical University of Graz, and Division of Endocrinology and Diabetology, Department of Internal Medicine, Medical University of Graz, Graz, Austria
7. İstanbul Faculty of Medicine Topkapi – Istambul Universitesi (ISTA), Istanbul, Turkey

### Eligibility criteria {10}

The study population will consist of *N* = 364 AYAs with PCOS. Of those, at least 180 will be adolescent girls (age < 18 years). A tentative clinical diagnosis of PCOS will be performed upon referral of the patient to the participating centre (by primary care paediatricians, family doctors, gynaecologists, or paediatric or adult endocrinologists working in referral hospitals), and inclusion and exclusion criteria will be evaluated, in order to confirm eligibility.

### Inclusion criteria

1. Age range within the AYAs category (> 12.0 years and ≤ 23.9 years at study start) [81]
2. Gynaecological age of 2 years or more
3. Clinical and/or biochemical androgen excess:
  Clinical androgen excess, as defined by the presence of hirsutism (modified-Gallwey score ≥ 4) [3, 4, 82] and/or inflammatory acne (Leeds scale) unresponsive to medications [3, 4, 83, 84] and/or
  Biochemical androgen excess, as defined by increased total testosterone (≥ 45 ng/dL), and/or a free androgen index (FAI) higher than 3.5 [FAI, total testosterone (nmol/L) × 100/SHBG (nmol/L)], in the follicular phase of the cycle (days 3–7) or after 2 months of amenorrhea [3, 13, 85]
4. Menstrual irregularity, as defined by ≤ 8 menses per year corresponding to an average inter-menstrual time of ≥ 45 days [3, 12, 83]
5. Written informed consent obtained from the patient (if aged > 18.0 years), or assent from the patient and consent from the parents or the legally acceptable representative (if aged < 18 years)

### Exclusion criteria

1. Class II obesity or morbid obesity (BMI of 35 kg/m^2^ or higher) according to published international standard charts for both adolescents and young women [73]
2. Underweight (BMI < 18.5 kg/m^2^) or eating disorder (anorexia nervosa)
3. Clinical suspicion or laboratory confirmation of anaemia or bleeding disorder
4. Evidence of thyroid, liver, or kidney dysfunction
5. History of precocious puberty (breast development < 8.0 yr) or precocious menarche (menarche < 10.0 yr) [86]
6. Clinical suspicion of Cushing syndrome
7. Evidence of late-onset adrenal hyperplasia due to 21-hydroxylase deficiency [17-hydroxyprogesterone levels > 200 ng/dL in the follicular phase of the cycle or after 2 months of amenorrhea] [3, 13, 87]
8. Hyperprolactinaemia (due to any cause including breast-feeding)
9. Clinical suspicion or laboratory confirmation of glucose intolerance or diabetes mellitus [88, 89]
10. Use of a medication affecting gonadal or adrenal function, or carbohydrate or lipid metabolism in the previous three months (including OCs)
11. Gynaecological age < 2.0 years
12. Positive pregnancy test
13. Pregnancy risk (failure to guarantee the use of non-hormonal contraception in sexually active subjects)
14. Cardiac failure or history of cardiac failure
15. Hypersensitivity to a study drug or an excipient
16. Clinical suspicion of Addison’s disease
17. Clinical suspicion of any type of acute metabolic acidosis (i.e. lactic acidosis, diabetic ketoacidosis)
18. Diabetic pre-coma
19. Acute conditions with the potential to alter renal function such as dehydration, severe infection, shock
20. Any disorder which may cause tissue hypoxia (especially acute disease or worsening of chronic disease) such as decompensated heart failure, respiratory failure, recent myocardial infarction, shock
21. Acute alcohol intoxication or clinical suspicion of alcoholism
22. Clinical suspicion or laboratory confirmation of hyperkalaemia
23. Concomitant use of eplerenone or other potassium sparing diuretics
24. Concomitant use of potassium-conserving diuretics and potassium supplements
25. Current bladder cancer or a history of bladder cancer
26. Non-investigated macroscopic haematuria

### Who will take informed consent? {26a}

The principal investigator (PI) or their designee at each centre, in accordance with the institutional policy, will obtain an informed consent previously reviewed and approved by the responsible Ethical Committee (EC). A written consent form bearing the full name and signature of the patient or the patient’s parent/guardian and the assent of the patient (in case of minor patients), together with the name, date and signature of the local investigator, will be obtained. The signed informed consent constitutes a confidential document and therefore will be archived in the study binder. A copy of the consent will be given to the patient or patient’s parent/tutor.

### Additional consent provisions for collection and use of participant data and biological specimens {26b}

An additional written informed consent for biological sample collection and use of participant data will be obtained from the trial participants or the patient’s parent/tutor.

### Interventions

#### Explanation for the choice of comparators {6b}

Comparators were chosen according to the paediatric investigation plan (PIP) approved by EMA (002187-PIP01-17, approved on Feb 26, 2021):

- The Placebo arm is required for key comparisons with SPIOMET arm.
- The PIO arm allows to obtain hitherto scarce data with low-dose PIO in monotherapy in adolescent girls.
- The SPIO arm offers low-dose SPI an opportunity to disclose its additional effects, on top of PIO.

Indeed, many aspects of the current protocol design were agreed with PDCO as part of the aforementioned PIP.

#### Intervention description {11a}

The study medication consists of placebo tablets, PIO tablets, SPIO tablets and SPIOMET tablets manufactured by the pharmaceutical company Reig Jofre (Sant Joan Despí, Barcelona, Spain).

SPIOMET tablets have been manufactured with the same composition and process used to produce the SPIOMET tablet tested in phase I study [50]. PIO and SPIO tablets have been produced at the same facilities, using the same process and excipient composition as for SPIOMET, but replacing the amount of deleted active substances (MET 850 mg or MET 850 mg + SPI 50 mg), by an equivalent amount of suitable excipient that is unlikely to affect drug absorption. The proposed excipients are povidone (polyvinylpirrolidone) k-30, microcrystalline cellulose, croscarmellose sodium, polyglykol 4000 PS, magnesium stearate and purified water. Placebo tablets contain only the aforementioned excipients. Tablets containing placebo, PIO, SPIO and SPIOMET have the same size, shape and colour, ensuring the double-blind design of the study. The composition of the medication used in the clinical trial is detailed in Table 3.

**Table 3:** Medication composition

	**Pioglitazone (7.5 mg)**	**Spironolactone (50 mg)**	**Metformin (850 mg)**	**Excipient**
Arm 1** Placebo**	No	No	No	1.1 g
Arm 2** PIO**	Yes	No	No	Up to 1.1 g
Arm 3** SPIO**	Yes	Yes	No	Up to 1.1 g
Arm 4** SPIOMET**	Yes	Yes	Yes	Up to 1.1 g

Patients in each study arm will take a tablet once a day, at dinner time, during meals. If needed, patients can break the tablet to facilitate the intake. There are no safety concerns or specific safety precautions for the intake of the medications. Missed doses should not be compensated. Postponement of medication intake is acceptable for up to 6 h; if postponement is > 6 h, then the dose should be skipped, and treatment should be continued on the next day. Missed doses should be reported in the patient’s diary.

During the active treatment phase spanning 12 months, a specific LIP will be implemented in 5 sessions that will coincide with the quarterly clinical visits (at 0, 3, 6, 9, 12 months), and will be led by a research nurse at each clinical centre.

#### Criteria for discontinuing or modifying allocated interventions {11b}

Study participants are free to withdraw from participation at any time upon request.

Discontinuation of the study intervention will imply discontinuation of the study and thus a dropout, so that any remaining study procedures will not be performed.

All data available until the discontinuation of the study intervention will be collected. If a clinically relevant finding is identified (including, but not limited to changes from baseline) after enrolment, the investigator or qualified designee will determine whether any change in participant management is needed. Any new clinically relevant finding will be reported as an adverse event (AE).

Criteria for discontinuing the allocated intervention are as follows: (1) pregnancy; (2) significant non-compliance with the intervention, for example, no medication intake for 30 consecutive days; in case of AE, patients are allowed to interrupt the treatment for 7 days without being withdrawn from the study; (3) any clinical AE, laboratory abnormality, or other medical condition/situation such that continued participation in the study is unlikely to be in the best interest of the participant; (4) appearance of an exclusion criterion (either newly developed or not previously recognised).

In case of premature termination of study participation, the patient will be re-allocated to the outpatient clinic as a regular patient whenever possible.

#### Strategies to improve adherence to interventions {11c}

A face-to-face medical visit will be scheduled every 3 months for each participating AYA, for a total of 5 visits over 12 months to maintain a close contact, and to monitor adherence until the end of the active treatment phase. Adherence will be calculated as the ratio between the number of tablets prescribed and dispensed for the period between hospital appointments and the number of tablets returned by the patient at the following appointment. In addition, the Intake Table of the Patient Diary will be screened to check whether the aforementioned ratio fits with the information registered by the patient.

The aforementioned LIP sessions will be implemented by a trained research nurse during the same 5 quarterly visits over the same 12 months of active intervention [77]. In order to optimise adherence, the LIP will focus on strengths rather than on weaknesses; such an approach is known to raise treatment adherence [80].

In order to further improve adherence to the pharmacological and LIP interventions, patients will be contacted by phone between clinical visits, in order to check how they are doing.

#### Relevant concomitant care permitted or prohibited during the trial {11d}

Study participants cannot participate concomitantly in another clinical trial.

Prohibited concomitant use/prohibited medication: (1) oral oestroprogestogen contraception prior to (3 months) and during the entire study (18 months); (2) iodinated contrast agents; (3) caution must be exerted with the concomitant use of medications that may cause hyperkalaemia or metabolic acidosis, such as nonsteroidal anti-inflammatory drugs, including selective cyclooxygenase II inhibitors, angiotensin converting enzyme inhibitors, angiotensin II receptor antagonists and diuretics, especially loop diuretics.

#### Provisions for post-trial care {30}

After study completion (18 months), the participants will receive the best available treatment that their physician considers most appropriate.

### Outcomes {12}

#### Primary outcome

The primary outcome will be the number of ovulations on-treatment and post-treatment (ovulatory function) by salivary progesterone analysis for 12 consecutive weeks during two periods on-treatment (months 0–3, and months 9–12), and during one period post-treatment (months 12–15). Information from salivary progesterone and from menstrual calendars (recorded in the patient’s diary) kept by the patients will be combined to infer each ovulation.

#### Secondary outcomes

1. Clinical variables:
  Weight, height, BMI, waist to hip ratio (WHR), systolic blood pressure (SBP), diastolic blood pressure (DBP)
  Hirsutism score (modified Ferriman and Gallwey score) [22, 82]
  Acne score (evaluated using the Leeds Acne Grading Scale) [84]
  Menstrual regularity
2. Endocrine-metabolic variables:
  Circulating androgens: total testosterone, SHBG, FAI (equivalent to free testosterone), and  androstenedione
  Lipids—total cholesterol, low-density lipoprotein (LDL-cholesterol), high-density lipoprotein (HDL-cholesterol), triglycerides
  Insulinaemia: fasting and 2 h after an oral glucose load [oral glucose tolerance test (OGTT)]. Estimation of insulin resistance from fasting insulin and glucose levels using the insulin resistance homeostatic model assessment (HOMA-IR)
  Markers of inflammation and insulin sensitivity: us-CRP, C-X-C motif chemokine ligand-14 (CXCL14), HMW-adip, GDF15
3. Epigenetic variable: circulating microRNA 451a (miR-451a) concentrations
4. Imaging variables:
  Cardiovascular risk: cIMT (ultrasound)
  Body composition: dual-energy X-ray absorptiometry (DXA)
  Abdominal fat distribution (subcutaneous and visceral) and hepatic fat (magnetic resonance imaging, MRI)
5. Lifestyle assessment parameters, including changes in (1) imaging variables; (2) clinical variables; (3) endocrine-metabolic variables; (4) health behaviour assessed through a questionnaire on physical activity, eating behaviour and nutrition adapted from the Health Behaviour in School-aged Children (HBSC) questionnaire; (5) minimisation of adverse side effects and evaluation of the risk of eating disorders assessed through the Eating Disorder Screening questionnaire SCOFF and the first question of the Binge Eating Disorder Screener 7 (BEDS-7); only in case the risk is confirmed, then, the Eating Disorder Examination Questionnaire (EDE-Q) will be used; (6) adherence to the LIP (completion of preparatory task); (7) patient-reported outcomes (PROMs) on HRQoL, assessed through generic short form 36 health survey (SF-36) and PCOS health-related quality of life questionnaires (PCOSQ).
6. Safety variables:
  Laboratory safety variables: blood count (haemoglobin, haematocrit, red blood cell count, white blood cell count, platelet count), electrolyte panel (sodium, potassium, chloride, calcium, phosphorus), urea, alanine aminotransferase (ALT), aspartate aminotransferase (AST), gamma-glutamyltransferase (GGT), creatinine, vitamin B12 and folic acid
  Pregnancy test
  Report of AEs
  Adherence and acceptability:
  Adherence, calculated as the ratio between the number of tablets prescribed and dispensed for the period between two hospital appointments and the number of tablets returned by the patient at the following appointment
  Acceptability of the tablet by the study patients
7. PROMs on HRQoL: assessed through SF-36 and PCOSQ

### Participant timeline {13}

See Table 4.

**Table 4:** Schedule of activities

	**Study months (± 2 weeks)** ^a^							
**Timepoint**	**Screening**	**On-treatment**					**Post-treatment**	
	** − 1**	**0**	**3**	**6**	**9**	**12**	**15**	**18**
**Enrolment**								
Eligibility screen “	X							
Informed consent	X							
Medical history and prior medication^b^	X							
Randomisation^c^	X							
**Interventions**								
**Pharmacological intervention**								
Placebo		X	X	X	X	X		
PIO		X	X	X	X	X		
SPIO		X	X	X	X	X		
SPIOMET		X	X	X	X	X		
**Lifestyle intervention** ^d^								
LIP and LIP-related questionnaires		X	X	X	X	X		
**Assessments**								
**Ovulation assessment** ^e^								
0–3 months—start		X						
0–3 months—stop			X					
9–12 months—start					X			
9–12 months—stop						X		
12–15 months—start						X		
12–15 months—stop							X	
**Clinical variables**								
Weight, height, BMI, WHR, SBP, DBP^f^		X	X	X	X	X	X	X
Hirsutism score		X	X	X	X	X	X	X
Acne score		X	X	X	X	X	X	X
Menstrual regularity		X	X	X	X	X	X	X
**Endocrine-metabolic variables**								
Circulating androgens^g^		X		X		X		X
Lipids^g^		X		X		X		X
Fasting insulin and glycaemia^h^		X	X^h^	X	X^h^	X	X^h^	X
Glucose-stimulated insulinaemia (OGTT)^i^		X				X		X
Markers of inflammation & insulin sensitivity^g^		X		X		X		X
**Epigenetic variables**								
Circulating miR-451a concentrations		X		X		X		X
**Imaging variables** ^j^								
Cardiovascular risk: cIMT (ultrasound)		X				X		X
Body composition: DXA		X				X		X
Abdominal fat distribution and hepatic fat: MRI		X				X		X
**Adherence and acceptability**								
Adherence to treatment		X	X	X	X	X		
Acceptability of the tablet			X			X		
**Safety variables**								
Laboratory safety variables^k^		X		X		X		X
Pregnancy test^l^		X	X	X	X	X		
Adverse events reporting^m^		X	X	X	X	X	X	X
**PROMs on HRQoL** ^n^								
SF-36		X		X		X		X
PCOSQ		X		X		X		X
**Other activities**								
Concomitant medication^o^		X	X	X	X	X	X	X
Patient diary			X	X	X	X	X	X
Online survey (optional)^p^		X						X

^a^1 month is equal to 30 days. Total duration of the clinical study per patient (including 12 months on-treatment and 6 months post-treatment): 18 months

“To verify the inclusion/exclusion criteria, the following parameters will be measured: body mass index (BMI), gynaecological age, hirsutism, acne, menstrual regularity, blood count (haemoglobin, haematocrit, red blood cell count, white blood cell count, platelet count), electrolyte panel (sodium, potassium, chloride, calcium, phosphorus), lipids [total cholesterol, low-density lipoprotein (LDL-cholesterol), high-density lipoprotein (HDL-cholesterol), triglycerides], glucose, insulin, alanine transaminase (ALT), aspartate transaminase (AST), gamma-glutamyltransferase (GGT), creatinine, urea, vitamin B12, folic acid, progesterone, 17-OH-progesterone, thyroid-stimulating hormone (TSH), androgens [testosterone, sex hormone binding globulin (SHBG), free androgen index (FAI), androstenedione, dehydroepiandrosterone-sulphate], prolactin, ultra-sensitive human chorionic gonadotropin (us-HCG). Androgens will be measured in the early follicular phase of the cycle (days 3 to 7) or after 2 months of amenorrhea

^b^Medical history including any clinically significant event during the past 5 years. Only prior medication relevant for in/exclusion criteria and/ or PCOS will be collected

^c^The screening period finalises with randomisation. All patients will be randomised before the basal visit (month 0), including performing any analyses/test related to this visit

^d^A trained research nurse will lead lifestyle intervention in a quarterly session through a Lifestyle Intervention Programme (LIP) and LIP-related questionnaires [self-reported Health Behaviour questionnaire, SCOFF & first question from Binge Eating Disorder Screener 7 (BEDS-7), Eating Disorder Examination Questionnaire EDE-Q (only in case the risk is confirmed)]. Patients can be contacted to check their status by phone between the visits at site

^e^By weekly salivary progesterone measurements for 12 consecutive weeks in three periods

^f^ *BMI* Body mass index, *WHR* Wwaist-to-hip ratio, *SBP* Systolic blood pressure, *DBP* Diastolic blood pressure

^g^Circulating androgens: total testosterone, sex hormone-binding globulin (SHBG), free androgen index (FAI), androstenedione. Lipids: total cholesterol, low-density lipoprotein (LDL-cholesterol), high-density lipoprotein (HDL-cholesterol), triglycerides. Markers of inflammation and insulin sensitivity: ultra-sensitive C-Reactive protein (us-CRP), C-X-C motif chemokine ligand-14 (CXCL14), high-molecular-weight adiponectin (HMW-adip), growth-and-differentiation factor 15 (GDF15)

^h^At visit months 3, 9 and 15, these analyses are to be performed only when they are necessary, namely in the event of clinically relevant hyperglycaemia observed at any time point. For patients with normal glycaemic status, these analyses are not required

^i^ *OGTT*, oral glucose tolerance test. Fasting and 2 h after an oral glucose load

^j^ *cIMT*, carotid intima media thickness; *DXA*, dual X-ray absorptiometry; *MRI*, magnetic resonance imaging

^k^Laboratory safety variables: blood count, electrolyte panel (sodium, potassium, chloride, calcium, phosphorus), alanine transaminase (ALT), aspartate transaminase (AST), gamma-glutamyltransferase (GGT), creatinine, urea, vitamin B12 and folic acid

^l^Highly sensitive serum or standard sensitive urine pregnancy tests

^m^The study clinician or designee team member will record all reportable events from treatment start until 6 months post-treatment

^n^ *PROMs*, patient-reported outcomes; *HRQoL*, health-related quality of life; *SF-36*, short-form 36 health survey; *PCOSQ*, PCOS health-related quality of life questionnaires

^o^Concomitant medication should include both, prescription and over-the-counter medications taken by the patient throughout the study period, including dose, frequency, indication, start and stop dates

^p^Optional survey to answer questions about the study conduct

### Sample size {14}

Numbers of patients in the four study arms have been calculated to attain 80% statistical power at two-sided 5% alpha level [90–92], with an expected dropout rate of 27%. The sample size is based on the two-sample *t*-test (R package “pwr”, functions “pwr.t.test” and “pwr.t2n.test”).

The number of subjects to be included has been determined based on the primary endpoint, namely the number of ovulations over 6 months (from month 9 to month 15). This design will cover to detect a 0.922 mean difference between placebo and SPIOMET arms. The standard deviation of 1.96 is based on previous evidence [14, 15]. The same difference and standard deviation are considered for potentially subsequent hierarchical comparisons (see the section “Trial design {8}”).

A sample size of 364 AYAs with PCOS [at least 180 of whom will be aged < 18.0 years at study start] is required using a 1:1:1:1 allocation ratio for placebo, PIO, SPIO and SPIOMET. The number of participants to be recruited is thus 91 per treatment arm (Table 5). Assuming a 27% dropout rate, approximately 266 patients (~ 66 or 67 per treatment arm) are estimated to complete the study.

**Table 5:** Sample size and minimum detectable differences among comparison groups

Allocation ratio	1:1:1:1	
**Sample size**		
**Total**	**364**	
Placebo	91	
PIO	91	
SPIO	91	
SPIOMET	91	
	**Minimal detectable mean differences** ^a^ ** and common standard deviations**	**Power** ^b^
1. Placebo vs SPIOMET	0.922 (1.96)	80%
2. Placebo vs SPIO	0.922 (1.96)	64%
3. Placebo vs PIO	0.922 (1.96)	51%
4. PIO vs SPIOMET	0.922 (1.96)	41%
5. SPIO vs SPIOMET	0.922 (1.96)	33%

^a^For ovulation rates

^b^Power to detect statistically significant differences within a fixed-sequence multiple testing approach

### Recruitment {15}

A total of 364 AYAs with PCOS will be recruited at the Outpatient Clinic of 7 hospitals from 4 European member states and 2 associated countries; the geographic distribution of the sites ensures ethnic variation.

The number of patients expected to be recruited are respectively:

- *N* = 70 at HSJD – FSJD (Spain)
- *N* = 46 at Hospital de Girona Dr. Josep Trueta – IDIBGI (Spain)
- *N* = 54 at Azienda Ospedaliero-Universitaria S. Orsola, di Bologna (UNIBO) (Italy)
- *N* = 39 at Odense University Hospital—UNIODE (Denmark)
- *N* = 41 at St. Olavs Hospital – NTNU (Norway)
- *N* = 72 at Medical University of Graz (Austria)
- *N* = 42 at Istanbul Faculty of Medicine Topkapi – ISTA (Turkey)

## Assignment of interventions: allocation

### Sequence generation {16a}

Participant girls and young women will be randomly assigned in a 1:1:1:1 ratio to one of the following treatment groups: placebo, PIO, SPIO or SPIOMET. Treatment allocation will be done using Pocock-Simon Minimization Algorithm. Minimisation method ensures balance of treatments among the stratification factors. With this method, girls and young women will be dynamically allocated to one of the treatment arms with the lowest occurrence at the moment of randomisation with a probability of *p* = 0.9. The stratification factors will be clinical site, gynaecological age and BMI.

Medication kits will be packed in boxes with 12 kits per box. For this purpose, kit numbers and treatment arms will be randomly assigned in 12 blocks using PROC PLAN procedure, SAS 9.4.

The randomisation process ensures that all participants and all investigators performing the clinical visits and the assessments will be blinded for the allocated treatment.

### Concealment mechanism {16b}

Treatment allocation will be done via Interactive Web Randomisation System (IWRS) accessible through the electronic Case Report Form (eCRF) and randomisation codes will be generated. Once a patient is randomised to a planned treatment group, medication kits will be delivered according to the allocated treatment at time 0, 3, 6 and 9 months. Therefore, up to 4 medication kits will be assigned per subject. Each medication kit will contain enough medication for 3 months (3 bottles per kit; each bottle containing 35 pills).

### Implementation {16c}

When a patient meets all eligibility criteria, she will be randomised by the principal investigator at each recruiting centre via IWRS, and a randomisation code will be generated. Subsequently, the hospital pharmacy will receive the necessary information for delivering the allocated medication.

## Assignment of interventions: blinding

### Who will be blinded {17a}

The trial is designed as a double-blind study. Participating girls and young women will receive a daily tablet which will be indistinguishable among study groups. The tablets (all with exactly the same size and appearance) will contain placebo or the active compounds (PIO, SPIO or SPIOMET) depending on the study arm to which the patient will be allocated. Study participants, sites and study team members will remain blinded to the treatment allocation. The study blinding will be maintained throughout the clinical trial until data entry and processing are completed and the database has been locked.

### Procedure for unblinding if needed {17b}

In the event of a suspicion of an unexpected serious adverse reaction, the unblinding of the allocation of an individual participant will be requested for regulatory purposes. However, if possible and appropriate, the blinding will be maintained for the investigators and for those who will analyse and interpret the results at the study’s conclusion. The Sponsor will be notified before the blind is broken unless unblinding is required for a medical emergency necessitating knowledge of the blinded medication in the immediate management of the participant’s condition. In the latter case, the investigator will request the unblinding through the IWRS. The timing and rationale for the unblinding must be recorded in the source documentation.

## Data collection and management

### Plans for collection and assessment of outcomes {18a}

#### Collection of outcomes

Screening evaluation (time − 1).

- Study candidates will be recruited at the outpatient clinic of participating hospitals and written informed consent will be obtained. Medical history will be taken, including gynaecological age, menstrual regularity, clinically significant events over the past 5 years and the use of medication related to the PCOS phenotype and/or to the inclusion/exclusion criteria.
- Confirmation of eligibility will require a clinical visit (hirsutism and acne scores; BMI) and blood sampling [to assess blood count, electrolyte panel, lipids, glucose, insulin, ALT, AST, GGT, creatinine, urea, vitamin B12, folic acid, progesterone, 17-OH-progesterone, thyroid-stimulating hormone (TSH), androgens (testosterone, SHBG, FAI, dehydroepiandrosterone-sulphate), prolactin, ultra-sensitive human chorionic gonadotropin (us-HCG)]. Androgens will be measured in the early follicular phase of the cycle (days 3 to 7) or after 2 months of amenorrhea.

Baseline (time 0), on-treatment (3, 6, 9 and 12 months) and post-treatment (15 and 18 months) evaluations.

- Saliva samples for ovulation assessment (by progesterone measurement) will be collected weekly between 0 and 3 months (for 12 consecutive weeks during months 1, 2 and 3), between 9 and 12 months (for 12 weeks during months 10, 11 and 12) and between 12 and 15 months (for 12 weeks during months 13, 14 and 15).
- Clinical features will be collected at each time point (height and weight to infer BMI; waist and hip circumference to infer WHR; SBP and DBP; hirsutism and acne scores; menstrual regularity) besides information on concomitant medication and adverse events.
- At time 0, 6, 12 and 18 months, blood sampling will be performed in the fasting state to assess endocrine-metabolic, epigenetic and laboratory safety variables. At time 0, 12 and 18 months, additional blood sampling will be performed after 2 h of glucose upload for insulin and glycaemia assessment. At time 3, 9 and 15 months, blood sampling (for fasting insulin and glycaemia assessment) will be performed only in the event of clinically relevant hyperglycaemia observed at any time in the prior months.
- At time 0, 12 and 18 months, ultrasound, DXA and MRI will be performed for assessments of respectively cIMT, body composition, and partitioning of abdominal fat, including hepatic fat.
- Adherence to treatment will be assessed at each time point from baseline to time 12 months; whereas acceptability of the tablet will only be recorded at time 3 and 12 months.
- A highly sensitive serum or standard sensitive urine pregnancy test will be performed at each time point from baseline to time 12 months.
- At each time point from baseline to time 12 months, will attend to a LIP session, and fill in LIP-related questionnaires.
- At time 0, 6, 12 and 18 months, PROMs on HRQoL will also be assessed.
- Between hospital visits throughout the 18 months of study, the participants will record—in a diary—their menses, the time of any medication intake and any adverse event. Participants will deliver this diary at the next visit.
- At time 0 and 18 months, an online survey will collect information on the patient’s experience, respectively, during the enrolment process and throughout the 18 months of the actual study.

##### Saliva sampling

Saliva samples will be collected once weekly, on the same weekday, in the morning and in the fasting state, for 12 consecutive weeks during two timespans on-treatment (0–3 and 9–12 months) and one timespan post-treatment (12–15 months), yielding a total of 36 samples per study participant. The collection of saliva will be performed by spitting in a plastic container that will then be placed in a home freezer space (at − 20 °C), bearing a code number and also the date and time of sample collection. Samples will be kept frozen at home until transportation to the clinical centre, where samples will be processed and stored at fridge or freezing temperature. Once or twice yearly, samples will be shipped to HSJD where salivary progesterone concentrations will be measured.

##### Blood sampling

20 mL of peripheral blood will be sampled from each patient at times 0, 6, 12 and 18 months. During the OGTT at times 0, 12 and 18 months, an additional amount of 10 mL will be drawn 120 min after the glucose load. Collected blood samples will be stored at − 80 °C until shipment to UNIBO and HSJD for analysis (see below).

#### Assessment of outcomes

##### Ovulatory function

Salivary progesterone will be centrally analysed at HSJD using an Enzyme-Linked Immunosorbent Assay (ELISA) [IBL-TECAN, Hamburg, Germany; intra- and inter-assay coefficients of variation (CVs) of 4.9 and 6.7%, respectively; salivary progesterone in the luteal phase, range: 87–550 pg/mL].

Saliva samples are a non-invasive, feasible method for monitoring progesterone levels across the menstrual cycle as they can be self-collected at home and stored in a home freezer for months before analysis [93]. Salivary progesterone concentrations are closely related to unbound serum progesterone [94]. This method has been previously used to assess ovulation rate in adolescents with type 1 diabetes and polycystic ovarian morphology [95, 96]; in one pilot study performed in HSJD, serum progesterone assessed by immuno-chemiluminescence, and salivary progesterone measured by the proposed method were highly correlated (*r* = 0.84; *p* < 0.0001; unpublished results). Two pilot studies performed in adolescent girls with PCOS have confirmed the reliability of salivary progesterone measurements [14, 15].

Information from salivary progesterone measurements and from menstrual diaries will be combined to infer ovulations and annualised ovulation rates. In the two previous pilot studies performed at HSJD [14, 15], with a similar ELISA method no longer available (Novatec, Immundiagnostica, Dietzenbach, Germany; intra- and inter-assay CVs < 10%), candidate ovulations were identified using a lower salivary progesterone cut-off of 99 pg/mL, as recommended by the manufacturer. All the ovulations that were confirmed by the above-mentioned two-step process were associated with salivary progesterone concentrations > 150 pg/mL. The progesterone cut-off derived from the ELISA assay to be used in the present study will be updated/rewritten when the new assay/method has been tested/validated. Candidate ovulations will be confirmed if the menstrual calendar mentions that menses did occur at least 2 weeks before and at most 2 weeks after obtaining the concerned saliva sample.

##### Clinical variables

In each centre, the same investigator will

- measure height and weight to infer BMI
- measure waist and hip circumference to infer WHR
- measure SBP and DBP with an electronic sphygmomanometer on the right arm after a 5-min rest, with the participant supine
- assess hirsutism by modified Ferriman-Gallwey score [22, 82] and acne by Leeds grading scale [84]
- take a history including information about menstrual regularity and the intake of concomitant medication

##### Endocrine-metabolic markers

1. *Androgens:* for enrolment purposes, circulating testosterone, SHBG, androstenedione and dehydroepiandrosterone-sulphate will be measured in referring/participating centres, and FAI will be derived. At times 0, 6, 12 and 18 months in the actual study, SHBG will be measured in the patient’s centre (by immuno-chemiluminiscence) whereas the steroid profiling (including testosterone and androstenedione) will be performed by liquid chromatography-tandem mass spectrometry (LC–MS/MS) at UNIBO [85, 97].
2. *Lipids:* total cholesterol, LDL-cholesterol, HDL-cholesterol and triglycerides will be assessed in the patient’s centre by molecular absorption spectrometry.
3. *Fasting insulin and glycaemia, and glucose-stimulated insulinaemia (OGTT):* insulin resistance will be estimated from fasting insulin and glucose levels with the homeostasis model assessment (HOMA-IR; CIGMA calculator programme v2.00) [14, 15, 98]. Standard OGTTs (75 g of glucose) will be performed after an overnight fast, as recommended [99]. Blood will be sampled before and 2 h after glucose intake for both glucose and insulin measurements, and their *Z*-scores will be derived [14, 15, 27]. Glucose will be measured by the glucose oxidase method and insulin by immuno-chemiluminiscence.
4. *Markers of inflammation and insulin sensitivity:*
  Serum us-CRP levels will be measured by immuno-chemiluminescence at participating centres.
  Circulating CXCL14 will be measured by a specific ELISA kit (RayBiotech, Nocross, GA, USA) with a sensitivity of 0.7 ng/mL, and with intra- and inter-assay CV < 12% (74).
  Circulating HMW-adip will be measured by ELISA (R&D Systems, Minneapolis, MN, USA) with intra- and inter-assay CVs < 9%, as reported [14, 15].
  Circulating GDF15 will be measured by a specific human ELISA kit (R&D Systems, Minneapolis, MN, USA) with intra- and inter-assay CVs < 6%, as reported [100].

Measurements of HMW-adip, CXCL14 and GDF15 will be centralised at HSJD.

##### Epigenetic variables

Expression levels of circulating miR-451a will be measured by a RT-qPCR-based approach [101], centralised at HSJD.

##### Imaging variables

1. *Cardiovascular risk:* cIMT is a non-invasive marker of cardiovascular risk. At each centre, ultrasound scans of the carotid arteries will be obtained by the same investigator (blinded to treatment allocation) and with comparable high-resolution equipment and transducers; images will be read by the same investigator (blinded to treatment allocation), stored at a central repository, and centrally validated at HSJD. The values obtained on each side will be averaged (the intra-observer CVs are expected to be < 10%).
2. *Body composition:* bone mineral density (BMD), bone mineral content (BMC) and body composition [fat body mass (FBM), truncal fat mass (TFM), abdominal fat mass (AFM), lean body mass (LBM)] will be assessed by DXA with comparable equipment in the participating centres and with previous phantom calibration [14, 15] and will in each centre be analysed by the same investigator (blinded to treatment allocation). Images will be stored at a central repository and will be centrally validated at HSJD.
3. *Abdominal fat distribution and hepatic fat: a*bdominal fat distribution (subcutaneous and visceral fat) and hepatic fat will be analysed by MRI using comparable equipment in the participating centres. At each centre, scans will be performed by the same operator (blinded to treatment allocation), will be anonymised by the data management team (ensuring blindness to all clinical information) and will then be analysed centrally at HSJD.

A central reader (based at HSJD) will handle all imaging data and will maintain a central repository (based at QUIBIM, Valencia, Spain) wherein the images will be available for analysis.

##### Lifestyle

The effectiveness of lifestyle intervention will be evaluated by assessing changes in:

- Imaging parameters (by DXA, MRI and cIMT)
- Clinical variables (including BMI)
- Endocrine-metabolic variables (including lipids and usCRP)
- Health behaviour assessed through a questionnaire on physical activity, eating behaviour and nutrition [questions adapted from HBSC questionnaire [102]]
- Minimisation of adverse side effects, mainly aggravation of unfavourable eating behaviour [103] and evaluation of the risk of eating disorders assessed through the SCOFF and the first question of BEDS-7; only in case the risk is confirmed, then, the EDE-Q will be used which covers the four subscales of restraint, eating concerns, weight concern and shape concern [104].
- Adherence to LIP (completion of session preparation task).
- PROMS on HRQoL [generic (SF-36) and specific (PCOSQ) questionnaires (see below)].

##### Safety variables assessment

1. *Laboratory safety variables:* blood count (by flow cytometry and colorimetric assay), electrolyte panel, ALT, AST, GGT, urea, creatinine (by molecular absorption spectrometry), vitamin B12 and folic acid (by immuno-chemiluminescence) will be assessed locally at each participating hospital.
2. *Pregnancy test:* before treatment start, a highly sensitive serum HCG pregnancy test will be performed. During the study, pregnancy tests will be performed 3-monthly, either by highly sensitive serum test, or by a standard sensitive urine test detecting HCG concentrations > 25 mIU/mL.
3. *Report of AEs:* all AEs will be recorded in the medical history and subsequently in the eCRF. The investigator will also judge whether the adverse event is related or not to the study drug, and this judgement will also be noted in the medical history and eCRF.

##### Adherence and acceptability

1. *Adherence:* as a marker of adherence to intervention, the ratio will be calculated (and expressed in %) between the number of tablets that were ingested and those that were prescribed, the former being calculated as the difference between the number of tablets that were dispensed and those that were returned at the subsequent visit. In addition, the Intake Table of the Patient Diary will be screened to check whether the calculated ratio (or %) fits with the information registered by the patient.
2. *Acceptability of the tablet:* the patient’s opinion regarding the palatability and easiness to swallow the tablet (containing Placebo, PIO, SPIO or SPIOMET) will be recorded by using a simple questionnaire with numerical rating scales, adapted from reference [105] and shown at Table 6.

**Table 6:** Tablet acceptability questionnaire

Numerical Rating Scales (NRSS) OR qualitative/descriptive controlled vocabulary for palatability/acceptability assessments of paediatric oral use formulations					
**Score**	**1**	**2**	**3**	**4**	**5**
Overall opinion of taste	Very bad	Bad	Acceptable/ OK/ Not nice and not bad	Good/ Nice	Very good/ Very nice
Overall swallowability (descriptive)	Vomits the drug immediately or within 2 h	Spat it out	Swallowed with some problems	Swallowed with no problems	
Parent/caregivers’ overall opinion on their daughter’s acceptability of the formulation	Very bad	Bad	Acceptable/ OK	Good	Very good

##### PROMs and HRQoL

The effects of PROMs on HRQoL will be assessed (at times 0, 6, 12 and 18 months) with generic (SF-36) and specific (PCOSQ) questionnaires. SF-36 consists of 36 items grouped in 8 multi-item scales. The scores on each domain range from 0 to 100; the higher scores point to a better condition using the same 8 subscales. The PCOSQ contains 26 items scoring the same 8 domains. Each item is answered by choosing from a Likert scale with 7 options, from 1 (always) to 7 (never). Questionnaire versions in languages of the participating centres/countries will be validated during the clinical trial.

### Plans to promote participant retention and complete follow-up {18b}

Participants will be seen in person during 3-monthly medical visits. Between such appointments, the study centre will stay in contact with participants (by phone) as to ensure compliance with treatment and follow-up.

If a study participant skips one study visit, then

- The clinical site (investigator or designee) will make a first attempt to contact the participant, will point to the importance of the study schedule and will make a new appointment at mutually earliest convenience, unless the participant clearly expresses the wish to abandon the study.
- If a first attempt to contact the participant fails, then at least three further attempts will be made by phone, by certified letter to the participant’s last known mailing address, or by equivalent routes. These contact attempts will be documented in the participant’s medical record or study file.
- If the participant cannot be reached by the staff of the study centre, or ends up skipping the next visit too, then she will be considered to have withdrawn from the study, due to loss of follow-up.

### Data management {19}

The following data will be collected in this trial:

- Clinical and endocrine-metabolic data: numerical data obtained by the staff during the clinical visits or blood tests and collected through the eCRF.
- Imaging data (DICOM): will be obtained by analysis of ultrasound, DXA and MRI data. All images will be sent electronically to an online repository (QUIBIM).
- Information from patient diary and questionnaires (HBSC, SCOFF, BEDS-7, EDE-Q, SF-36, PCOSQ and acceptability of the tablet questionnaire) will be collected on paper and transcribed to the eCRF by the investigators or their designees.

Participating girls and young women will be assigned a unique identification code (without any reference to their name), and all patient data will be recorded in an anonymised manner using this code. The list with identification codes will remain at each recruiting centre and will be stored separately from the trial data. Only if needed will this list be used for the re-identification of patients.

The research data of the study will be collected in the Open Clinica eCRF (OpenClinica v3.15.3 system). The investigator(s) or designee(s) will be responsible for the data entry through the eCRF system. The access to the eCRF will be controlled by username and password. Any change conducted in the database will be registered by means of an audit trail. The reason for accessing, username, date and time, table name, old and new values and subject/patient ID will be recorded. Any data transfer will be done using Secure Sockets Layer (SSL) connection with encryption.

For the SPIOMET4HEALTH clinical trial, the Clinical Research Organization (CRO) Optimapharm (Palma de Mallorca, Spain) will be responsible for all tasks related to data management.

### Confidentiality {27}

The study protocol, documentation, data and all other information generated will be held in strict confidence. No information concerning the study or the data will be released to any unauthorised third party without prior written approval of the Sponsor.

All research activities will be conducted in as private a setting as possible.

The study monitor, other authorised representatives of the Sponsor, representatives of the ECs, regulatory agencies or pharmaceutical company supplying study product will inspect all documents and records required to be maintained by the investigator, including but not limited to, medical records (office, clinic, or hospital) and pharmacy records for the participants in this study. The clinical study site will allow access to such records after patients’ written consent.

The study participant’s contact information will be securely stored at each clinical site for internal use during the study. At the end of the study, all records will continue to be kept in a secure location for as long a period as dictated by the reviewing EC, institutional policies or sponsor requirements.

Study participant research data, which are for purposes of statistical analysis and scientific reporting, will be transmitted to and stored at the Optimapharm Biometry Department. This will not include the participant’s contact or identifying information. Rather, individual participants and their research data will be identified by a unique study identification number. The study data entry and study management systems used by clinical sites and by Optimapharm Biometry Department research staff will be secured and password protected. At the end of the study, all study databases will be de-identified and archived at the Optimapharm Biometry Department.

### Plans for collection, laboratory evaluation and storage of biological specimens for genetic or molecular analysis in this trial/future use {33}

Circulating testosterone and androstenedione, circulating markers (HMW-adip, CXCL14, GDF15 and miR-451a) and salivary progesterone will be analysed centrally at UNIBO and HSJD respectively, and samples will thus be stored in those centres until assessment. The remnant samples will be stored at Biosample Collection located at the Endocrinology Laboratory of HSJD (Barcelona, Spain). These samples can be used in future studies related to PCOS.

## Statistical methods

A detailed Statistical Analysis Plan (SAP) will be developed for this study. The SAP will provide full details of the analyses, the analysis populations, the data displays and the algorithms to be used for data derivations. The SAP will be finalised and approved prior to the database lock.

### Statistical methods for primary and secondary outcomes {20a}

The statistical objective of the study will be the assessment of the efficacy, tolerability and safety of SPIOMET in AYAs with PCOS. As explained (see the “Trial design {8}” section), the statistical analysis will be conducted with a hierarchical approach considering the mean number of ovulations (primary outcome) for the different study treatment arms following the predefined order: (1) SPIOMET vs placebo; (2) SPIO vs placebo; (3) PIO vs placebo; (4) SPIOMET vs PIO; and (5) SPIOMET vs SPIO. Therefore, the first comparison will be analysed in the first place, and only if a significant finding is detected for this comparison, a formal statistical conclusion will be reached for the subsequent comparisons (2nd, 3rd, 4th and 5th).

Each null hypothesis will be tested in all randomised patients based on the intention-to-treat (ITT) principle. The analysis will be conducted using an analysis of variance (ANOVA) considering the stratification factors used in the randomisation.

The statistical analysis will be carried out in accordance with the principles specified in the International Conference on Harmonisation (ICH) Topic E9 (CPMP/ICH/363/96) [106] and the addendum on estimands [107]. The SAS System [108] (release 9.4, or an upgraded version), or equivalent validated statistical software, will be used to analyse the data sets. Statistical tests for primary and secondary efficacy outcomes will use a significance level of 0.05; point estimates and two-side 95% confidence intervals for appropriate treatment effects will be provided.

#### Analysis of the primary outcome

To calculate individual ovulation rates, the number of ovulatory cycles in each girl over the assessment period will be compared with the maximum possible number of ovulatory cycles over that period. The number of ovulations between times 0 and 3 months is expected to provide evidence that the study subgroups have a comparably low number of ovulations shortly after study start. Although it may have been statistically preferable to assess ovulatory function prior to randomisation, we elected to do it shortly thereafter because clinical experience indicated that a 3-month delay of randomisation and treatment could be a cause of non-enrolment. The number of ovulations between 9 and 12 months (on-treatment) and 12 and 15 months (post-treatment) will be combined and analysed using ANOVA, considering the stratification factors used in the randomisation. A secondary analysis per 3-month timespan will also be performed. To calculate individual ovulation rates, the number of ovulatory cycles in each girl over a period of 12 consecutive weeks will be compared with the maximum possible number of ovulations over that period, namely, *N* = 3. We will use number of on-treatment and post-treatment ovulations rather than the classification into oligo-ovulatory and normo-ovulatory status because the cut-off between these two conditions is debatable due to the wide range of prevalence of ovulatory estimands in the early post-menarcheal years [109].

#### Analysis of secondary outcomes

For the many different secondary endpoints, the analyses will be conducted according to the following strategy: Fisher’s exact test to compare categorical variables, ANOVA for continuous variables, and in case of deviations of the applicability assumptions, we will use non-parametric methods (Mann–Whitney test). Continuous variables following a Gaussian distribution repeated over time (longitudinal data) will be analysed using mixed models for repeated measures [longitudinal mixed model for repeated measurements (MMRM)] and variables not meeting the parametric assumptions will be rank transformed. Binary longitudinal data will be analysed similarly with marginal models [Generalised Estimating Equation (GEE)].

### Interim analyses {21b}

No efficacy interim analyses are planned for this study. Primary and secondary endpoints will be analysed based on the stratification factors applied to the randomisation once the trial is finished.

### Methods for additional analyses (e.g. subgroup analyses) {20b}

No subgroup additional analyses will be performed.

### Methods in analysis to handle protocol non-adherence and any statistical methods to handle missing data {20c}

The handling of missing data will follow the principles specified in the ICH Topic E9 (CPMP/ICH/363/96), ICH E9 [110] and guidance on missing data [111] guidelines. The main analysis will include all randomised patients following the ITT principle, and thus, patients not completing the 6 months’ assessment period for the primary endpoint will be imputed. If patients have completed ≥ 80% of the 6 months assessment, then the mean value will be imputed for the rest of the missing observations. Otherwise, the 0 to 3 months of follow-up initial period after randomisation will be used for the imputations.

### Plans to give access to the full protocol, participant-level data and statistical code {31c}

The full protocol, participant-level data and statistical code will be provided on acceptable demand.

## Oversight and monitoring

### Composition of the coordinating centre and trial steering committee {5d}

The clinical trial is part of the project SPIOMET4HEALTH funded by the European Union’s Horizon 2020 research and innovation programme. SPIOMET4HEALTH will be carried out by a consortium of groups working on PCOS in AYAs. Besides the 7 hospitals where the clinical data will be generated, 10 partners will be part of the consortium: KU Leuven (KUL, Leuven, Belgium), Reig Jofre (Sant Joan Despí, Barcelona, Spain), Optimapharm (Palma de Mallorca, Spain), Vestische Children’s Hospital Children’s Hospital/University of Witten/Herdeck (Witten, Germany), Asphalion (Barcelona, Spain), Outcomes’10 (Castellón de la Plana, Spain), Zabala Innovation (Pamplona, Spain), Universidad Complutense de Madrid (Madrid, Spain), European Institute of Women’s Health (EIWH, Dublin, Ireland), EU delegation of Make Mothers Matter (MMM, Brussels, Belgium).

HSJD – FSJD will be responsible for the coordination of SPIOMET4HEALTH. KU Leuven will function as co-coordinator and will be responsible for the intellectual property management. Reig Jofre will be responsible for manufacturing the study medication. The CRO Optimapharm will lead the management of the clinical trial activities and the Vestische Children's Hospital Children's Hospital/University of Witten/Herdeck will be responsible for the development and implementation of the LIP. Asphalion will perform the regulatory and exploitation activities to define the roadmap towards a marketing authorisation of SPIOMET for the treatment of adolescent PCOS. Outcomes’10 and Universidad Complutense de Madrid will lead the analysis of the costs and health effects of each treatment option as HRQoL. The associations EIWH and MMM will engage activities, stakeholder networks and mapping of current policies on PCOS at national and European levels. Zabala Innovation will be responsible for communication and dissemination of the project.

The trial Sponsor and coordinating centre will be HSJD-FSJD. The General Assembly involves all 17 members of the consortium, will meet at least once yearly and will be the consortium’s ultimate decision-making body. The trial Steering Committee (SC) will be composed of the coordinator (HSJD-FSJD), the co-coordinator (KUL) and a core group composed of UNIBO, Optimapharm and Asphalion. The SC will be responsible for decision-making on the daily running of the project and will meet 3-monthly. The principal investigators as well as the research coordinators at each recruiting centre will meet once monthly to coordinate and supervise all the tasks. At each clinical site, a group of nurses, clinicians and researchers will provide day-to-day support for the trial.

### Composition of the data monitoring committee, its role and reporting structure {21a}

The CRO Optimapharm will be responsible for the trial monitoring, data quality, safety and pharmacovigilance. In addition, an independent Data Safety and Monitoring Board (DSMB) will be established to monitor data quality and patient safety as the study progresses. In order to do so, a DSMB may review unblinded study information (on a patient level or treatment group level) during the conduct of the study. Based on its review, the DSMB provides the Sponsor with recommendations regarding study modification (for example, to strengthen the scientific validity of the trial), continuation or termination. The DSMB will be composed of one external statistician/epidemiologist and two external medical expert consultants.

### Adverse event reporting and harms {22}

The nature, severity, treatment and outcome of any potential AE or serious AE (SAE) will be recorded by the investigator or designee team member at each clinical site and will determine the relationship to the treatment or underlying disease. The investigator will collect any unfavourable and unintended sign, symptom or disease temporally associated with the use of the investigational product, whether related to the investigational product or not. All AEs and SAEs, whether expected or unexpected, will be recorded in the medical history and subsequently in the eCRF. The Sponsor will be responsible for determining whether an AE is expected or unexpected. The study clinician or designee team member will record all reportable events from treatment start until 6 months post-treatment.

At each study visit, the investigator will inquire about the occurrence of AE/SAEs since the last visit. Events will be followed for outcome information until resolution or stabilisation. If a participant is found to be pregnant or may have been pregnant at the time of exposure to the tested drugs, then the pregnancy, any AEs associated with maternal exposure and the pregnancy outcome will be recorded on the Pregnancy Surveillance Form and reported to Optimapharm and the Sponsor.

### Frequency and plans for auditing trial conduct {23}

The CRO Optimapharm will be responsible for the auditing of the SPIOMET4HEALTH trial and will conduct the following types of site visits: (1) site initiation visits; (2) monitoring visits; (3) close-out visits. The first one-site monitoring visit will be conducted at each site within 6 weeks after the first subject is randomised. Monitoring frequency will be every 3 months. After the completion of each visit, a report will be provided.

### Plans for communicating important protocol amendments to relevant parties (e.g. trial participants, ethical committees) {25}

Protocol modifications will be approved by the EC of each recruiting centre and the National Competent Authority in each country.

#### Dissemination plans {31a}

The results of the clinical trial will be disseminated at two levels:

- Patients with PCOS and their families, including patient associations and general public (press release, informative talks, web, radio, television)
- Clinicians, researchers and academic staff (articles in peer-reviewed journals, presentations at national and international meetings)

## Discussion

### Innovation and originality

Worldwide, PCOS is the most prevalent endocrine-metabolic disorder in women of reproductive age, and it is the leading cause of anovulatory subfertility. There is no approved therapy for PCOS in AYAs. New insights into the pathophysiology suggest that PCOS is essentially the result of a mismatch between early adipogenesis and later lipogenesis resulting in an excess of fat in subcutaneous adipose tissue and in an abundance of lipids in ectopic depots such as liver and viscera. The present trial will test—on top of lifestyle measures—the efficacy and safety of a randomised treatment with low-dose generics (PIO, SPIO, SPIOMET) that aim to reduce ectopic fat, to revert the entire PCOS phenotype (and its correlates) towards normal and, ultimately, to decrease PCOS’ economic burden on health care systems.

### Strengths and limitations

The strengths include the double-blind, placebo-controlled design of a first multi-centre study in an ethnically diverse study population that bridges adolescence and early adulthood; the combination of a specific lifestyle intervention and different pharmacological treatments; the phase of post-treatment follow-up including an evaluation of ovulatory function; and the pre-, on- and post-treatment assessments with up-to-date markers, imaging techniques and questionnaires. The main limitations relate to the relatively narrow age range, and the exclusion of patients with obesity beyond class I.

## Trial status

Protocol version 4; 15 November 2022. Recruitment start date: May 2022. Expected completion date: April 2024.

### Supplementary Information

**Additional file 1.** Original funding documentation.

**Additional file 2.** Ethical approval documents.

## Data Availability

The study investigators will have access to the final dataset, and they will not have any contractual agreement that limits such access. Any data required to support the protocol can be supplied on request.

## Abbreviations

AE: Adverse event
AFM: Abdominal fat mass
ALT: Alanine aminotransferase
AMPK: AMP-activated protein kinase
ANOVA: Analysis of variance
AST: Aspartate aminotransferase
AYA: Adolescent and young adult
BAT: Brown adipose tissue
BEDS-7: Binge Eating Disorder Screener 7
BMC: Bone mineral content
BMD: Bone mineral density
BMI: Body mass index
CDK5: Cyclin-dependent kinase 5
cIMT: Carotid intima-media thickness
CRO: Clinical Research Organization
CV: Coefficient of variation
CXCL14: C-X-C motif chemokine ligand-14
DBP: Diastolic blood pressure
DSMB: Data Safety and Monitoring Board
DXA: Dual-energy X-ray absorptiometry
EC: Ethic Committee
eCRF: Electronic Case Report Form
EDE-Q: Eating Disorder Examination Questionnaire
EIWH: European Institute of Women’s Health
ELISA: Enzyme-linked immunosorbent assay
EMA: European Medicine Agency
FAI: Free androgen index
FBM: Fat body mass
FDA: US Food and Drug Administration
FDC: Fixed-dose combination
FSJD: Fundació Sant Joan de Déu
GDF15: Growth-and-differentiation factor 15
GEE: Generalised estimating equation
GGT: Gamma-glutamyltransferase
HBSC: Health Behaviour in School-aged Children
HCG: Human chorionic gonadotropin
HDL: High-density lipoprotein
HMW-adip: High molecular weight adiponectin
HRQoL: Health-related quality of life
HSJD: Hospital Sant Joan de Déu
ICH: International Conference on Harmonisation
IDIBGI: Institut d’Investigació Biomèdica de Girona
IR-HOMA: Insulin Resistance Homeostatic Model Assessment
ISTA: Istambul Universitesi
ITT: Intention-to-treat
IWRS: Interactive Web Randomisation System
KUL: Katholieke University of Leuven
LBM: Lean body mass
LC–MS/MS: Liquid chromatography-tandem mass spectrometry
LDL: Low-density lipoprotein
LIP: Lifestyle Intervention Programme
MET: Metformin
miR-451a: MicroRNA 451a
MMM: Make Mothers Matter
MMRM: Mixed Model for Repeated Measurements
MRI: Magnetic resonance imaging
MUG: Medizinische Universität Graz
NTNU: Norwegian University of Science and Technology
OC: Oral contraceptive
OGTT: Oral Glucose Tolerance Test
PCOS: Polycystic ovary syndrome
PCOSQ: PCOS Health-Related Quality of Life Questionnaire
PDCO: Paediatric Committee of the European Medicine Agency
PI: Principal Investigator
PIO: Pioglitazone
PIP: Paediatric Investigation Plan
PPAR: Peroxisome proliferator-activated receptor
PROMs: Patient-reported outcomes
SAE: Serious adverse event
SBP: Systolic blood pressure
SC: Steering committee
SCOFF: Sick-Control-One-Fat-Food
SF-36: Short-Form 36 Health Survey
SHBG: Sex hormone-binding globulin
SPI: Spironolactone
SPIOMET: Spironolactone, pioglitazone and metformin
SSL: Secure Sockets Layer
T2D: Type 2 diabetes
TFM: Truncal fat mass
TSH: Thyroid-stimulating hormone
TZD: Thiazolidinedione
UNIBO: Università di Bologna
UNIODE: Odense Universitets Hospital
us-CRP: Ultra-sensitive C-reactive protein
us-HCG: Ultra-sensitive human chorionic gonadotropin
WHR: Waist to hip ratio
