# Supplementary material for: SPIOMET4HEALTH—efficacy, tolerability and safety of lifestyle intervention plus a fixed dose combination of spironolactone, pioglitazone and metformin (SPIOMET) for adolescent girls and young women with polycystic ovary syndrome: study protocol for a multicentre, randomised, double-blind, placebo-controlled, four-arm, parallel-group, phase II clinical trial
Source: Trials. 2023 Sep 15;24:589. doi: 10.1186/s13063-023-07593-6 (PMC10503102; doi:10.1186/s13063-023-07593-6)
Supplement: Supplementary file 1 — Additional file 1. Original funding documentation. [file 13063_2023_7593_MOESM1_ESM.pdf]

EUROPEAN COMMISSION
DIRECTORATE-GENERAL FOR RESEARCH AND INNOVATION

RTD.E – People
E.2 – Combatting Diseases

Lourdes IBAÑEZ
HOSPITAL SANT JOAN DE DÉU
PASEO SANT JOAN DE DEU 2
08950 ESPLUGUES DE LLOBREGAT
SPAIN

Subject: Horizon 2020 Framework Programme
Call: H2020-SC1-2020-Two-Stage-RTD
Project: 899671 — SPIOMET4HEALTH
GAP invitation letter

Dear Madam/Sir,

I am writing in connection with your proposal for the above-mentioned call.

Having completed the evaluation, we are pleased to inform you that your proposal has passed this phase and that we would now like to start **grant preparation**.

Please find enclosed the evaluation summary report (ESR) for your proposal (for both stages of the evaluation). It is based on the comments and opinion of independent outside experts that helped us with the evaluation.

Please be aware that there may be differences between the ESRs, since stage 1 evaluations are done on the outline of your proposal, while stage 2 evaluations cover the full proposal.

Invitation to grant preparation

Grant preparation will be based on the following:

1. **Project:** 899671 — SPIOMET4HEALTH

2. **Topic:** SC1-BHC-08-2020 — New interventions for Non-Communicable Diseases

3. **Type of action:** Research and Innovation action

4. **Project officer:** Amalia Irina VLAD
   Combatting Diseases

   ⚠ Please always use the Funding & Tenders Portal messaging function (via your Portal account). Do NOT contact us via other means (email, letter, etc.) — unless explicitly asked to do so.

5. **Maximum grant amount:**

   Requested EU contribution (according to proposal): 6 366 148.50 EUR

   Maximum grant amount (proposed amount, after evaluation): 6 366 148.50 EUR

6. **Project duration:** 60 months

7. **Grant preparation:**

   Preparation of grant data & annexes: 3 weeks after receiving this letter

   ⚠ The annexes (description of the action, estimated budget, etc.) must be based on the proposal you submitted and the clarifications provided (if any). You may normally NOT make changes to the project/project budget/ consortium composition (except if required by us). Please immediately inform the project officer if you need to make a change *(e.g. bankruptcy, etc.)*.

   ⚠ Please be aware that your proposal may still need to undergo an ethics review or security scrutiny (and the results will then have to be implemented by you).

   Once we have checked the information you have encoded, you will have 2 weeks to submit your final version — to bring it in line with our comments.

   Declaration of honour (DoH): 5 weeks after receiving this letter

   ⚠ Please note that each applicant should also submit a signed and scanned DoH for each one of their linked third parties.

   Signature: within 3 months after receiving this letter (planned date)

   ⚠ Please note that repeated failure to respect deadlines during grant preparation may lead to the rejection of your proposal (or a consortium member). Lack of cooperation will be taken to mean that you are no longer interested.

8. **Funding & Tenders Portal**

   Grant preparation (including signature) will be done exclusively through the Funding & Tenders Portal electronic exchange system (login via your Portal account). Do NOT contact us via other means (email, letter, etc.) — unless explicitly asked to do so.

   Please be aware that linked third parties (if allowed) must be registered and validated as legal entities in the Portal Participant Register. ⚠ Register them immediately, if not already done.

   Please note that some of your legal and financial data in the Participant Register is read-only and can be updated only by a LEAR (via the Portal My Organisation(s) page). You will therefore be contacted soon to appoint a LEAR (unless you already have one).

9. **Other**

   ⚠ The ethics review for your proposal is still ongoing. The results will be made available in the Portal during grant preparation. Please be aware that they may require action *before* signature of the agreement.

   ℹ For more information on grant preparation, *see the Online Manual*. You can refer to this document also for programmes other than H2020 since the procedures are very similar.

⚠ Please note that this letter does **NOT** constitute a **formal commitment for funding**. The final decision on your project will only be taken at a later stage, since it depends on the finalisation of grant preparation and further checks which we still need to make *(for instance, financial capacity, non-exclusion, etc.)*.

For calls with a limited number of proposals to be funded: If a call can fund only a limited number of proposals, grant preparation may be moreover be stopped if other proposals move up in the ranking after a successful evaluation review and re-evaluation procedure.

Commission européenne/Europese Commissie, 1049 Bruxelles/Brussel, BELGIQUE/BELGIË - Tel. +32 22991111

I would be grateful if you could inform the other members of your consortium (if any) of this letter.

For any questions, please contact us via your Funding & Tenders Portal account.

Yours faithfully,

Irene NORSTEDT
Acting Director

Enclosure: Evaluation summary report (ESR)

Commission européenne/Europese Commissie, 1049 Bruxelles/Brussel, BELGIQUE/BELGIË - Tel. +32 22991111
