# Supplementary material for: SPIOMET4HEALTH—efficacy, tolerability and safety of lifestyle intervention plus a fixed dose combination of spironolactone, pioglitazone and metformin (SPIOMET) for adolescent girls and young women with polycystic ovary syndrome: study protocol for a multicentre, randomised, double-blind, placebo-controlled, four-arm, parallel-group, phase II clinical trial
Source: Trials. 2023 Sep 15;24:589. doi: 10.1186/s13063-023-07593-6 (PMC10503102; doi:10.1186/s13063-023-07593-6)
Supplement: Supplementary file 2 — Additional file 2. Ethical approval documents. [file 13063_2023_7593_MOESM2_ESM.pdf]

Fundació Privada per a la Recerca i la Docència Sant Joan de Déu NIF: G-62978689

DICTAMEN DEL COMITÉ DE ÉTICA DE LA INVESTIGACIÓN CON MEDICAMENTOS

Dra. Neus Riba Garcia
Secretaria Técnica del CEIm Fundació Sant Joan de Déu

HACE CONSTAR:

Que el CEIm Fundació Sant Joan de Déu en su reunión del día **13/01/2022**, acta **01/2022**, ha evaluado la propuesta del promotor referida al estudio:

Código protocol: SPIOMET4HEALTH
Código EUDRACT: 2021-003177-58
Promotor: Fundació Privada per a la Recerca i la Docència Sant Joan de Déu - FSJD
Título: *"A Phase II, randomised, multi-centric, multi-national clinical trial to evaluate the efficacy, tolerability, and safety of a fixed dose combination of Spironolactone, Pioglitazone & Metformin (SPIOMET) for adolescent girls and young adult women (AYAs) with polycystic ovary syndrome (PCOS) / Ensayo clínico de fase II, aleatorizado, multicéntrico y multinacional para evaluar la eficacia, la tolerabilidad y la seguridad de una combinación de dosis fijas de espironolactona, pioglitazona y metformina (SPIOMET) para adolescentes y mujeres adultas jóvenes con síndrome de ovario poliquístico (SOP)"*
Investigador Principal: Lourdes Ibañez Toda
Centro: Hospital Sant Joan de Déu - Esplugues HSJD.1 (FSJD)
Código CEIm: AC-02-22

Que se emitieron aclaraciones tras la evaluación inicial que requerían de una nueva evaluación en reunión plenaria.

Que el CEIm Fundació Sant Joan de Déu en su reunión del día **24/03/2022**, acta **07/2022**, evaluó la respuesta recibida a las aclaraciones.

Que este Comité ha realizado la evaluación de la parte I de la solicitud de autorización del ensayo, ha valorado las respuestas del promotor a las aclaraciones solicitadas (si las hubiera) y ha transmitido a la Agencia Española de medicamentos su opinión final sobre la parte I.

Que este Comité ha realizado la evaluación de la parte II de la solicitud de autorización del ensayo, de acuerdo con lo previsto en el Real Decreto 1090/2015 y en el art 7 del reglamento (UE) 536/2014 y considera que:

Edifici Insitut Borja de Bioètica - c. Santa Rosa, 6, 1ª planta, 08950 Esplugues de Llobregat / Barcelona
Tel. Directe CEIm: +34 93 600 97 32 - Fax +34 93 600 97 71 e-mail: ceic@fsjd.org web: www.sjdrecerca.org

- El procedimiento para obtener el consentimiento informado (incluyendo las hojas de información al sujeto de ensayo y consentimientos informados mencionados en el encabezamiento), y el plan de reclutamiento de sujetos previsto son adecuados y cumplen con los requisitos para la obtención del consentimiento informado previstos en el capítulo II del Real Decreto 1090/2015.
- Las compensaciones previstas a los participantes son adecuadas, así como las previsiones de indemnización por daños y perjuicios que pueda sufrir el participante.
- El procedimiento previsto para el manejo de datos personales es adecuado.
- El uso futuro de las muestras biológicas obtenidas durante el ensayo se adecua a lo previsto en el Real Decreto 1716/2011.
- Para la realización del ensayo se consideran adecuados los centros e investigadores previstos en el anexo II a este dictamen, teniendo en cuenta las declaraciones de idoneidad emitidas por el promotor y por los responsables de las instituciones correspondientes.

Que este Comité decidió emitir **DICTAMEN FAVORABLE** en la reunión celebrada el día **24/03/2022**, acta **07/2022**.

Documentos con versiones:

Protocolo	Versión 3.0 - 16/03/2022
Manual del Investigador	Versión 4.0 - septiembre 2021
Hoja de información para la paciente y formulario de consentimiento informado para padres y tutores	Versión 2.0 - 24-Jan-22
Hoja de información y consentimiento informado al paciente menor de edad - (12-17 años)	Versión 3.0 - 21/03/2022
Plan de la campaña de reclutamiento	Versión 3.0 - 28Feb2022
Certificado de Seguro	Versión 04/02/2022
Memoria Económica	Versión diciembre 2021

Que en dicha reunión se cumplieron los requisitos establecidos en la legislación vigente –Real Decreto 1090/2015 – para que la decisión del citado CEIm sea válida.

Que el CEIm Fundació Sant Joan de Déu, tanto en su composición como en sus procedimientos, cumple con las normas de BPC (CPMP/ICH/135/95) y con la legislación vigente que regula su funcionamiento, y que la composición del CEIm Fundació Sant Joan de Déu es la indicada en el anexo I, teniendo en cuenta que en el caso de que algún miembro participe en el ensayo o declare

Fundació Privada per a la Recerca i la Docència Sant Joan de Déu NIF: G-62978689

Edifici Insitut Borja de Bioètica - c. Santa Rosa, 6, 1ª planta, 08950 Esplugues de Llobregat / Barcelona
Tel. Directe CEIm: +34 93 600 97 32 - Fax +34 93 600 97 71 e-mail: ceic@fsjd.org web: www.sjdrecerca.org

algún conflicto de interés no habrá participado en la evaluación ni en el dictamen de la solicitud de autorización del ensayo clínico.

Lo que firmo en Esplugues de Llobregat (Barcelona), a

Fdo:

Neus Riba Garcia
Firmado digitalmente por Neus Riba Garcia
Fecha: 2022.03.25 13:58:49 +01'00'

Dra. Neus Riba Garcia
Secretaria Técnica del CEIm Fundació Sant Joan de Déu

Fundació Privada per a la Recerca i la Docència Sant Joan de Déu NIF: G-62978689

Edifici Insitut Borja de Bioètica - c. Santa Rosa, 6, 1ª planta, 08950 Esplugues de Llobregat / Barcelona
Tel. Directe CEIm: +34 93 600 97 32 - Fax +34 93 600 97 71 e-mail: ceic@fsjd.org web: www.sjdrecerca.org

Fundació Privada per a la Recerca i la Docència Sant Joan de Déu NIF: G-62978689

Anexo I Dictamen inicial sobre la Parte II
COMPOSICION DEL CEIm

Presidente:
- Jesús Pineda Sánchez - Medicina – Pediatría

Vicepresidente:
- Bernabé Robles Del Olmo - Medicina – Neurología

Secretaria:
- Neus Riba Garcia - Farmacología Clínica

Vocales:
- Fernando Aguiló Martínez - Medicina Tropical
- Clara Chamorro Pérez - Jurista
- Ángel del Campo Escota - Representante de las asociaciones de pacientes
- Beatriz Del Pino Gaya - Farmacia hospitalaria
- Rosa María Dueñas Herrero - Medicina – Psiquiatría
- Pau Ferrer Salvans - Farmacología Clínica
- Joan Vinent Genestar - Farmacia hospitalaria
- María Eugènia Rey Abella - Farmacia AP
- Laura Martínez Rodríguez - Enfermería
- Marisa Serra Alacid - Medicina – Unidad Atención al Usuario
- Ana Martín Ancel - Medicina – Neonatología
- Eduard Puig Vaquero - Jurista – Delegado protección de datos
- Carlota Romans Ruiz - Farmacología Clínica
- Antoni Noguera Julián - Medicina – Pediatría
- Edurne Mazarico Gallego - Medicina – Ginecología y Obstetricia
- Esther Via Virgili - Medicina – Psiquiatría
- Oriol Martín Solé - Medicina – Cirugía y representante del CR
- Maite Gorostegui Obanos - Medicina – Oncología

Lo que firmo en Esplugues de Llobregat (Barcelona), a

Fdo: Neus Riba Garcia
Firmado digitalmente por Neus Riba Garcia
Fecha: 2022.03.25 13:58:28 +01'00'

Dra. Neus Riba Garcia
Secretaria Técnica del CEIm Fundació Sant Joan de Déu

Edifici Insitut Borja de Bioètica - c. Santa Rosa, 6, 1ª planta, 08950 Esplugues de Llobregat / Barcelona
Tel. Directe CEIm: +34 93 600 97 32 - Fax +34 93 600 97 71 e-mail: ceic@fsjd.org web: www.sjdrecerca.org

Fundació Privada per a la Recerca i la Docència Sant Joan de Déu NIF: G-62978689

Anexo II Dictamen Inicial sobre la Parte II
CENTROS E INVESTIGADORES PRINCIPALES PARTICIPANTES EN ESPAÑA

Código protocol: SPIOMET4HEALTH
Código EUDRACT: 2021-003177-58
Promotor: Fundació Privada per a la Recerca i la Docència Sant Joan de Déu - FSJD FSJD
Título: *"A Phase II, randomised, multi-centric, multi-national clinical trial to evaluate the efficacy, tolerability, and safety of a fixed dose combination of Spironolactone, Pioglitazone & Metformin (SPIOMET) for adolescent girls and young adult women (AYAs) with polycystic ovary syndrome (PCOS) / Ensayo clínico de fase II, aleatorizado, multicéntrico y multinacional para evaluar la eficacia, la tolerabilidad y la seguridad de una combinación de dosis fijas de espironolactona, pioglitazona y metformina (SPIOMET) para adolescentes y mujeres adultas jóvenes con síndrome de ovario poliquístico (SOP)"*
Investigador Principal: Lourdes Ibañez Toda
Centro: Hospital Sant Joan de Déu - Esplugues HSJD.1 (FSJD)
Código CEIm: AC-02-22

- Lourdes Ibañez Toda. HOSPITAL SANT JOAN DE DEU.
- Abel López Bermejo. Hospital Universitari de Girona Dr. Trueta.

Lo que firmo en Esplugues de Llobregat (Barcelona), a

Fdo:

Neus Riba Garcia
Firmado digitalmente por Neus Riba Garcia
Fecha: 2022.03.25 13:58:11 +01'00'

Dra. Neus Riba Garcia
Secretaria Técnica del CEIm Fundació Sant Joan de Déu

Edifici Insitut Borja de Bioètica - c. Santa Rosa, 6, 1ª planta, 08950 Esplugues de Llobregat / Barcelona
Tel. Directe CEIm: +34 93 600 97 32 - Fax +34 93 600 97 71 e-mail: ceic@fsjd.org web: www.sjdrecerca.org

CERTIFICATION OF TRANSLATION

Quote number: 2204-03699

Source document: SPIOMET4HEALTH_Spain_Approval_EC_25Mar2022.pdf

Full Title of Documents: SPIOMET4HEALTH_Spain_Approval_EC_25Mar2022_EN_translated 14Apr2022.docx

Footer: SPIOMET4HEALTH_Spain_Approval_EC_25Mar2022, translated into English on 14Apr2022

Original Source Language of Document: Spanish
Language Document is to be Translated into: English

Declaration of Translator:

I, Jan Hirš on behalf of Skrivanek s. r. o., hereby certify that the translation into English agrees with the text of the original document in Spanish language, that there were no changes/corrections made to the original document neither the translation, and that it was translated by a certified translator fluent in both above mentioned languages.

I, Jan Hirš on behalf of Skrivanek s. r. o., also confirm that the above mentioned service has been rendered and that the translated document is to the best of my knowledge a true and faithful translation of the original document as provided by the client

With Best regards,

OPINION OF THE ETHICS COMMITTEE ON DRUGS RESEARCH

Dr Neus Riba Garcia
CEIm Fundació Sant Joan de Déu Technical Secretary

CERTIFIES:

That the CEIm Fundació Sant Joan de Déu at its meeting on **13/01/2022**, minutes **01/2022**, assessed the proposal by the developer with reference to the study:

Protocol code: SPIOMET4HEALTH
EUDRACT code: 2021-003177-58
Developer: Fundació Privada per a la Recerca i la Docència Sant Joan de Déu - FSJD
Job title: *"A Phase II, randomised, multi-centric, multi-national clinical trial to evaluate the efficacy, tolerability, and safety of a fixed dose combination of Spironolactone, Pioglitazone & Metformin (SPIOMET) for adolescent girls and young adult women (AYAs) with polycystic ovary syndrome (PCOS) / Ensayo clínico de fase II, aleatorizado, multicéntrico y multinacional para evaluar la eficacia, la tolerabilidad y la seguridad de una combinación de dosis fijas de espironolactona, pioglitazona y metformina (SPIOMET) para adolescentes y mujeres adultas jóvenes con síndrome de ovario poliquístico (SOP)"*
Main researcher: Lourdes Ibañez Toda
Centre: Hospital Sant Joan de Déu - Esplugues HSJD.1 (FSJD)
CEIm code: AC-02-22

That clarifications were issued after the initial assessment that required further assessment at a plenary meeting.

That the CEIm Fundació Sant Joan de Déu at its meeting on **24/03/2022**, minutes **07/2022**, assessed the response received to the clarifications.

That this Committee undertook the assessment of Part I of the request for authorisation of the trial, considered the responses from the developer regarding the clarifications requested (where applicable) and informed the Spanish Agency of Medicines and Medical Devices of its final opinion on Part I.

That this Committee undertook the assessment of Part II of the request for authorisation of the trial, in accordance with the provisions of Royal Decree 1090/2015 and art. 7 of (EU) regulation

Edifici Insitut Borja de Bioètica - c. Santa Rosa, 6, 1ª planta, 08950 Esplugues de Llobregat / Barcelona
Direct Tel. CEIm: +34 93 600 97 32 - Fax +34 93 600 97 71 e-mail: ceic@fsjd.org web: www.sjdrecerca.org

536/2014 and considers that:

- The procedure to obtain informed consent (including the information sheets for the subject of the trial and informed consent mentioned in the heading), and the subject recruitment plan anticipated are adequate and meet the requirements for obtaining informed consent as established in chapter II of Royal Decree 1090/2015.
- The payment anticipated for the participants is adequate, as is the anticipated compensation for damages and loss that may be suffered by the participant.
- The procedure anticipated for processing personal data is adequate.
- The future use of the biological samples obtained during the trial adapts to the provisions of Royal Decree 1716/2011.
- For the performance of the trial, the centres and researchers listed in Annex II of this opinion are considered adequate, considering the declarations of suitability issued by the developer and the managers of the corresponding institutions.

That this Committee decided to issue a **FAVOURABLE OPINION** at its meeting on **24/03/2022**, minutes **07/2022**.

Documents with versions:

Protocol	Version 3.0 - 16/03/2022
Investigator's Brochure	Version 4.0 - September 2021
Patient Information Sheet and Informed Consent Form for parents and guardians	Version 2.0 - 24-Jan-22
Information Sheet and Informed Consent Form for minors - (12-17 years of age)	Version 3.0 - 21/03/2022
Recruitment campaign plan	Version 3.0 - 28Feb2022
Certificate of Insurance	Version 04/02/2022
Economic Report	Version December 2021

That at said meeting the requirements established in current legislation (Royal Decree 1090/2015) for the decision by the CEIm to be valid were met.

That the CEIm Fundació Sant Joan de Déu, in both its composition and its procedures, complies with the GCP standards (CPMP/ICH/135/95) and with current legislation that regulates its operation, and that the composition of the CEIm Fundació Sant Joan de Déu is that stated in Annex I, taking into consideration that in the case that any member participates in the trial or

declares any conflict of interest, they will not have participated in the assessment or in the opinion for the request for authorisation for clinical trial.

Signed in Esplugues de Llobregat (Barcelona), on

Signed:

Neus Riba

Digitally signed by Neus Riba Garcia
Date: 2022.03.25 13:58:49 +01'00'

Dr Neus Riba Garcia
CEIm Fundació Sant Joan de Déu Technical Secretary

Edifici Insitut Borja de Bioètica - c. Santa Rosa, 6, 1ª planta, 08950 Esplugues de Llobregat / Barcelona
Direct Tel. CEIm: +34 93 600 97 32 - Fax +34 93 600 97 71 e-mail: ceic@fsjd.org web: www.sjdrecerca.org

Annex I Initial opinion on Part II
COMPOSITION OF THE CEIm

Chairman:
- Jesús Pineda Sánchez - Medicine – Paediatrics

Deputy Chairman:
- Bernabé Robles Del Olmo - Medicine – Neurology

Secretary:
- Neus Riba Garcia - Clinical Pharmacology

Members:
- Fernando Aguiló Martínez - Tropical Medicine
- Clara Chamorro Pérez - Lawyer
- Ángel del Campo Escota - Representative of the patient associations
- Beatriz Del Pino Gaya - Hospital pharmacy
- Rosa María Dueñas Herrero - Medicine – Psychiatry
- Pau Ferrer Salvans - Clinical Pharmacology
- Joan Vinent Genestar - Hospital Pharmacy
- María Eugènia Rey Abella - GP Pharmacy
- Laura Martínez Rodríguez - Nursing
- Marisa Serra Alacid - Medicine – User Support Service
- Ana Martín Ancel - Medicine – Neonatology
- Eduard Puig Vaquero - Lawyer – Data protection officer
- Carlota Romans Ruiz - Clinical Pharmacology
- Antoni Noguera Julián - Medicine – Paediatrics
- Edurne Mazarico Gallego - Medicine – Gynaecology and Obstetrics
- Esther Via Virgili - Medicine – Psychiatry
- Oriol Martín Solé - Medicine – Surgery and CR representative
- Maite Gorostegui Obanos - Medicine – Oncology

Signed in Esplugues de Llobregat (Barcelona), on

Signed:

Neus Riba

Digitally signed by Neus Riba Garcia
Date: 2022.03.25 13:58:49 +01'00'

Dr Neus Riba Garcia
CEIm Fundació Sant Joan de Déu Technical Secretary

Edifici Insitut Borja de Bioètica - c. Santa Rosa, 6, 1ª planta, 08950 Esplugues de Llobregat / Barcelona
Direct Tel. CEIm: +34 93 600 97 32 - Fax +34 93 600 97 71 e-mail: ceic@fsjd.org web: www.sjdrecerca.org

Annex II Initial Opinion on Part II
MAIN CENTRES AND RESEARCHERS PARTICIPATING IN SPAIN

Protocol code: SPIOMET4HEALTH
EUDRACT code: 2021-003177-58
Developer: Fundació Privada per a la Recerca i la Docència Sant Joan de Déu - FSJD
Job title: *"A Phase II, randomised, multi-centric, multi-national clinical trial to evaluate the efficacy, tolerability, and safety of a fixed dose combination of Spironolactone, Pioglitazone & Metformin (SPIOMET) for adolescent girls and young adult women (AYAs) with polycystic ovary syndrome (PCOS) / Ensayo clínico de fase II, aleatorizado, multicéntrico y multinacional para evaluar la eficacia, la tolerabilidad y la seguridad de una combinación de dosis fijas de espironolactona, pioglitazona y metformina (SPIOMET) para adolescentes y mujeres adultas jóvenes con síndrome de ovario poliquístico (SOP)"*
Main researcher: Lourdes Ibañez Toda
Centre: Hospital Sant Joan de Déu - Esplugues HSJD.1 (FSJD)
CEIm code: AC-02-22

- Lourdes Ibañez Toda. HOSPITAL SANT JOAN DE DEU.
- Abel López Bermejo. Hospital Universitari de Girona Dr. Trueta.

Signed in Esplugues de Llobregat (Barcelona), on

Signed:

Neus Riba

Digitally signed by Neus Riba Garcia
Date: 2022.03.25 13:58:49 +01'00'

Dr Neus Riba Garcia
CEIm Fundació Sant Joan de Déu Technical Secretary

Edifici Insitut Borja de Bioètica - c. Santa Rosa, 6, 1ª planta, 08950 Esplugues de Llobregat / Barcelona
Direct Tel. CEIm: +34 93 600 97 32 - Fax +34 93 600 97 71 e-mail: ceic@fsjd.org web: www.sjdrecerca.org

COMUNICAZIONE AL RICHIEDENTE, AGLI ALTRI COMITATI ETICI E AD AIFA DELLA DECISIONE DEL COMITATO ETICO RELATIVA AL PARERE UNICO

Il parere finale (favorevole o non favorevole) deve essere trasmesso entro trenta giorni dalla data di ricevimento della domanda nella forma prescritta (entro sessanta giorni in caso di sperimentazione monocentrica)

A. IDENTIFICAZIONE DELLA SPERIMENTAZIONE

A.1 Versione CTA valutata
1.4

A.1.1 Note
Appendice 5 V.1.4 firmata in data 16/05/2022

A.1.2 Numero EudraCT
2021-003177-58

A.2 Titolo completo della sperimentazione
Studio clinico di fase II, randomizzato, multicentrico e multinazionale per valutare l'efficacia, la tollerabilitÃ e la sicurezza di una combinazione a dose fissa di Spironolattone, Pioglitazone e Metformina (SPIOMET) per adolescenti e giovani adulte (AYA) con sindrome dell'ovaio policistico (PCOS)

A.3.1 Codice del protocollo
SPIOMET4HEALTH

A.3.2 Versione del protocollo
3.0

A.3.3 Data del protocollo
16/03/2022

B. IDENTIFICAZIONE DEL COMITATO ETICO (CE) ISTITUITO AI SENSI DEL D.M. 8 FEBBRAIO 2013

B.1 Denominazione del CE
COMITATO ETICO INDIPENDENTE DI AREA VASTA EMILIA CENTRO

B.2.1 Nome del Presidente
Elisabetta

B.2.2 Cognome del Presidente
Poluzzi

B.3 Indirizzo del CE
VIA ALBERTONI 15

B.4 Numero di telefono
0512141384

B.5 Numero di fax
0516361249

B.6 E-mail
cometico@aosp.bo.it

C. IDENTIFICAZIONE DELLO SPERIMENTATORE COORDINATORE (SE STUDIO MONOCENTRICO, DELLO SPERIMENTATORE PRINCIPALE)

C.1 Nome
Alessandra

C.2 Cognome
Gambineri

C.3 Centro clinico
AZIENDA OSPEDALIERO-UNIVERSITARIA DI BOLOGNA

C.4 Indirizzo del centro clinico
VIA GIUSEPPE MASSARENTI

C.5 Reparto
Dipartimento di Scienze Mediche e Chirurgiche

D. DOCUMENTAZIONE ESAMINATA

D.1 Data di ricezione della domanda nella forma prescritta
17/01/2022

D.2 Data di ricezione di informazioni integrative (ove applicabile)

D.3 Modulo di domanda (Appendice 5) [x]

D.4 Documentazione riportata nel modulo di domanda [x]

E. ELEMENTI VALUTATI (SELEZIONARE NA NEI CASI IN CUI L'INFORMAZIONE NON SIA APPLICABILE)

**E.1 Dati di qualità del medicinale sperimentale**				
**E.1.1 Le informazioni e i dati necessari a supportare la qualita' dell'IMP sono adeguati**				☒
**E.1.2 Il promotore ha documentato che i prodotti in sperimentazione saranno preparati gestiti e conservati nel rispetto delle Norme di Buona Fabbricazione (GMP) applicabili**				☒
**E.1.2.1 Eventuali elementi critici riscontrati**				
**E.2 Dati di farmacologia non clinica e tossicologia**				
**E.2.1 Esistono presupposti solidi e rilevanti che giustificano l'avvio dello studio**				☒
**E.2.1.1 Eventuali elementi critici riscontrati**				
**E.3 Dati clinici**				
**E.3.1 Esistono presupposti solidi e rilevanti che giustificano l'avvio dello studio (non applicabile per studi di fase I e II)**	NA	☒	Sì	☐
**E.3.2 Lo studio consentira' di acquisire maggiori informazioni sull'IMP di migliorare le procedure profilattiche diagnostiche e terapeutiche o la comprensione dell'eziologia e della patogenesi delle malattie**				☒
**E.3.2.1 Eventuali elementi critici riscontrati**				
**E.4 Protocollo**				
**E.4.1 Gli obiettivi sono coerenti con il razionale scientifico**				☒
**E.4.2 Il disegno dello studio e' pertinente e rilevante**				☒
**E.4.3 Sono stati esaminati i seguenti aspetti**				
**E.4.4 Mancanza del gruppo di controllo**	NA	☒	Sì	☐
**E.4.5 Disegno in aperto**	NA	☒	Sì	☐
**E.4.6 Assenza di randomizzazione**	NA	☒	Sì	☐
**E.4.7 Uso del placebo quale gruppo di controllo**	NA	☐	Sì	☒
**E.4.8 Disegno di equivalenza o di non inferiorita'**	NA	☒	Sì	☐
**E.4.9 Lo schema di trattamento con l'IMP risulta adeguato (via di somministrazione dosaggio e posologia durata della terapia)**				☒
**E.4.10 Il trattamento di controllo e lo schema di trattamento sono giustificati**	NA	☐	Sì	☒
**E.4.11 I criteri di inclusione/esclusione sono appropriati chiari e ben definiti**				☒
**E.4.12 Gli esami le visite e le procedure previste (specie se invasive) sono idonei a verificare gli effetti del trattamento**				☒
**E.4.13 La misura di esito primaria e' clinicamente rilevante o correlabile a una misura clinicamente rilevante**	NA	☐	Sì	☒
**E.4.14 I metodi per rilevare la misura di esito primaria risultano adeguati**	NA	☐	Sì	☒
**E.4.15 Il calendario previsto per la rilevazione dei parametri di efficacia e' appropriato**	NA	☐	Sì	☒
**E.4.16 I parametri selezionati per la valutazione della sicurezza sono congrui**				☒
**E.4.17 Il follow-up ha una durata sufficiente in relazione all'obiettivo dello studio**				☒
**E.4.18 La dimensione campionaria e' stata calcolata in funzione della misura di esito primaria dichiarata**	NA	☐	Sì	☒
**E.4.19 Il calcolo della dimensione campionaria e' corretto in relazione alla potenza prevista per lo studio**	NA	☐	Sì	☒
**E.4.20 Il piano statistico di analisi dei dati e' coerente rispetto agli obiettivi**				☒
**E.4.21 La differenza attesa tra i trattamenti confrontati e' significativa**	NA	☐	Sì	☒
**E.4.22 In caso di studio di equivalenza o di non inferiorita' la differenza considerata non rilevante e' sufficientemente ristretta ed accettabile**	NA	☒	Sì	☐
**E.4.23 Il protocollo e' conforme alle linee guida EMA in materia**	NA	☒	Sì	☐
**E.4.23.1 Se si al punto precedente specificarne i riferimenti:**				

**E.4.24 Eventuali elementi critici riscontrati**				
**E.5 Aspetti etici**				
**E.5.1 Il promotore ha documentato che la sperimentazione verrà condotta in conformità ai principi etici che traggono la loro origine dalla Dichiarazione di Helsinki, e che rispetta le GCP e le disposizioni normative applicabili**				[x]
**E.5.2 I rischi e gli inconvenienti prevedibili sono stati soppesati rispetto al vantaggio per il soggetto incluso nella sperimentazione e per altri pazienti attuali e futuri**				[x]
**E.5.3 Il Comitato Etico e' giunto alla conclusione che i benefici previsti dalla sperimentazione terapeutici e in materia di sanita' pubblica ne giustifichino i rischi**				[x]
**E.5.4 I diritti la sicurezza e il benessere dei soggetti dello studio hanno costituito le considerazioni piu' importanti e sono prevalsi sugli interessi della scienza e della societa'**				[x]
**E.5.5 La ricerca su persone che non sono in grado di dare il loro consenso informato e' giustificata**	NA	[x]	Sì	[ ]
**E.5.6 Sono attesi possibili benefici diretti per il soggetto**	NA	[ ]	Sì	[x]
**E.5.7 Sono attesi possibili benefici per la collettivita'**	NA	[ ]	Sì	[x]
**E.5.7.1 Eventuali elementi critici riscontrati**				
**E.6 Informazione dei soggetti e procedure per il consenso informato**				
**E.6.1 Le informazioni per il paziente sono complete e comprensibili**				[x]
**E.6.2 Le procedure previste dal protocollo sono indicate in modo esauriente**				[x]
**E.6.3 I disagi e i rischi cui il paziente potrebbe essere esposto sono ben descritti**				[x]
**E.6.4 Le modalita' di ottenimento del consenso sono ben esplicitate**				[x]
**E.6.5 Le modalita' di coinvolgimento di volontari sani sono adeguate**	NA	[x]	Sì	[ ]
**E.6.6 Le misure adottate per la salvaguardia della privacy del soggetto e la tutela dei dati personali sono appropriate in accordo alla normativa vigente**				[x]
**E.6.7 Le modalita' di informazione al medico curante sono corrette e complete**	NA	[ ]	Sì	[x]
**E.6.7.1 Eventuali elementi critici riscontrati**				
**E.7 Aspetti economici e informazioni relative a strutture e personale**				
**E.7.1 Sono stati adeguatamente valutati gli elementi della proposta di contratto tra il promotore e il centro clinico**	NA	[ ]	Sì	[x]
**E.7.2 La copertura assicurativa garantisce un'appropriata tutela dei partecipanti**				[x]
**E.7.3 Gli importi le modalita' di retribuzione o di compenso o di emolumenti di qualsiasi natura previsti dall'amministrazione di competenza per gli sperimentatori sono conformi alle norme vigenti adeguati rispetto all'impegno richiesto e non tali da costituire elemento determinante per la conduzione della sperimentazione**	NA	[ ]	Sì	[x]
**E.7.4 E' stata considerata la congruita' dell'eventuale indennita' per i volontari sani che non deve essere tale da costituire elemento determinante per la partecipazione alla sperimentazione**	NA	[x]	Sì	[ ]
**E.7.5 E' stata esaminata l'idoneita' dello sperimentatore e dei suoi collaboratori**				[x]
**E.7.6 La struttura sanitaria dove si svolgera' lo studio e' appropriata**				[x]
**E.7.7 E' stato verificato che il promotore dichiara di garantire una corretta e rapida diffusione delle informazioni acquisite attraverso lo studio**				[x]
**E.7.7.1 Eventuali elementi critici riscontrati**				

F. DECISIONE DEL COMITATO ETICO

	NA	Sì	No
**F.1 Parere unico favorevole**		[x]	[ ]
**F.2 In caso di richiesta di parere su una sperimentazione non commerciale il CE ha accertato la sussistenza dei requisiti della normativa vigente**	[ ]	[x]	[ ]
**F.3 Parere unico non favorevole**		[ ]	[ ]
**F.4 Sperimentazione da condurre presso**			
**F.4.1 Stessa struttura**		[x]	[ ]
**F.4.2 Altra struttura**		[ ]	[x]

F.5 Numero di pazienti previsto nel centro
54

F.6 Contributo lordo previsto dal promotore (per soggetto completato ove applicabile)
n.a.

G. ASPETTI PARTICOLARI DELLO STUDIO CONSIDERATI NEL RILASCIO DEL PARERE UNICO FAVOREVOLE

G.1 Aspetti particolari dello studio considerati nel rilascio del parere unico favorevole

Acquisita la documentazione e i chiarimenti richiesti, il Comitato Etico, nella seduta telema-tica del giorno 18/05/2022, esprime allâunanimitÃ parere unico favorevole alla conduzione dello studio. Resta inteso che, in accordo con la natura no profit dello studio, non Ã¨ consentito lâutilizzo dei dati e dei risultati dello studio a scopo commerciale e/o di lucro. In caso contrario, qualora si esitasse in scoperte scientifiche brevettabili o comunque sfruttabili commercialmente da qualsiasi soggetto di natura profit, occorrerÃ riqualificare lo studio da no profit a profit con il conseguente pagamento della tariffa prevista per il Comitato Etico e dei costi diretti e indiretti dello studio ai sensi del D. Lgs. 52/2019, previa debita informazione al Comitato per il rilascio del parere. Si precisa infine che: â¢ lo studio potrÃ essere avviato solo dopo aver ricevuto il nulla osta da parte della Direzione Gene-rale della struttura sanitaria di riferimento ai sensi dellâart. 7 della L.R. n. 9/2017; â¢ ai fini del monitoraggio dellâandamento dello studio in oggetto, lo Sperimentatore Responsabi-le dovrÃ comunicare al Comitato Etico le seguenti informazioni relativamente a questo singolo centro sperimentale: data di inizio arruolamento, data di fine arruolamento e data di conclusio-ne dello studio. In ogni caso, a partire dallâanno di approvazione dello studio e fino alla sua conclusione, almeno una volta allâanno e comunque entro e non oltre il 31 dicembre, dovrÃ essere fornito un rapporto annuale sullo stato di avanzamento dello studio. Per le suddette co-municazioni Ã¨ possibile utilizzare il modulo disponibile sul sito web del Comitato Etico (https://www.aosp.bo.it/files/modulo_monitoraggio_ceavec_v3_5-7-19_def.doc). Il modulo compilato, firmato e datato, andrÃ inviato esclusivamente in formato elettronico allâindirizzo e-mail dedicato monitoraggiostudi@aosp.bo.it indicando nellâoggetto il nome dello Sperimenta-tore Responsabile locale ed il codice assegnato dal Comitato allo studio. Si precisa, infine, che il Comitato Etico Ã¨ costituito in conformitÃ al DM 08/02/2013 âCriteri per la composizione e il funzionamento dei comitati eticiâ. Si allega elenco dei membri presenti alla valutazione. La firma del parere sull'OsSC Ã¨ delegata al Dr. Giacomo Chiabrando, referente OsSC per il CE-AVEC, come da lettera di delega del Presidente datata 23/02/2022 disponibile presso la sede legale del CE-AVEC. Elenco documentazione esaminata nella seduta del 17/03/2022: â¢ Lettera di trasmissione della Optimapharm firmata il 17/01/2022 â¢ Appendice 5, v. 1.0 firmata digitalmente il 13/01/2022 â¢ Protocollo di Studio, v.2.0 del 04/12/2021 â¢ Sinossi del Protocollo in italiano, tradotta il 15/12/2021 v. 2.0 del 04/12/2021 â¢ Investigatorâs Brochure (IB) v.4.0 del Settembre 2021 â¢ Informativa e consenso informato per i genitori/rappresentante legale, v. 1.0 del 11/11/2021 â¢ Informativa e consenso informato per pazienti adulte, v. 1.0 del 11/11/2021 â¢ Informativa e assenso per pazienti adolescenti, v. 1.0 del 11/11/2021 â¢ Informativa e consenso al trattamento dati personali per genitori/rappresentante legale, v1.0 del 16/12/2021 â¢ Informativa e consenso al trattamento dati personali per pazienti adulte, v. 1.0 del 16/12/2021 â¢ Scheda partecipazione paziente v. 1.1 del 27.07.2021 â¢ Modulo centro specifico firmato il 15/02/2022 â¢ Dichiarazione collaborazione LUM del 12/01/2022 â¢ Lettera disponibilitÃ Radiologia Golfieri SPIOMET4HEALTH â¢ Lettera disponibilitÃ Radiologia Lovato SPIOMET4HEALTH â¢ Grant Agreement 899671 del 28/01/2021 â¢ Lettera di delega del 01/10/2021 â¢ Conferma numero EudraCT del 01/06/2021 â¢ Scientific Advice EMA: EMA/CHMP/SAWP/282878/2017 del 18/05/2017 â¢ Decisione PIP n. P/0150/2021 del 16/04/2021 â¢ Investigational Medicinal Product Dossier (IMPD) v.4.0 del Settembre 2021 â¢ Autorizzazione alla Produzione - LABORATORIO REIG JOFRÃ, S.A., Barcelona, Spain-MIA 0391-26/06/2021 â¢ Certificato GMP - LABORATORIO REIG JOFRÃ, S.A., Barcelona, Spain-NCF/2120/001/CAT del 31/03/2021 â¢ Contenuto dellâetichetta per il medicinale sperimentale v. 2.0 del 24/11/2021 Polizza assicurativa del 05/11/2021 â¢ Certificato di assicurazione del 29/11/2021 â¢ Elenco riepilogativo delle variabili della eCRF, v. 1.0 del 04/10/2021 â¢ Modulo notifica SAE, v. 2.0 del 02/11/2021 â¢ Screening dei disturbi alimentari (questionario SCOFF + BEDS-7), v. 2.0 del 30.09.2021 â¢ Opuscolo âIntervento sullo stile di vitaâ, v. 2.0 del 05/11/2021 â¢ Storyboard, v. 1.0 del 12/08/2021 â¢ Descrizione del diario del paziente, v. 1.0 del 29/09/2021 â¢ Diario del paziente e accettabilitÃ delle compresse, v. 1.0 del 29/09/2021 â¢ Sondaggio per i partecipanti allo studio, v 1.0 del 28/07/2021 â¢ Opuscolo (raccolta campione salivare) , v. 1.0 del 25/08/2021 â¢ Questionario di valutazione LIP, v 1.0 del 08/08/2021 â¢ Volantino attivitÃ di coinvolgimento del paziente, v. 1.0 del 01/07/2021 â¢ Questionario EDE-Q, v. 6.0 del 2008 â¢ Lettera per il pediatra/MMG, v. 2.0 del 14/12/2021 â¢ Piano della campagna di reclutamento, v. 1.0 del 27/11/2021 â¢ Pagina web, v. 2.0 del 08/10/2021 â¢ Procedura operativa standard su screening per i disturbi alimentari, v. 1.0 del 24/08/2021 â¢ Manuale - Programma di intervento sullo stile di vita per adolescenti/giovani donne con PCOS, v. 1.1 del 12/08/2021 â¢ Scheda documentale - Vivere in salute con PCOS, v. 1.0 del 03/08/2021 â¢ Elenco dei centri partecipanti, v. 1.0 del 25/10/2021 â¢ CV Sperimentatore principale, Prof.ssa Alessandra Gambineri del 04/08/2021 â¢ Certificato GCP

G.1 Aspetti particolari dello studio considerati nel rilascio del parere unico favorevole

Sperimentatore principale del 27/07/2016 â¢ Dichiarazione assenza conflitto di interesse del 11/10/2021 â¢ Checklist SPIRIT del 05/01/2022 â¢ Dichiarazione sulla natura no profit dello studio del 13/01/2022 â¢ Lettera di risposta alle richieste di validazione del 03/02/2022 â¢ Bozza convenzione economica in lingua inglese del 05/07/2021 â¢ Grant Agreement 899671, versione non firmata digitalmente del 28/01/2021 â¢ Autodichiarazione sulla natura no-profit del Promotore del 13/09/2021 â¢ Certificato dellâAgenzia Statale di Amministrazione Tributaria spagnola del 17/05/2021 â¢ Identificazione fiscale del Promotore, FSJD â¢ Questionario per auto compilazione del 01/06/2021 â¢ Testo per la somministrazione del colloquio del 03/12/2018 â¢ Certificato di traduzione del questionario del 01/06/2021 â¢ Certificato di traduzione del testo per la somministrazione colloquio del 03/12/2018 â¢ Questionario QualitÃ della Vita PCOSQ Elenco documentazione esaminata nella seduta del 18/05/2022: â¢ Lettera di risposta ai chiarimenti da parte di Optimapharm d.o.o. datata 11/05/2022 â¢ Appendice 5 V.1.4 firmata in data 16/05/2022 â¢ Protocollo di Studio V.3.0 del 16/03/2022 â¢ Investigational Medicinal Product Dossier (IMPD): o IMPD SPIOMET V.5.0 â Marzo 2022 o IMPD Pioglitazone V.2.0 â Marzo 2022 o IMPD SPIO V.2.0 â Marzo 2022 o IMPD Placebo V.2.0 â Marzo 2022 â¢ Informative e moduli di consenso informato: o Informativa e consenso informato per i genitori/rappresentante legale V.2.0 del 10/05/2022 o Informativa e consenso informato per pazienti adulte V.2.0 del 10/05/2022 o Informativa e assenso per pazienti adolescenti V.2.0 del 10/05/2022 o Informativa sullâutilizzo futuro dei campioni per i genitori V.1.0 del 09/05/2022 o Informativa sullâutilizzo futuro dei campioni per partecipanti adulte V.1.0 del 09/05/2022 â¢ Altro materiale da fornire al paziente: o Scheda partecipazione paziente V1.0 del 27/07/2021 o Opuscolo âIntervento sullo stile di vitaâ V.2.0 del 05/11/2021 o Descrizione del diario del paziente V.1.0 del 29/09/2021 o Diario del paziente e accettabilitÃ delle compresse V.1.0 del 29/09/2021 o Sondaggio per i partecipanti allo studio V1.0 del 28/07/2021 o Questionario di valutazione LIP V.2.0 del 14/01/2022 o Volantino attivitÃ di coinvolgimento del paziente V.1.0 del 01/07/2021 o Lettera per il pediatra/MMG V.2.0 del 14/12/2021 â¢ Disposizioni per il reclutamento dei soggetti: o Piano della campagna di reclutamento V.1.0 del 27/11/2021 o Pagina web V.2.0 del 08/10/2021 â¢ Lettera di trasmissione risposta alle obiezioni motivate di AIFA datata 23/03/2022 â¢ Risposta alle obiezioni motivate di AIFA datata 23/03/2022 â¢ Dichiarazione sui composti N-nitroso del Pioglitazone datata 20/10/2021 â¢ Rapporto sullo studio di valutazione del rischio per NDMA (nitrosodimetilammina) e NDEA (nitrosodietilammina) nella Metformina - 09/06/2020 â¢ Certificato di idoneitÃ â Spironolattone - 29/05/2017 â¢ Dichiarazione di conformitÃ alle linee guida ICH Q3D per le impuritÃ elementari â Spironolattone - 02/11/2021 â¢ Dichiarazione di origine â LIGAMED MF-2-V/K (stearato di magnesio) - 15/01/2018 â¢ Dichiarazione sui risultati della verifica dei metodi analitici - 01/03/2022 â¢ Studio di verifica per la determinazione di sostanze correlate della Metformina in SPIOMET - 14/12/2021 â¢ Studio di verifica del dosaggio e dellâuniformitÃ del contenuto di Metformina, Pioglitazone e Spironolattone nelle compresse di SPIOMET, SPIO e PIO - 30/11/2021 â¢ Studio di verifica per la determinazione di sostanze correlate di Pioglitazone e Spironolactone in compresse di SPIOMET, SPIO e PIO - 25/01/2022 â¢ Studio di verifica del test di dissoluzione di Metformina, Pioglitazone e Spironolattone in compresse SPIOMET - 21/02/2022 â¢ Studio di verifica del test di dissoluzione di Pioglitazone e Spironolactone in compresse SPIOMET - 21/02/2022 â¢ Analisi di stabilitÃ del lotto SPIOMET H1756 utilizzato nello studio clinico Fase I KPI-002-CL-002 - 15/04/2021 â¢ Studio di stabilitÃ di lotti di farmaco Spiomet frasco 35 comp, Spio (Spiomet) frasco 35 comp, PIO(SPIOMET) frasco 35 comp e Placebo Spiomet frasco 35 comp batch 22101E - 16/03/2022 â¢ Autorizzazione AIFA rilasciata in data 28/03/2022

H. MOTIVAZIONI DEL PARERE UNICO NON FAVOREVOLE

H.1 Protocollo

H.1.1 Rilevanza della sperimentazione ☐

H.1.2 Criteri di inclusione ed esclusione ☐

H.1.3 Gruppo di controllo ☐

H.2 Informazione dei soggetti e procedure per il consenso informato

H.2.1 Procedure per il reclutamento ☐

H.2.2 Foglio informativo modulo per il consenso informato e procedure ☐

H.2.3 Protezione dei dati personali e confidenzialita' ☐

H.3 Aspetti etici

H.3.1 Valutazione dei benefici e dei rischi prevedibili ☐

H.3.2 Misure per minimizzare il dolore il disagio e la paura ☐

H.3.3 Inclusione di persone incapaci di dare validamente il proprio consenso informato e altre popolazioni vulnerabili ☐

H.3.4 Adesione alle norme di Buona Pratica Clinica ☐

H.4.1 Idoneita' dello sperimentatore e dei suoi collaboratori ☐

H.4.2 Adeguatezza della struttura sanitaria ☐

H.4.3 Contratto tra promotore e centro clinico ☐

H.4.4 Polizza assicurativa ☐

H.4.5 Indennita' per i partecipanti allo studio ☐

H.4.6 Indennita' per gli sperimentatori ☐

H.4.7 Adempimenti degli obblighi amministrativi ☐

H.5 Altro Sì ☐ No ☐

H.5.1 Se si al punto precedente specificare

I. DESCRIZIONE DELLE MOTIVAZIONI DEL PARERE UNICO NON FAVOREVOLE

I.1 Descrizione delle motivazioni del parere unico non favorevole

L. SEDUTA DEL COMITATO ETICO

L.1 Data della seduta
18/05/2022

L.2 Numero del registro dei pareri del CE
179/2022/Farm/AOUBo

L.3 Componenti del CE presenti e qualifiche
Elisabetta Poluzzi - Farmacologo

L.3.1 Sostituto permanente che ha partecipato alla seduta in vece del Direttore sanitario
n.a.

L.4 Consulenti esterni presenti e qualifiche (ove applicabile)
n.a.

L.5 Componenti del CE presenti che non hanno partecipato alla votazione (ove applicabile)
La Dott.ssa Pelusi si astiene dalla votazione per conflitto di interessi

M. FIRMA DEL PRESIDENTE DEL COMITATO ETICO

M.1 Il Comitato Etico ha espresso il parere unico:

M.1.1 Verificata la sussistenza del numero legale essendo presenti membri n. ____
33

M.1.1.1 su n. _____
42

M.1.2 Tenuto conto di eventuali osservazioni ricevute dai comitati etici collaboratori
n.a.

M.2 Data
27/05/2022

M.2 Nome

M.2.1 Cognome

M.3 Firma

M.4 Allega file

DOCUMENTAZIONE

Documentazione	
Nome File:	elenco presenti assenti CE AVEC 18.05.2022_2.pdf

CERTIFICATION OF TRANSLATION

Quote number: 2206-00442

Source document: 179.2022.Farm.AOUBo_signed.pdf

Full Title of Documents: 179.2022.Farm.AOUBo_signed_translated into English on 8June2022.docx

Footer: 179.2022.Farm.AOUBo_signed_translated into English on 8June2022

Original Source Language of Document: Italian
Language Document is to be Translated into: English

Declaration of Translator:

I, Jan Hirš on behalf of Skrivanek s. r. o., hereby certify that the translation into English agrees with the text of the original document in Italian language, that there were no changes/corrections made to the original document neither the translation, and that it was translated by a certified translator fluent in both above mentioned languages.

I, Jan Hirš on behalf of Skrivanek s. r. o., also confirm that the above mentioned service has been rendered and that the translated document is to the best of my knowledge a true and faithful translation of the original document as provided by the client

With Best regards,

COMMUNICATION TO THE APPLICANT, THE OTHER ETHICS COMMITTEES AND AIFA OF THE DECISION OF THE ETHICS COMMITTEE RELATING TO THE SINGLE OPINION

The final opinion (favourable or unfavourable) must be sent within thirty days from the date of receipt of the application in the prescribed form (within sixty days in the case of monocentric experimentation)

A. IDENTIFICATION OF THE TRIAL

A.1 Assessed CTA version
1.4

A.1.1 Note
Annex 5 V.1.4 signed on 16/05/2022

A.1.2 EudraCT Number
2021-003177-58

A.2 Complete title of the trial
Phase II, Randomised, Multicentre, Multinational Clinical Study to Evaluate Efficacy, Tolerability and Safety of a Fixed-Dose Combination of Spironolactone, Pioglitazone, and Metformin (SPIOMET) for Adolescents and Young Adults (AYA) with Polycystic Ovary Syndrome(PCOS)

A.3.1 Protocol Code
SPIOMET4HEALTH

A.3.2 Protocol Version
3.0

A.3.3 Protocol Date
16/03/2022

B. IDENTIFICATION OF THE ETHICS COMMITTEE (EC) ESTABLISHED PURSUANT TO D.M. 08/02/2013

B.1 Name of the EC
INDEPENDENT ETHICS COMMITTEE OF AREA VASTA EMILIA CENTRO

B.2.1 Forename of the President
Elisabetta

B.2.2 Surname of the President
Poluzzi

B.3 Address of the EC
VIA ALBERTONI 15

B.4 Telephone number
0512141384

B.5 Fax number
0516361249

B.6 Email
cometico@aosp.bo.it

C. IDENTIFICATION OF THE COORDINATING INVESTIGATOR (IF A MONOCENTRIC STUDY, OF THE PRINCIPAL INVESTIGATOR)

C.1 Forename
Alessandra

C.2 Surname
Gambineri

C.3 Clinical centre
AZIENDA OSPEDALIERO-UNIVERSITARIA DI BOLOGNA

C.4 Address of the Clinical Centre
VIA GIUSEPPE MASSARENTI

C.5 Section
Department of Medical and Surgical Sciences

D. DOCUMENTATION EXAMINED

D.1 Date of receipt of the application in the prescribed form
17/01/2022

D.2 Date of receipt of additional information (if applicable)

D.3 Application form (Annex 5) [x]

D.4 Documentation reported in the application form [x]

E. ELEMENTS EVALUATED (SELECT NA IN CASES WHERE THE INFORMATION IS NOT APPLICABLE

Item	NA	Yes
**E.1 Quality data of the experimental drug**		
**E.1.1 The information and data necessary to support the quality of the IMP are adequate**		☒
**E.1.2 The promoter has documented that the products under trial will be prepared, managed and stored in compliance with the applicable Good Manufacturing Standards (GMP)**		☒
**E.1.2.1 Any critical elements found**		
**E.2 Non-clinical pharmacology and toxicology data**		
**E.2.1 There are solid and relevant assumptions that justify the start of the study**		☒
**E.2.1.1 Any critical elements found**		
**E.3 Clinical data**		
**E.3.1 There are strong and relevant assumptions justifying the initiation of the study (not applicable for phase I and II studies)**	NA ☒	Yes ☐
**E.3.2 The study will make it possible to acquire more information on IMP to improve diagnostic and therapeutic prophylactic procedures or to understand the aetiology and pathogenesis of diseases.**		☒
**E.3.2.1 Any critical elements found**		
**E.4 Protocol**		
**E.4.1 The objectives are consistent with the scientific rationale**		☒
**E.4.2 The study design is pertinent and relevant**		☒
**E.4.3 The following aspects were examined**		
**E.4.4 Lack of control group**	NA ☒	Yes ☐
**E.4.5 Open design**	NA ☒	Yes ☐
**E.4.6 No randomisation**	NA ☒	Yes ☐
**E.4.7 Use of placebo as a control group**	NA ☐	Yes ☒
**E.4.8 Equivalence or non-inferiority design**	NA ☒	Yes ☐
**E.4.9 The treatment schedule with the IMP is adequate (route of administration, dosage and posology, duration of therapy)**		☒
**E.4.10 The control treatment and the treatment method are justified**	NA ☐	Yes ☒
**E.4.11 The inclusion/exclusion criteria are appropriate clear and well defined**		☒
**E.4.12 The examinations, visits and planned procedures (especially if invasive) are suitable for checking the effects of the treatment**		☒
**E.4.13 The primary outcome measure is clinically relevant or correlated with a clinically relevant measure**	NA ☐	Yes ☒
**E.4.14 The methods for taking the primary outcome measure are adequate**	NA ☐	Yes ☒
**E.4.15 The schedule foreseen for detecting the efficacy parameters is appropriate**	NA ☐	Yes ☒
**E.4.16 The parameters selected for the safety evaluation are congruous**		☒
**E.4.17 The follow-up is of sufficient duration in relation to the objective of the study**		☒
**E.4.18 The sample size was calculated on the basis of the primary outcome measure declared**	NA ☐	Yes ☒
**E.4.19 The calculation of the sample size is correct in relation to the power expected for the study**	NA ☐	Yes ☒
**E.4.20 The statistical data analysis plan is consistent with the objectives**		☒
**E.4.21 The expected difference between the treatments compared is significant**	NA ☐	Yes ☒
**E.4.22 In the case of an equivalence or non-inferiority study, the difference considered not relevant is sufficiently small and acceptable**	NA ☒	Yes ☐
**E.4.23 The protocol complies with the relevant EMA guidelines**	NA ☒	Yes ☐
**E.4.23.1 If yes is answered to the previous point, specify the references:**		

E.4.24 Any critical elements found

E.5 Ethical elements

E.5.1 The promoter has documented that the trial will be conducted in compliance with the ethical principles that originate from the Declaration of Helsinki, and which respects the GCP and applicable regulatory provisions x

E.5.2 The foreseeable risks and inconveniences have been weighed against the benefit for the subject included in the trial and for other current and future patients x

E.5.3 The Ethics Committee has come to the conclusion that the benefits envisaged by the therapeutic and public health experimentation justify the risks x

E.5.4 The rights to safety and well-being of the study subjects have constituted the most important considerations and prevailed over the interests of science and society x

E.5.5 Research on people who are unable to give their informed consent is justified NA x Yes

E.5.6 Possible direct benefits for the subject are expected NA Yes x

E.5.7 Possible benefits for the community are expected NA Yes x

E.5.7.1 Any critical elements found

E.6 Information for subjects and procedures for informed consent

E.6.1 Patient information is complete and understandable x

E.6.2 The procedures foreseen by the protocol are indicated in detail x

E.6.3 The inconveniences and risks that the patient could be exposed to are well described x

E.6.4 The methods of obtaining consent are well explained x

E.6.5 The ways of involving healthy volunteers are adequate NA x Yes

E.6.6 The measures adopted to safeguard the privacy of the subject and the protection of personal data are appropriate in accordance with current legislation x

E.6.7 The procedures for informing the attending doctor are correct and complete NA Yes x

E.6.7.1 Any critical elements found

E.7 Economic aspects and information relating to facilities and persons

E.7.1 The elements of the draft contract between the promoter and the clinical centre have been adequately assessed NA Yes x

E.7.2 The insurance coverage guarantees appropriate protection of the participants x

E.7.3 The modalities of remuneration or compensation or emoluments of any nature provided by the competent authorities for the researchers are in compliance with current regulations appropriate with respect to the required commitment and not such as to constitute a decisive element for the conduct of the trial NA Yes x

E.7.4 The appropriateness of any indemnity for healthy volunteers has been considered which must not be such as to constitute a determining factor for participation in the trial NA x Yes

E.7.5 The suitability of the researcher and their staff was examined x

E.7.6 The health facility where the study will take place is appropriate x

E.7.7 It has been verified that the promoter declares to guarantee a correct and rapid dissemination of the information acquired through the study x

E.7.7.1 Any critical elements found

F. DECISION OF THE ETHICS COMMITTEE

F.1 Single and favourable opinion Yes [x] No [ ]

F.2 In the event of a request for an opinion on a non-commercial trial, the EC has ascertained the existence of the requirements of current legislation NA [ ] Yes [x] No [ ]

F.3 Single and unfavourable opinion Yes [ ] No [ ]

F.4 Trial to be conducted at

F.4.1 Same structure Yes [x] No [ ]

F.4.2 Other structure Yes [ ] No [x]

F.5 Number of patients envisaged at the centre

54

F.6 Gross contribution expected by the promoter (per completed subject where applicable)

n.a.

179.2022.Farm.AOUBo_signed_translated into English on 8June2022

G. SPECIAL ASPECTS OF THE STUDY CONSIDERED IN THE ISSUE OF THE SINGLE FAVOURABLE OPINION

G.1 SPECIAL ASPECTS OF THE STUDY CONSIDERED IN THE ISSUE OF THE SINGLE FAVOURABLE OPINION

Having acquired the required documentation and clarifications, the Ethics Committee, in the electronic session on 18/05/2022, unanimously expresses a single opinion in favour of conducting the study.

It is understood that, in accordance with the non-profit nature of the study, the use of the data and results of the study for commercial and / or profit-making purposes is not permitted. Otherwise, should there be any scientific discoveries that can be patented or otherwise exploited commercially by any for-profit entity, it will be necessary to re-qualify the study from non-profit to profit with the consequent payment of the fee provided for the Ethics Committee and the direct and indirect costs of the study. pursuant to Legislative Decree 52/2019, subject to due information to the Committee for the issue of the opinion. Finally, it should be noted that: â ¢ the study may be started only after having received the authorisation from the General Management of the reference healthcare facility pursuant to art. 7 of L.R. no. 9/2017; â ¢ for the purposes of monitoring the progress of the study in question, the Lead Investigator must communicate the following information to the Ethics Committee regarding this single trial centre: enrolment start date, enrolment end date and study end date. In any case, starting from the year of approval of the study and until its conclusion, at least once a year and in any case no later than 31 December, an annual report on the progress of the study must be provided. For the aforementioned communications it is possible to use the form available on the website of the Ethics Committee (https://www.aosp.bo. it/files/modulo_monitoraggio_ceavec_v3_5-7-19_def.doc). The completed, signed and dated form shall be sent exclusively in electronic format to the dedicated email address monitoraggiostudi@aosp.bo.it indicating in the subject line the name of the local Lead Investigator and the code assigned by the Committee to the study.. Finally, it should be noted that the Ethics Committee is set up in compliance with Ministerial Decree 08/02/2013 on criteria
for the composition and functioning of ethics committees. A list of the members present at the evaluation is attached. The signing of the opinion on the OsSC is delegated to Dr. Giacomo Chiabrando, OsSC contact person for the CE-AVEC, as per the letter of delegation from the President dated 23/02/2022 available at the CE-AVEC registered office. List of documents examined in the session of 17/03/2022: â¢ transmission letter signed on 17/01/2022 â¢ Annex 5, v. 1.0 digitally signed on 13/01/2022 â¢ Study Protocol, v.2.0 of 04/12/2021 â¢

Synopsis of the Protocol in Italian, translated on 15/12/2021 v. 2.0 dated 04/12/2021 â¢ Investigator’s Brochure (IB) v.4.0 dated September 2021 â¢ Information and informed consent
for parents/legal guardian, v. 1.0 dated 11/11/2021 â¢ Information and informed consent for adult patients, v. 1.0 dated 11/11/2021 â¢ Information and consent for adolescent patients, v. 1.0 dated 11/11/2021 â¢

Information and consent to the processing of personal data for parents / legal guardian, v1.0 of 16/12/2021 â¢ Information and consent to the processing of personal data for adult patients, v. 1.0 dated 16/12/2021 â¢ Patient participation form v. 1.1 dated 27.07.2021 â¢ Specific centre form signed on 15/02/2022 â ¢ LUM collaboration declaration dated 12/01/2022 â¢ Availability letter for Radiology Golfieri SPIOMET4HEALTH â ¢ Availability letter for Radiology Lovato SPIOMET4HEALTH â¢ Grant Agreement 899671 of 28/01/2021 â¢ Proxy letter dated 01/10/2021 â¢ EudraCT number confirmation dated 01/06/2021 â¢ Scientific Advice EMA: EMA/CHMP/SAWP/282878/2017 of 18/05/2017 â¢ PIP Decision No. P/0150/2021 of 16/04/2021 â¢ Investigational Medicinal Product Dossier (IMPD) v.4.0 of September 2021 â¢ Production Authorisation - LABORATORY REIG JOFRÃ, S.A., Barcelona, Spain-MIA 0391-26/06/2021 â¢

GMP Certificate - LABORATORY REIG JOFRÃ, S.A., Barcelona, Spain-NCF/2120/001/CAT of 31/03/2021 â¢ Contents of the label for the investigational drug v. 2.0 dated 24/11/2021 Insurance policy dated 05/11/2021 â ¢ Insurance certificate dated 29/11/2021 â¢ Summary list of the variables of the eCRF, v. 1.0 dated 04/10/2021 â ¢ SAE notification form, v. 2.0 dated 02/11/2021 â¢ Screening of eating disorders (SCOFF + BEDS-7 questionnaire), v. 2.0 dated 30.09.2021 â¢ Brochure â¢ Intervention on lifestyles, v. 2.0 dated 05/11/2021 â¢ Storyboard, v. 1.0 dated 12/08/2021 â ¢ Description of the patient's diary, v. 1.0 dated 29/09/2021 â¢

Patient diary and tablet acceptability, see. 1.0 dated 29/09/2021 â¢ Survey for study participants, v 1.0 dated 28/07/2021 â¢ Brochure (salivary sample collection), v. 1.0 dated 25/08/2021 â¢ LIP evaluation questionnaire, v 1.0 dated 08/08/2021 â¢ Patient involvement activity flyer, v. 1.0 dated 01/07/2021 â¢ EDE-Q questionnaire, v. 6.0 of 2008 â¢ Letter for the paediatrician / GP, v. 2.0 dated 14/12/2021 â¢ Plan of the recruitment campaign, v. 1.0 of 27/11/2021 â¢ Web page, v. 2.0 dated 08/10/2021 â¢ Standard operating procedure on screening for eating disorders, see 1.0 dated 24/08/2021 â¢ Manual - Lifestyle intervention program for adolescents/young women with PCOS, v. 1.1 dated 12/08/2021 â¢ Documentary sheet - Living in health with PCOS, v. 1.0 dated 03/08/2021 â¢ List of participating centres, see 1.0 dated 25/10/2021 â¢ CV Main Investigator, Prof. Alessandra Gambineri dated 04/08/2021 â¢ GCP Certificate

G.1 SPECIAL ASPECTS OF THE STUDY CONSIDERED IN THE ISSUE OF THE SINGLE FAVOURABLE OPINION

Principal Investigator dated 27/07/2016 â¢ Declaration of absence of conflict of interest dated 11/10/2021 â¢ SPIRIT Check-list dated 05/01/2022 â¢ Declaration on the non-profit nature of the study dated 13/01/2022 â¢ Letter of response to the validation requests of 03/02/2022 â¢ Draft economic agreement in English of 05/07/2021 â¢ Grant Agreement 899671, digitally unsigned version of 28/01/2021 â¢ Self-declaration on the non-profit nature of the Promoter dated 13/09/2021 â¢ Certificate from the Spanish State Tax Administration dated 17/05/2021 â¢ Tax identification of the Promoter, FSJD â ¢ Questionnaire for self-compilation dated 01/06/2021 â¢ Text for the administration of the interview dated 03/12/2018 â¢ Translation certificate of the questionnaire dated 01/06/2021 â¢

Certificate of translation of the text for the interview administration of 03/12/2018 â¢ PCOSQ Quality of Life questionnaire List of documents examined in the session of 18/05/2022: â¢ Letter of response to clarifications by Optimapharm d.o.o. dated 11/05/2022 â¢ Annex 5 V.1.4 signed on 16/05/2022 â¢ Study Protocol V.3.0 dated 16/03/2022 â¢ Investigational Medicinal Product Dossier (IMPD): o

IMPD SPIOMET V.5.0 â March 2022 or IMPD Pioglitazone V.2.0 â March 2022 or

IMPD SPIO V.2.0 â March 2022 or IMPD Placebo V.2.0 â March 2022 â ¢

Information and informed consent forms: o Information and informed consent for parents/legal representative V.2.0 of 10/05/2022 o Information and informed consent for adult patients V.2.0 of 10/05/2022 o Information and consent for patients adolescents V.2.0 of 10/05/2022 o Information on the future use of samples for parents V.1.0 of 09/05/2022 o Information on the future use of samples for adult participants V.1.0 of 09/05/2022 â¢ Other material to be provided to the patient: o Patient participation form V1.0 dated 27/07/2021 o

Leaflet Intervention on life styles V.2.0 dated 05/11/2021 o

Description of the patient's diary V.1.0 of 29/09/2021 o Patient's diary and acceptability of tablets V.1.0 of 29/09/2021 o Survey for study participants V1.0 of 28/07/2021 o Questionnaire of LIP evaluation V.2.0 dated 14/01/2022 o

Patient Involvement Activity Flyer V.1.0 dated 01/07/2021 or Letter to the paediatrician/GP V.2.0 dated 14/12/2021 â ¢ Provisions for the recruitment of subjects: o Recruitment campaign plan V.1.0 dated 27/11/2021 or Web page V.2.0 of 08/10/2021 â¢ Letter of transmission of response to AIFA's reasoned objections dated 23/03/2022 â¢ Response to AIFA's reasoned objections dated 23/03/2022 â¢

Pioglitazone N-Nitrous Compound Statement dated 20/10/2021 â¢ NDMA (Nitrosodimethylamine) Risk Assessment Study Report and

NDEA (nitrosodiethylamine) in Metformin - 09/06/2020 â¢ Certificate of Spironolactone â suitability - 29/05/2017 â¢ Declaration of compliance with ICH Q3D guidelines for elementary impurities â Spironolactone - 02/11/2021 â¢ Declaration of origin â LIGAMED MF-2-V/K (magnesium stearate) - 15/01/2018 â¢ Statement on the results of the verification of the analytical methods - 01/03/2022 â ¢ Verification study for the determination of related substances of Metformin in SPIOMET - 12/14/2021 â¢

Study to verify the dosage and uniformity of the content of Metformin, Pioglitazone and Spironolactone in SPIOMET, SPIO and PIO tablets - 30/11/2021 â¢

Verification study for the determination of related substances of Pioglitazone and Spironolactone in SPIOMET, SPIO and PIO tablets - 25/01/2022 â¢ Verification study of the dissolution test of Metformin, Pioglitazone and Spironolactone in SPIOMET tablets - 21/02/2022 â¢ Verification study of the dissolution test of Pioglitazone and Spironolactone in SPIOMET tablets - 21/02/2022 â¢ Stability analysis of the SPIOMET H1756 batch used in the Phase I clinical study KPI-002-CL-002 - 15/04/2021 â¢ Stability study of drug batches Spiomet frasco 35 comp, Spio (Spiomet) frasco 35 comp, PIO (SPIOMET) frasco 35 comp and Placebo Spiomet frasco 35 comp batch 22101E - 16/03/2022 â¢ AIFA authorisation issued on 28/03/2022

H. REASONING FOR THE UNFAVOURABLE SINGLE OPINION

H.1 Protocol

H.1.1 Relevance of the trial ☐

H.1.2 Inclusion and exclusion criteria ☐

H.1.3 Control group ☐

H.2 Information for subjects and procedures for informed consent

H.2.1 Recruitment procedure ☐

H.2.2 Information sheet for informed consent form and procedures ☐

H.2.3 Personal data protection and confidentiality ☐

H.3 Ethical elements

H.3.1 Assessment of foreseeable risks and benefits ☐

H.3.2 Measures to minimise pain, discomfort and fear ☐

H.3.3 Inclusion of people unable to validly give their informed consent and other vulnerable populations ☐

H.3.4 Adhesion to the rules of Good Clinical Practice ☐

H.4.1 Suitability of the researcher and their collaborators ☐

H.4.2 Suitability of the health facility ☐

H.4.3 Contact between promoter and clinical centre ☐

H.4.4 Insurance policy ☐

H.4.5 Payment for study participants ☐

H.4.6 Payment for researchers ☐

H.4.7 Compliance with the administrative obligations ☐

H.5 Other Yes ☐ No ☐

H.5.1 If yes is answered to the previous point, specify

I. DESCRIPTION OF THE REASONING FOR THE UNFAVOURABLE SINGLE OPINION

L.1 Description of the reasoning for the unfavourable single opinion

L. DECISION OF THE ETHICS COMMITTEE

L.1 Date of the meeting
18/05/2022

L.2 Number of the EC opinions register
179/2022/Farm/AOUBo

L.3 Members of the EC present and qualifications
Elisabetta Poluzzi - Pharmacologist

L.3.1 Permanent substitute who attended the session in place of the Medical Director
n.a.

L.4 External consultants present and qualifications (where applicable)
n.a.

L.5 EC members present who did not participate in the vote (where applicable)
Dr. Pelusi abstains from voting due to conflict of interest

M. SIGNATURE OF THE CHAIRMAN OF THE ETHICS COMMITTEE

M.1 The Committee expressed the single opinion

M.1.1 Once the existence of the quorum has been verified, with number of members _

33

M.1.1.1 on No. ____

42

M.1.2 Having regard to any comments received from the ethics committees

n.a.

M.2 Date

27/05/2022

M.2 Forename

M.2.1 Surname

M.3 Signature

M.4 Attach file

DOCUMENTATION

Documentation	
File Name:	elenco presenti assenti CE AVEC 18.05.2022_2.pdf

De Videnskabsetiske Komitéer for Region Syddanmark

komite@rsyd.dk

Pernille Ravn
Odense universitetshospital
Gynækologisk Obstetrisk Afdeling D
Kløvervænget 23, Indgang 55, 2. sal,
5000 Odense C

Dato: 28. april 2022
Projekt-ID: **S-20220006**
Acadrenr: 22/6638.
Heidi Lund Olesen /
Betina Simonsen

Forskningsprojekt: Et randomiseret, multi-center, multinationalt klinisk fase II-forsøg med henblik på at evaluere effektiviteten, tolerancen og sikkerheden af en fast dosis kombination af spironolacton, pioglitazon og metformin (SPIOMET) til unge piger og unge voksne kvinder (AYA) med polycystisk ovariesyndrom (PCOS).

Den Videnskabsetiske Komité 2 for Region Syddanmark har nu truffet endelig afgørelse om **godkendelse af dit projekt**. Komitéen har den 13. april 2022 modtaget revideret materiale. Projektmaterialet opfylder nu vilkår givet ved afgørelsen den 4. marts 2022.

Afgørelsen er truffet i henhold til lov nr. 1338 af 1. september 2020 om videnskabsetisk behandling af sundhedsvidenskabelige forskningsprojekter (komiteloven).

Godkendelsen gælder for de anmeldte forsøgssteder, de anmeldte forsøgsansvarlige i Danmark samt for den angivne forsøgsperiode. Komitéen forventer, at den forsøgsansvarlige sørger for at underrette de øvrige deltagere i projektet om Komitéens afgørelse.

Lægemiddelstyrelsen orienteres om afgørelsen.
Det er en betingelse for projektets iværksættelse, at Lægemiddelstyrelsen også godkender forsøget.

Vær opmærksom på at godkendelsesperioden i Lægemiddelstyrelsens afgørelse kan være en anden end i denne afgørelse. Det er forsøgsansvarliges ansvar at sikre – evt. ved en ansøgning om forlængelse – at der er hele tiden under projektets forløb er fornøden godkendelse fra de to myndigheder.

Komitéen gav samtidig dispensation fra kravet om informeret samtykke for en gruppe af forsøgspersoner, som beskrevet i bilag jf. komitélovens §10, stk. 1.

Godkendelsen gælder fra 28. april 2022 til den 31. marts 2025.

Godkendelsen omfatter følgende dokumenter:

• Forsøgsprotokol	version Final 2.0	4. december 2021
• Protokoltillæg (dansk)	version vers. 2.0	13. april 2022
• Deltagerinfo. og samtykkeerklæring voksne v. 3.0		13. april 2022
• Deltagerinfo. og samtykkeerklæring 15-17 år v. 3.0		13. april 2022
• Deltagerinfo. og samtykkeerklæring forældre v. 3.0		13. april 2022
• Spørgeskema: LIP Evaluation Questions vers. 2.0		14. januar 2022
• Spørgeskema: ede-q v. 6.0		15. september 2021
• Spørgeskema: SCOFF og BEDS Screening v. 2.0		30. september 2021
• Spørgeskema: Procedurer v 1.0		28. juli 2021
• Spørgeskema: SF 36 v. 2.0 standard og interview		23. april 2019

•	Spørgeskema: PCOSQ (livskvalitet) v. 2.0	23. april 2019
•	Patient dagbog final v. 1.0	29. september 2021
•	Patient Engagement flyer final- v. 1.0	1. juli 2021
•	SPIOMET4HEALTH Webpage v. 2.0	8. oktober 2021

Følgende dokumenter er taget til efterretning i forbindelse med behandlingen af projektet:

•	Forældrefuldmagt, modtaget	23. marts 2022
•	Investigator's Brochure v. 4.0	1. september 2021
•	Brochure Lifestyle Intervention v. 2.0	5. november 2021
•	Dagbog beskrivelse final v. 1.0	29. september 2021
•	Instruks til spytprøver (flyer) final v. 1.0	25. august 2021
•	Spiomet4health_Storyboard v. 1.0	12. august 2021
•	Patient nødkort v. 1.0	27. juli 2021

Iværksættelse af projektet i strid med komitéens godkendelse kan straffes med bøde eller fængsel, jf. komitélovens §§41 og 42.

Bemærkninger
Det bemærkes, at komiteen ikke er ressortmyndighed vedr. regelsættet om databeskyttelse, og at komiteen forudsætter, at projektet gennemføres i overensstemmelse med Europa-Parlamentets og Rådets forordning nr. 2016/679 af 27. april 2016 om beskyttelse af fysiske personer i forbindelse med behandling af personoplysninger og om fri udveksling af sådanne oplysninger og databeskyttelsesloven.

Forskers pligter
Som forsøgsansvarlig skal du være opmærksom på følgende forpligtelser i forhold til komitésystemet:

Ændringer
Hvis den forsøgsansvarlige foretager væsentlige ændringer under projektets gennemførelse, skal ændringerne anmeldes til komitéen i form af tillægsprotokoller. Først når komitéens godkendelse af ændringerne er modtaget, må disse iværksættes, jf. komitélovens §27, stk. 1.

Forsøgsansvarlig skal anmelde tillægsprotokoller elektronisk på www.drvk.dk. Ved anmeldelsen skal det oprindeligt tildelte anmeldelsesnummer og adgangskode anvendes.

Se vejledning om ændringer af et godkendt forskningsprojekt:
http://www.nvk.dk/forsker/forskervejledning/vejledning-om-aendringer-i-et-godkendt-projekt

Løbende indberetning af bivirkninger og hændelser
Forsøgsansvarlig skal omgående indberette til komitéen, hvis der under projektet optræder formodet alvorlige, uventede bivirkninger (SUSARs), jf. komitélovens §30, stk. 1. Indberetningen skal ledsages af kommentarer om eventuelle konsekvenser for forsøget. Pligten til indberetning omfatter kun SUSARs, der er forekommet i Danmark og skal ske senest syv dage efter, at sponsor eller den forsøgsansvarlige har fået kendskab til tilfældet.

Indberetning af SUSARs til komitésystemet skal indholdsmæssigt følge Lægemiddelstyrelsens vejledning om kliniske forsøg, afsnit 12.1 og 12.3 om indberetning af bivirkninger.

De Videnskabsetiske Komitéer for Region Syddanmark

komite@rsyd.dk

<u>Årlig statusindberetning</u>
Én gang årligt i hele forsøgsperioden skal forsøgsansvarlig sende en årlig indberetning til komitéen. Indberetningen skal indeholde en liste over alle formodet alvorlige (ventede og uventede) bivirkninger, som er indtruffet i forsøgsperioden (ASR/DSUR) sammen med en rapport om forsøgspersonernes sikkerhed, jf. komitélovens §30, stk. 2. Materialet skal være på dansk eller engelsk.

Indberetninger af ASR/DSUR skal i øvrigt følge Lægemiddelstyrelsens vejledning om kliniske forsøg, afsnit 12.4 om indberetning af bivirkning.

Ovenstående indberetninger kan ske ved hjælp af skemaer, der findes på
http://www.nvk.dk/emner/bivirkninger/hvornaar-skal-bivirkninger-indberettes
Skemaet med bilag skal mailes til komite@rsyd.dk.

<u>Underretning om afslutning</u>
Den forsøgsansvarlige skal senest 90 dage efter datoen for godkendelsens udløb underrette komitéen herom, jf. komitélovens §31, stk. 1.

Afbryder forsøgsansvarlig sit projekt tidligere end planlagt, skal en begrundelse herfor sendes til komitéen senest 15 dage efter, at beslutningen er truffet, jf. komitélovens §31, stk. 2.

Hvis forsøgsansvarlig ikke påbegynder sit projekt, skal dette samt årsagen hertil meddeles komiteen.

Underretningen kan ske ved hjælp af et skema, der findes på
http://www.nvk.dk/forsker/indberetning-ved-afslutning-af-forsoeg
Skemaet med bilag skal mailes til komite@rsyd.dk.

Vi skal bede forsøgsansvarlige om altid at anføre **projekt id S-20220006**, ved fremsendelse af projektmaterialer til komitéen. Henvendelser vedrørende dit projekt kan rettes til komitéens sekretariat på komite@rsyd.dk.

Tilsyn:
Det er Lægemiddelstyrelsen, der fører tilsyn med lægemiddelforsøg.

Sagen har været behandlet og er endelig godkendt af komitéens sekretariat i samarbejde med

- Aia Elise Jønch, Overlæge, phd

På Komiteens vegne
venlig hilsen

Aia Elise Jønch, Overlæge, phd

Kopi til:

MajBritt Jørgensen MajBritt.jorgensen@linkmedical.eu

Lægemiddelstyrelsen på kf@dkma.dk

Region Syddanmark

Regionshuset, Damhaven 12, 7100 Vejle
Tlf.: 76638221
www.regionsyddanmark.dk/komite

CERTIFICATION OF TRANSLATION

Quote number: 2205-00396

Source document: 221799-22 EndeligGodkendelse S-20220006.pdf

Full Title of Documents: 221799-22 EndeligGodkendelse S-20220006_EN_translated 13May2022.docx

Footer: 221799-22 EndeligGodkendelse S-20220006, translated into English on 13May2022

Original Source Language of Document: Danish
Language Document is to be Translated into: English

Declaration of Translator:

I, Jan Hirš on behalf of Skrivanek s. r. o., hereby certify that the translation into English agrees with the text of the original document in Danish language, that there were no changes/corrections made to the original document neither the translation, and that it was translated by a certified translator fluent in both above mentioned languages.

I, Jan Hirš on behalf of Skrivanek s. r. o., also confirm that the above mentioned service has been rendered and that the translated document is to the best of my knowledge a true and faithful translation of the original document as provided by the client

With Best regards,

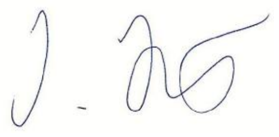

-The Scientific Ethics
Committees for the
Southern Denmark Region

komite@rsyd.dk

Pernille Ravn
Odense University Hospital
Gynækologisk Obstetrisk Afdeling D
Kløvervænget 23, Indgang 55, 2. sal,
5000 Odense C

Date: 28 April 2022
Project ID: **S-20220006**
Case no. 22/6638.
Heidi Lund Olesen /
Betina Simonsen

Research project: A randomized, multi-center, multinational Phase II clinical trial to evaluate the efficacy, tolerance and safety of a fixed dose combination of spironolactone, pioglitazone and metformin (SPIOMET) in young girls and young adult women (AYA) with polycystic ovary syndrome (PCOS).

The Scientific Ethics Committee 2 for the Southern Denmark Region has reached a conclusion on **approving your project**. On 13 April 2022, the Committee received revised material. The project materials now meet the conditions outlined in the 4 March 2022 decision.

The decision was made in accordance with Act No. 1338 of September 1, 2020, regarding the ethical treatment of health science research projects (the Committee Act).

The approval applies to the notified trial sites and trial leaders in Denmark, as well as the trial period specified. The Committee expects the individual responsible for the experiment to inform the other project participants of its decision.

The Danish Medicines Agency is informed of the decision.
The trial's approval by the Danish Medicines Agency is a prerequisite for the implementation of the project.

Please note that the approval period in the decision of the Danish Medicines Agency may differ from this decision. Throughout the duration of the project, it is the responsibility of the person in charge of the experiment to ensure - possibly in the case of a request for an extension - that there is always the necessary approval from both authorities.

At the same time, the committee exempted a group of subjects from the requirement for informed consent, as described in the appendix, cf. section 10 (1) of the Committee Act.

The approval is valid from 28 April 2022 to 31 March 2025.

The approval includes the following documents:

- Trial protocol version Final 2.0 December 4, 2021
- Protocol supplement (Danish) version verse. 2.0 April 13, 2022
- Participant info. and declaration of consent adults v. 3.0 April 13, 2022
- Participant info. and declaration of consent 15-17 years v. 3.0 April 13, 2022
- Participant info. and consent form parents v. 3.0 April 13, 2022
- Questionnaire: LIP Evaluation Questions verse. 2.0 14 January 2022
- Questionnaire: ede-q v. 6.0 15 September 2021
- Questionnaire: SCOFF and BEDS Screening v. 2.0 30 September 2021
- Questionnaire: Procedures v 1.0 July 28, 2021

Region Syddanmark
Regionshuset, Damhaven 12, 7100 Vejle
Tel.: 76638221
www.regionsyddanmark.dk/komite

- Questionnaire: SF 36 v. 2.0 standard and interview April 23, 2019
- Questionnaire: PCOSQ (quality of life) v. 2.0 23 April 2019
- Patient diary final v. 1.0 29 September 2021
- Patient Engagement flyer final- v. 1.0 01 July 2021
- SPIOMET4HEALTH Webpage v. 2.0 08 October 2021

The following documents have been noted in relation to the processing of the project:

- Parental power of attorney, received March 23, 2022
- Investigator's Brochure v. 4.0 01 September 2021
- Brochure Lifestyle Intervention v. 2.0 05 November 2021
- Diary description final v. 1.0 29 September 2021
- Instructions for saliva tests (flyer) final v. 1.0 25 August 2021
- Spiomet4health_Storyboard v. 1.0 12 August 2021
- Patient emergency card v. 1.0 27 July 2021

In accordance with Sections 41 and 42 of the Committee Act, initiating the project without approval from the committee may result in a fine or imprisonment.

Comments
It is noted that the committee is not a data protection authority and that the committee assumes the project is implemented in accordance with Regulation (EU) 2016/679 of the European Parliament and of the Council of 27 April 2016 on the protection of natural persons with regard to the processing of personal data and on the free movement of such data and with the Data Protection Act.

Duties of researchers
As the experiment's responsible party, you must be aware of the following responsibilities regarding the committee system:

Changes
If the person in charge of the experiment makes significant changes during the project's execution, the committee must be notified in the form of additional protocols. The changes may be implemented only after receiving the committee's approval, as per Section 27 (1) of the Committee Act.

The person responsible for the experiment must notify additional protocols electronically at www.drvk.dk.
When notifying, the originally assigned notification number and password must be used.

See guidance on changes to an approved research project:
http://www.nvk.dk/forsker/forskervejledning/vejledning-om-aendringer-i-et-godkendt-projekt

Continuous reporting of side effects and events
According to section 30 (1) of the Committee Act, the person in charge of the experiment must immediately notify the committee of any serious, unanticipated side effects (SUSARs) that occur during the project. The report must include commentary on any trial-related consequences. The obligation to report applies only to SUSARs that have occurred in Denmark and must be fulfilled within seven days of the sponsor or person responsible for the trial becoming aware of the incident.

In terms of content, the reporting of SUSARs to the committee system must adhere to the Danish Medicines Agency's clinical trial guidelines, sections 12.1 and 12.3 on reporting side effects.

-The Scientific Ethics
Committees for the
Southern Denmark Region

komite@rsyd.dk

Annual status report
Throughout the entire trial period, the individual in charge of the trial must submit an annual report to the committee. The report must include a list of all presumed serious (expected and unexpected) adverse events that occurred during the trial period (ASR / DSUR) as well as a report on the subjects' safety, per Section 30 (2) of the Committee Act. The material must be in Danish or English.

Reports of ASR / DSUR must otherwise adhere to the Danish Medicines Agency's clinical trial reporting guidelines, as per section 12.4 on adverse reactions.

The above reports can be made using forms available at http://www.nvk.dk/emner/bivirkninger/hvornaar-skal-bivirkninger-indberettes
The form with appendices must be emailed to komite@rsyd.dk.

Completion notification
The individual responsible for the experiment must notify the committee within ninety days of the expiration date of the approval, as per Section 31 (1) of the Committee Act.

Section 31 (2) of the Committee Act stipulates that a justification must be sent to the committee no later than 15 days after a decision has been made if the person responsible for the experiment interrupts his project earlier than planned.

If the person responsible for the experiment does not start his project, this and the reason for this must be notified to the committee.

The notification can be made using a form available at http://www.nvk.dk/forsker/indberetning-ved-afslutning-af-forsoeg
The form with appendices must be emailed to komite@rsyd.dk.

We must ask the person responsible for the experiment to always state **project id S-20220006** , when sending project materials to the committee. Inquiries regarding your project can be directed to the committee's secretariat at komite@rsyd.dk.

Oversight:
The Danish Medicines Agency supervises drug trials.

The case has been considered and has finally been approved by the committee's secretariat in collaboration with

- Aia Elise Jønch, Overlæge, phd

Yours sincerely, on behalf of the Committee

Aia Elise Jønch, Overlæge, phd

Copy to:

MajBritt Jørgensen MajBritt.jorgensen@linkmedical.eu

Region Syddanmark
Regionshuset, Damhaven 12, 7100 Vejle
Tel.: 76638221
www.regionsyddanmark.dk/komite

-The Scientific Ethics Committees for the Southern Denmark Region

komite@rsyd.dk

The Danish Medicines Agency at kf@dkma.dk

Region Syddanmark

Regionshuset, Damhaven 12, 7100 Vejle
Tel.: 76638221
www.regionsyddanmark.dk/komite

Region:	Saksbehandler:	Telefon:	Vår dato:	Vår referanse:
REK midt	Linda Tømmerdal Roten	73597506	23.06.2022	423718

Eszter Vanky

Prosjektsøknad: En randomisert, multinasjonal, fase II-multisenterstudie for å evaluere effekt, toleranse og sikkerhet for en fast kombinasjon av spironolakton, pioglitazon og metformin (SPIOMET) hos tenåringer og unge voksne kvinner med polycystisk ovariesyndrom (PCOS).
Søknadsnummer: 423718
EudraCT-nummer: 2021-003177-58
Forskningsansvarlig institusjon: Norges teknisk-naturvitenskapelige universitet

Prosjektsøknad godkjennes med vilkår

Søkers beskrivelse

Det finnes foreløpig ingen godkjent behandling for polycystisk ovariesyndrom (PCOS), den vanligste endokrine forstyrrelsen hos fertile kvinner. Sykdommen rammer 5-10% kvinner på verdensbasis. Formålet med denne studien er å evaluere effekt og sikkerhet av metformin, pioglitazon og spironolakton (SPIOMET) i kombinasjon hos jenter og unge kvinner i alderen 12-25 år med PCOS. Pasientene randomiseres 1:1:1:1 til studiens 4 behandlingsarmer; pioglitazon (PIO), kombinasjon pioglitazon og spironolakton (SPIO), kombinasjon pioglitazon, spironolakton og metformin (SPIOMET), samt placebo. Pasientene mottar behandling i 12 måneder og følges opp i ytterligere 6 måneder. Totalt skal det rekrutteres 364 pasienter i studien, hvorav 41 i Norge

Innledning

Viviser til dinsøknadom forhåndsgodkjenningavovennevnteforskningsprosjekt.Søknaden og tilbakemeldingen dinble behandlet av Regional komité for medisinsk og helsefaglig forskningsetikkMidt-Norge (REK midt)i møtet02.06.2022.Vurderingen er gjort med hjemmel i helseforskningsloven § 10.

Saksgang

Søknaden ble første gang behandlet i REK midts møte16.02.2022. Endelig vedtak ble utsatt fordi det var uklarheter knyttet til deltakernes risiko. Komiteen besluttet å innhente en sakkyndiguttalelse til støtte i den videre vurderingen av forsvarligheten av prosjektet. Prosjektleder ble bedt om en tilbakemelding på om det er nødvendig å inkludere mindreårige i prosjektet, om en betenkning rundt det å gi livsstilsråd til mindreårige og om spørreskjemaer og informasjonsmateriell bør tilpasses de yngste i utvalget. Videre har vi

bedt prosjektleder klargjøre om det skal opprettes en spesifikk forskningsbiobank i Norge og om sponsor skal kunne bruke data til å besvare fremtidige forskningsspørsmål utover de som er beskrevet i søknaden. Vi mottok sakkyndiguttalelser 20.05.2022 og 24.05.2022, og tilbakemeldingen fra prosjektleder 20.05.2022. Et spørreskjema (SCOFF /BEDS v2) med spørsmål for å avdekke spiseforstørrelser ble ettersendt 20.05.2022, men dette kom ikke med på komitémøtet.

REKs vurdering

Komiteens opprinnelige prosjektsammendrag

Polycystisk ovariesyndrom (PCOS) er den vanligste endokrine forstyrrelsen hos fertile kvinner. Formålet med denne internasjonale fase II multisenter randomiserte legemiddelstudien er å undersøke hvordan metformin, pioglitazon og spironolakton i kombinasjon (SPIOMET) påvirker eggløsningsrate hos jenter og unge kvinner i alderen 12-25 år med PCOS, og evaluere effekt, toleranse og sikkerhet av behandlingen. Det er fire studiearmer: 1: placebo, 2: pioglitazone, 3: kombinasjon av pioglitazone og spironolakton, og 4: kombinasjon av pioglitazone, spironaolakton og metformin. Totalt skal man rekruttere 364 pasienter, hvorav 41 fra St. Olavs Hospital, Norge. Deltakerne blir randomisert til en av de fire studiearmene. Deltakelse innebærer å motta behandling i 12 måneder, kliniske undersøkelser som røntgen for å undersøke kroppssammensetning (DXA), MR for å måle andelen fett i organer (innvoller), under huden samt i lever, ultralyd av halspulsåre og måling av høyde, vekt og blodtrykk. Det vil også bli tatt blodprøver, spyttprøver for å måle progesteron og urinprøver for graviditetstest. Biologisk materiale som ikke analyseres kort tid etter prøvetaking skal lagres i en spesifikk forskningsbiobank. Opplysninger om medisinsk historie og medisinbruk vil bli hentet fra pasientjournal, og deltakerne skal fylle ut spørreskjema og menstruasjonsdagbok. Studien er samtykkebasert. EudraCT-nummer 2021-003177-58.

Oppsummering av tilbakemeldingen

I tilbakemeldingen har du kommentert komiteens punkter. Du presiserer at det er nødvendig å inkludere mindreårige i studien, da det er jenter under 19 år som har minst behandlingstilbud. Du har imidlertid oppgitt at det er uaktuelt å inkludere jenter under 14 år i studien. All livsstilscoaching vil bli individuelt tilpasset den enkelte deltaker. Spørreskjema om spiseforstyrrelser (EDE-Q) vil kun bli benyttet dersom studiesykepleier får bekreftet at det er risiko for spiseforstyrrelser via skjemaet «SCOFF+BEDS». Du opplyser om at det ikke skal opprettes noen forskningsbiobank i Norge. Alt biologisk materiale vil bli overført til Spania, og deltakerne blir gitt mulighet til å velge om de ønsker å donere materiale til lagring i Spania for fremtidig forskning på PCOS.

Oppsummering av sakkyndiges vurdering

Begge de sakkyndige vurderer at studien er medisinsk forsvarlig å gjennomføre, gitt en bedre plan for å håndtere spiseforstyrrelser og med endringer i informasjonsmateriellet. En

av de sakkyndige mener sikkerheten rundt prevensjon og tidlig påvisning av svangerskap må være bedre. Videre er en av de sakkyndige skeptisk til inklusjon av jenter ned i 12-års alder, og mener det ikke er nødvendig for å besvare forskningsspørsmålene.

Komiteens forsvarlighetsvurdering

Vi ber deg utarbeide en plan for å håndtere spiseforstyrrelser, og ber om endringer i kost/livsstilskjemaet, skjemaet med tittel «Din helse og trivsel» og informasjonsskriv. Vi har også en kommentar til lagring av humant biologisk materiale i utlandet. Du må også oppdatere forskningsprotokollen. Forutsatt at du endrer informasjonsskriv i samsvar med våre kommentarer vil deltakerne og de foresatte motta informasjon som gir dem et godt grunnlag for et informert, frivilligog dokumenterbart samtykke. Vi gjør oppmerksom på at samtykker som blir avgitt på grunnlag av informasjonsskrivene ikke er dekkende for å utføre genetiske undersøkelser. Vi vurderer atrisikoen/ulempen forbundet med deltakelse er minimal og akseptabel gitt den potensielle nyttenav prosjektet. Prosjektet er også organisert med en klar ansvarsfordeling, og med relevant og tilstrekkelig kompetanse i prosjektgruppen. Under forutsetning av at vilkårene nedenfor tas til følge vurderer vi at prosjektet er forsvarlig, og at hensynet til deltakernes velferd og integritet er ivaretatt.

Godkjenner opprettelse av spesifikk forskningsbiobank

Det skal opprettes en spesifikk forskningsbiobank bestående av blodprøver (fullblod, serum og plasma), urin og spyttprøver fra inntil 30 personer. Du, som prosjektleder, er ansvarshavende for forskningsbiobanken. Vi godkjenner opprettelsen av en spesifikk forskningsbiobank jf. helseforskningsloven § 25, annet ledd. Du har i søknadsskjemaets punkt 5.15 oppgitt at dere ønsker å oppbevare biologisk materiale utover sluttdato for prosjektet. Vi ønsker å presisere at det ikke er anledning til å oppbevare biologisk materiale som lagres i en norsk prosjektspesifikk forskningsbiobank utover prosjektperioden. Vi setter en tidsavgrensning på forskningsbiobanken i tråd med sluttdatoen for prosjektet, til og med 30.08.2025. Du er ansvarlig for at materialet destrueres senest ved sluttdato.

Lagring av biologisk materiale i utlandet

Du har opplyst om at biologisk materiale skal overføres til Spania for lagring i forskningsbiobank for fremtidig forskning på PCOS (generell forskningsbiobank) dersom deltaker/foresatte samtykker til det. Vi gjør oppmerksom på at norsk REK ikke har myndighet til å godkjenne opprettelse av generell forskningsbiobank i utlandet, eller stille vilkår knyttet til hvor lenge materialet skal lagres i utlandet. Vanligvis lagres biologisk materiale permanent i en generell forskningsbiobank. REK skal imidlertid påse kravene til samtykke er oppfylt og at deltakerne får god og tilstrekkelig informasjon. Vi vurderer at kravene i dette tilfellet er oppfylt, jamfør helseforskningsloven § 29. Vi godkjenner derfor overføringen av humant biologisk materiale til Spania.

Plan for å håndtere spiseforstyrrelser

Vi ber om at du utarbeider en beredskapsplan for å håndtere spiseforstyrrelser som blir avdekket. Vi ber deg også om å involvere en ernæringsfysiolog til å gå igjennom informasjonsmateriellet for å se om det er formuleringer som kan stimulere til spiseforstyrrelser.

Endring av spørreskjema

Du må endre kost/livsstilskjemaet ved å bytte ut ordene «lidelse» med «tilstand» og «fertilitet» med «fruktbarhet». Du må endre skjemaet med tittel «Din helse og trivsel» ved å endre setningen «For hvert av de følgende spørsmålene vennligst sett et x i den ene luken som best beskriver ditt svar.» ved å bytte ut ordet «luken» med for eksempel «avkryssingsboks».

Endring av informasjonsskriv

Vi ber deg om å utforme et kort og forenklet informasjonsskriv tilpasset barn der du unngår fagterminologi, samt ord og begreper som ikke nødvendigvis er enkle å forstå for barn og ungdom (for eksempel produksjon, effekt, relatert). Videre ber vi deg om å endre informasjonsskriv i samsvar med følgende punkter:

1. Oppdater informasjonsskriv til samtykkekompetente ved å ta utgangspunkt i REKs mal for informasjonsskriv til voksne».
2. Det må komme klart frem at NTNU og St. Olavs Hospital er ansvarlig for studien i Norge.
3. Informer nærmere om prevensjon og graviditetstesting på en måte som ikke forutsetter at alle er seksuelt aktive. Du kan for eksempel informere om at dersom man er seksuelt aktiv så vil det være aktuelt med graviditetstesting på grunn av legemidlene.
4. Beskriv livsstilsintervensjonen bedre, og gjerne i samråd med en ernæringsfysiolog.
5. I skrivet til foresatte må du endre setningen «Dette er et spørsmål til din datter om å delta i et forskningsprosjekt for å undersøke…» til «Dette er et spørsmål til deg om du vil la din datter delta i et forskningsprosjekt for å undersøke…».
6. Unngå fagterminologi. Bytt ut «hirsutisme» med «økt hårvekst».
7. Bytt ut «dårlig kolesterol» med «ugunstig kolesterol».
8. Du må klargjøre at lagring i en generell forskningsbiobank innebærer permanent lagring av materialet. Du må også omformulere setningen «Opplysningene og prøvene av deg vil bli slettet 15 år etter at sluttrapport etter studien foreligger.» ved å presisere at slettingen kun gjelder i denne konkrete studien.
9. Endre setningen «Etter ny personopplysningslov har Fundació Sant Joan de Déu (FSJD) behandlingsansvar for dine personopplysninger og prosjektleder Eszter Ilona Vanky et selvstendig ansvar for å sikre at behandlingen av dine opplysninger har et lovlig grunnlag.» til «NTNU, St. Olavs Hospital og prosjektleder Eszter Ilona Vanky er ansvarlig for personvernet i prosjektet.».
10. Bytt ut setningen «Vi behandler opplysningene basert på og i samsvar med artikkel 6 til 9 i EUs generelle personvernforordning.» med «Vi behandler opplysningene basert på samtykke.».
11. Vennligst oppdater versjonsnummer og/eller datering i skrivene.

Vilkår for godkjenning

1. Du må sendeossreviderteinformasjonsskriv og forenklet skriv til barn, kost/livsstilskjema, skjemaet med tittelen «Din helse og trivsel» og beredskapsplanen viaREK-portalen.Vennligst benytt funksjonen«Endring og/eller henvendelse».Du kan ikke sette i gang prosjektet før vi har bekreftet atinformasjonsskriveneer godkjent.
2. Du må registrere St. Olavs Hospital HF hvor pasientene rekrutteres fra som forskningsansvarlig institusjon i tillegg til NTNU. Vennligst benytt funksjonen«Endring og/eller henvendelse».
3. Komiteen forutsetter at kun jenter over 14 år inkluderes i studien.
4. Du kan kun bruke materialet i biobanken i dette konkrete prosjektet.Annen brukvil kreve søknad tiloss, og vil normalt sett kreve innhenting av nytt samtykke.
5. Materiale i forskningsbiobanker skal oppbevares og behandles forsvarlig. Oppbevaring og behandling skal skje med respekt for giveren av materialet, jf. helseforskningsloven § 27.
6. Komiteen minner om at koblingsnøkkel skal bli værende i Norge ved internasjonalt samarbeid. Dette gjelder også ved lagring av biologisk materiale i utlandet.
7. Komiteen forutsetter godkjenning fra Legemiddelverket (SLV) dersom dette er nødvendig. Du er selv ansvarlig for å avklare dette med SLV.
8. Komiteen minner om at de aller fleste kliniske studier skal registreres i det offentlig tilgjengelige registeret www.clinicaltrials.gov. Du er selv ansvarlig for å avklare om forskningsstudien er omfattet av kravet til registrering.
9. Komiteen forutsetter at ingen personidentifiserbare opplysninger kanfremkommeved publisering eller annen offentliggjøring.
10. Komiteen forutsetter at du og alle prosjektmedarbeiderne følger egen institusjons bestemmelser for å ivareta informasjonssikkerhet og personvern ved innsamling, bruk, oppbevaring, deling og utlevering av personopplysninger. Bestemmelsene må være i samsvar medkomiteensvilkår for godkjenning.
11. Av dokumentasjonshensyn skal opplysningene oppbevares i 15 år etter prosjektslutt. Enhver tilgang til prosjektdataene skal da være knyttet til behovet for etterkontroll. Prosjektdata vilsåledesikke være tilgjengelig for prosjektet. Prosjektleder og forskningsansvarlig institusjon er ansvarlige for at opplysningene oppbevares indirekte personidentifiserbart i denne perioden, det vil siatskilt i enkoblingsnøkkel- og en datafil.Etter denne 15-årsperioden skal opplysningene slettes eller anonymiseres. Komiteen gjør oppmerksom på at anonymisering er mer omfattende enn å kun slette koblingsnøkkelen, jf. Datatilsynets veileder om anonymiseringsteknikker.

Vedtak

Godkjent på vilkår

Sluttmelding

Prosjektleder skal sende sluttmelding til REK på eget skjema via REK-portalen senest 6 måneder etter sluttdato 30.08.2025, jf. helseforskningsloven § 12. Dersom prosjektet ikke starter opp eller gjennomføres meldes dette også via skjemaet for sluttmelding.

Søknad om endring
Dersom man ønsker å foreta vesentlige endringer i formål, metode, tidsløp eller organisering må prosjektleder sende søknad om endring via portalen på eget skjema til REK, jf. helseforskningsloven § 11.

Klageadgang
Du kan klage på REKs vedtak, jf. forvaltningsloven § 28 flg. Klagen sendes på eget skjema via REK portalen. Klagefristen er tre uker fra du mottar dette brevet. Dersom REK opprettholder vedtaket, sender REK klagen videre til Den nasjonale forskningsetiske komité for medisin og helsefag (NEM) for endelig vurdering, jf. forskningsetikkloven § 10 og helseforskningsloven § 10.

Med vennlig hilsen

Vibeke Videm

Professor, dr.med./ overlege

Leder, REK midt

Linda Tømmerdal Roten

Seniorrådgiver, REK midt

Kopi til:

Norges teknisk-naturvitenskapelige universitet
Inger Hilde Zahl
Statens legemiddelverk

REK
REGIONALE KOMITEER FOR MEDISINSK OG HELSEFAGLIG FORSKNINGSETIKK

Region:	Saksbehandler:	Telefon:	Vår dato:	Vår referanse:
REK midt	Linda Tømmerdal Roten	73597506	16.08.2022	423718

Eszter Vanky

423718 En randomisert, multinasjonal, fase II-multisenterstudie for å evaluere effekt, toleranse og sikkerhet for en fast kombinasjon av spironolakton, pioglitazon og metformin (SPIOMET) hos tenåringer og unge voksne kvinner med polycystisk ovariesyndrom (PCOS).

Forskningsansvarlig: Norges teknisk-naturvitenskapelige universitet

Søker: Eszter Vanky

REKs svar på generell henvendelse

Hei Ezster.
Vi mottok 04.08.2022 din henvendelse der forenklet informasjonsskriv til barn under 12 år, reviderte informasjonsskriv (versjon 1.2 datert 05.07.2022), endret kost/livsstils-skjema, spørreskjemaet SCOFF og en beredskapsplan for å håndtere spiseforstyrrelser var vedlagt. Du har endret informasjonsskrivene i samsvar med våre kommentarer. Vi anser derfor vilkår 1 i vedtaksbrev fra REK midt datert 23.06.2022 som oppfylt og tar skrivene til orientering. Videre tar vi endrede spørreskjema og beredskapsplanen til orientering. St. Olavs Hospital HF er nå registrert som forskningsansvarlig institusjon i tillegg til NTNU.

Lykke til med prosjektet.

Vennlig hilsen
Linda Tømmerdal Roten
Seniorrådgiver, REK midt

Vennlig hilsen
Regionale komiteer for medisinsk og helsefaglig forskningsetikk

Denne e-posten er sendt automatisk fra REK og kan ikke besvares

CERTIFICATION OF TRANSLATION

Quote number: 2207-01360

Source document: REK_23JUN2022 Prosjektsøknad godkjennes med vilkår.pdf

Full Title of Documents: SPIOMET4HEALTH, NOR conditional approval, 23Jun2022

Footer: SPIOMET4HEALTH, NOR conditional approval, 23Jun2022

Original Source Language of Document: Norwegian
Language Document is to be Translated into: English

Declaration of Translator:

I, Jan Hirš on behalf of Skrivanek s. r. o., hereby certify that the translation into English agrees with the text of the original document in Norwegian language, that there were no changes/corrections made to the original document neither the translation, and that it was translated by a certified translator fluent in both above mentioned languages.

I, Jan Hirš on behalf of Skrivanek s. r. o., also confirm that the above mentioned service has been rendered and that the translated document is to the best of my knowledge a true and faithful translation of the original document as provided by the client

With Best regards,

Jan Hirš
Head of IPMC and Localization Department

SKRIVANEK

Skřivánek s.r.o.
Veveří 102
CZ 616 00 Brno
jan.hirs@skrivanek.com, www.skrivanek.com
Tel.: +420 546 210 973 Fax: +420 546 210 738
GSM: +420 739 391 791

Region:	Case Officer:	Phone:	Our date:	Our reference:
REC Central	Linda Tømmerdal Roten	+47 73597506	23/06/2022	423718

Eszter Vanky

Project application: A randomised, multinational, Phase II multicentre study to evaluate the efficacy, tolerance and safety of a fixed combination of spironolactone, pioglitazone and metformin (SPIOMET) in adolescent and young adult women with polycystic ovary syndrome (PCOS).
Application number: 423718
EudraCT Number: 2021-003177-58
Institution responsible for research: The Norwegian University of Science and Technology (NTNU)

Project application is approved with conditions

Applicant's description

There is currently no approved treatment for polycystic ovary syndrome (PCOS), the most common endocrine disorder among fertile women. The disease affects 5-10% of women worldwide. The purpose of this study is to assess the efficacy and safety of metformin, pioglitazone, and spironolactone (SPIOMET) when used in combination, in girls and young women aged 12-25 years with PCOS. Patients are randomised 1:1:1:1 to the study's 4 treatment arms; pioglitazone (PIO), combination of pioglitazone and spironolactone (SPIO), combination of pioglitazone, spironolactone and metformin (SPIOMET), as well as a placebo. Patients are treated for 12 months and then monitored for an additional 6 months. The study will enrol 364 patients, 41 of whom will be from Norway.

Introduction

We refer to your application for the above research project's prior approval. The Regional Committee for Medical and Health Research Ethics in Central Norway (REC Central) considered the application and comments at its meeting on 2 June 2022. The evaluation was conducted in accordance with Section 10 of the Health Research Act.

Proceedings

The application was initially discussed at the REC meeting on 16 February 2022. The final decision was postponed due to ambiguities regarding the risk of the participants. The Committee decided to solicit an expert opinion in support of the project's continued evaluation. The project manager was requested to comment on the necessity of including

minors in the project, a report on providing lifestyle advice to minors, and whether questionnaires and informational materials should be tailored to the youngest participant in the sample. In addition, we have requested clarification from the project manager as to whether a specific research biobank is to be established in Norway and whether the sponsor will be able to use data to answer future research questions beyond those outlined in the application. We obtained expert opinions on 20 May 2022 and 24 May 2022, and project manager feedback on 20 May 2022. On 20 May 2022, a questionnaire (SCOFF / BEDS v2) containing questions to identify eating enlargements was sent, but it was not discussed at the committee meeting.

REC's assessment

The Committee's original project summary

Polycystic ovary syndrome (PCOS) is the most common endocrine disorder among fertile women. The purpose of this international Phase II multicentre randomised pharmaceuticals study is to investigate how metformin, pioglitazone and spironolactone in combination (SPIOMET) affect the ovulation rate in girls and young women aged 12-25 years with PCOS, and to evaluate the efficacy, tolerance and safety of the treatment. There are four study arms: 1: placebo, 2: pioglitazone, 3: combination of pioglitazone and spironolactone, and 4: combination of pioglitazone, spironaolactone and metformin. A total of 364 patients will be recruited, 41 of whom will come from Norway's St. Olav's Hospital. Participants are randomised into one of the four study arms. Participation in the study receive 12 months of treatment and clinical examinations such as X-rays to determine body composition (DXA), MRIs to determine the proportion of fat in organs (intestines), beneath the skin, and in the liver, carotid artery ultrasounds, and measurements of height, weight, and blood pressure. Additionally, blood samples, saliva samples for progesterone measurement, and urine samples for pregnancy testing will be taken. Biological samples that are not analysed immediately after collection must be stored in a designated research biobank. Information about medical history and medication use will be obtained from the patient record, and participants will complete a questionnaire and maintain a menstruation diary. The study is consent-based. EudraCT Number 2021-003177-58.

Summary of the feedback

You have commented on the Committee's points in the feedback. You emphasise the importance of including minors in the study because girls under 19 have the fewest treatment options. You have stated, however, that it is irrelevant to include girls younger than 14 in the study. All lifestyle coaching will be personalised to the specific participant. Questionnaire on eating disorders (EDE-Q) will only be administered if the study nurse receives confirmation via "SCOFF + BEDS" that there is a risk of eating disorders. You state that there will be no research biobank established in Norway. All biological samples will be transferred to Spain, and participants will be given the option to donate samples for future PCOS research.

Summary of expert assessment

Both experts believe it is medically justifiable to conduct the study, given a more effective treatment plan for eating disorders and revised informational materials. One of the experts believes that contraception and early pregnancy detection must be made safer. In addition, one of the experts is sceptical of the inclusion of girls under the age of 12 and believes their participation is unnecessary to answer the research questions.

The Committee's assessment of soundness

We request that you prepare a plan for addressing eating disorders and request modifications to the diet/lifestyle form, the “Your health and well-being” form, and the information leaflet. Additionally, we have a comment regarding the overseas storage of human biological material. You are also obliged to update the research protocol. If you modify the information leaflet in accordance with our suggestions, the participants and their parents will receive information that provides a solid foundation for their informed, voluntary, and documentable consent. We would emphasise that consents based solely on the information leaflets are insufficient for genetic testing. In light of the project's potential benefits, we believe the risk/disadvantage associated with participation is minimal and acceptable. The project is also organised with a clear division of responsibilities and a project team with relevant and sufficient expertise. Assuming that the conditions are met, we believe that the project is justifiable and that the well-being and integrity of the participants will have been taken into account.

Approves the establishment of a particular research biobank

Blood samples (whole blood, serum, and plasma), urine, and saliva samples will be collected from up to thirty individuals to establish a research biobank. As the project manager, you are in charge of the research biobank. We approve the establishment of a specific research biobank in accordance with Section 25 of the Health Research Act. You indicated in paragraph 5.15 of the application form that you intend to store biological material beyond the project's termination date. We wish to emphasise that it is not possible to store biological material in a project-specific biobank in Norway beyond the duration of the project. We established a time limit for the research biobank in accordance with the project's completion date of 30 August 2025. You are responsible for ensuring the material is destroyed by the expiration date.

Storage of biological material abroad

You have stated that biological material will be transferred to Spain for storage in a research biobank for future PCOS research (general research biobank) subject to consent by the participant/guardian. We note that the Norwegian REC does not have the authority to approve the establishment of a biobank for general research abroad or to set conditions regarding the length of time the material may be stored abroad. Typically, biological material is stored permanently in a biobank for general research. However, the REC must ensure that the consent requirements are met and that participants receive sufficient and accurate information. We believe the requirements in this instance have been met in

accordance with Section 29 of the Health Research Act. The transfer of human biological material to Spain is therefore approved.

Plan to address eating disorders

We request that you develop a contingency plan to address any detected eating disorders. Involve a nutritionist in reviewing the informational materials to determine if any formulations can stimulate eating disorders.

Amendments to the questionnaire

You need to modify the diet/lifestyle questionnaire by replacing the words “disorder” with “condition” and “fertility” with “fruitfulness”. You need to modify the form entitled “Your health and well-being” by replacing the word “hatch” with “check box” in the sentence “For each of the following questions, please place an X in the box that best describes your answer.”

Amendment of information letter

We request that you create a brief and simplified letter of information tailored to children, avoiding technical terminology as well as words and concepts that are not necessarily simple for children and adolescents to comprehend (.e.g., production, effect, related). In addition, we request that you modify the information sheet to reflect the following points:

1. Update consent-competent information letter using REC's template for adult information letter.
2. It must be clear that NTNU and St Olav's Hospital are responsible for the study in Norway.
3. Inform in greater detail about contraception and pregnancy testing without assuming that all individuals are sexually active. You can inform them, for instance, that if they are sexually active, pregnancy testing is necessary because of the effects of the drugs.
4. Improve the description of the lifestyle intervention, preferably in consultation with a nutritionist.
5. Change “This is a question for your daughter to participate in a research project to investigate...” to “This is an invitation for you if you would like your daughter to participate in a research project to investigate...” in the letter to parents.
6. Avoid technical terminology. Replace “hirsutism” with “increased hair growth”.
7. Replace “bad cholesterol” with “unfavourable cholesterol levels”.
8. You need to specify that storage in a biobank for general research involves long-term preservation of the material. You must also rephrase the sentence “Your information and tests will be deleted 15 years after the study's final report is published” to emphasise that the deletion only applies to this particular study.
9. Change the sentence “According to the new Personal Data Act, Fundació Sant Joan de Déu (FSJD) has processing responsibility for your personal data, and project

manager, Eszter Ilona Vanky has an independent responsibility to ensure that the processing of your data is legal" to "NTNU, St. Olav's Hospital and project manager, Eszter Ilona Vanky is responsible for the privacy of the project."

10. Replace "We process the information in accordance with and based on Articles 6 to 9 of the EU General Data Protection Regulation" with "We process the information based on consent."

11. Please update version number and/or dating in the letters.

Conditions for approval

1. Via the REC portal, you are required to send us the revised information letter and simplified letter for children, the diet/lifestyle form, the form entitled "Your health and well-being," and the contingency plan. Please use the "Change and/or inquiry" function. You may not begin the project until we have approved the information letter.
2. You must register St. Olav's Hospital HF from where the patients are recruited as the institution responsible for research in addition to NTNU. Please use the function "Change and/or inquiry".
3. The Committee assumes that the study only includes women older than 14 years of age.
4. The material in the biobank may only be used for this specific project. Other uses will necessitate an application and, in most cases, a new consent.
5. Materials in biobanks must be properly stored and handled. In accordance with Section 27 of the Health Research Act, the donor of the material must be given due consideration with respect to its storage and processing.
6. Through international cooperation, the connection key will remain in Norway, according to the Committee. This also applies to storage of biological material abroad.
7. If necessary, the Committee requires approval from the Norwegian Medicines Agency (SLV). You are responsible for clarifying this with SLV.
8. The Committee reminds you that the vast majority of clinical trials should be registered in the publicly available register www.clinicaltrials.gov. You are responsible for clarifying whether the registration requirement applies to the research study.
9. The Committee assumes that no personally identifiable information can be obtained through publication or any other means.
10. The Committee assumes that you and all project staff adhere to the information security and privacy regulations of your own institution when collecting, using, storing, sharing, and disclosing personal information. The provisions must be in accordance with the co-committee's terms for approval.
11. The information must be stored for 15 years after the completion of the project for documentation purposes. Any access to the project's data must then be correlated with the need for subsequent action. As a result, project data will not be available. The project manager and the institution responsible for the research have responsibility for ensuring that the information is stored in a connection key and a data file during this time period. After 15 years, the information must be deleted or made anonymous. The Committee points out that anonymization is more comprehensive than simply deleting the connection key, cf. The Norwegian Data Protection Authority's guide on anonymization techniques.

Decision

Conditionally Approved

Notification of Completion
The project manager is required to submit a notification of completion to the REC on a separate form via the REC portal no later than six months after the 30 August 2025 end date in accordance with Section 12 of the Health Research Act. If the project does not commence or is completed, this needs also to be communicated via the notification form.

Application for change
If you wish to make significant changes to the purpose, method, time course, or organisation, the project manager must submit a separate form via the portal to the REC in accordance with Section 11 of the Health Research Act.

Right of appeal
You may file an appeal against the REC's decision in accordance with Section 28 et seq of the Public Administration Act. The complaint is to be filed on a separate form via the REC portal. The deadline for filing complaints is three weeks after receiving this letter. If the REC upholds the decision, it forwards the complaint to the National Research Ethics Committee for Medicine and Health Sciences (NEM) for a final evaluation in accordance with Section 10 of the Research Ethics Act and Section 10 of the Health Research Act.

Best regards,

Vibeke Videm

Professor, Dr. Med./ Chief Physician

Director, REC Central

Linda Tømmerdal Roten

Senior Advisor, REC Central

Copy to:

The Norwegian University of Science and Technology (NTNU)
Inger Hilde Zahl
The Norwegian Medicines Agency

Medizinische Universität Graz
Ethikkommission

Auenbruggerplatz 2, A-8036 Graz
ethikkommission@medunigraz.at
Tel.: +43 / 316 / 385-13928, Fax: -14348

VOTUM

gültig bis 12.05.2023

**EK-Nummer:**	34-169 ex 21/22 **EudraCT Nr.:** 2021-003177-58<br>1608-2021
**Studientitel:**	A Phase II, randomised, multi-centric, multi-national clinical trial to evaluate the efficacy, tolerability, and safety of a fixed dose combination of Spironolactone, Pioglitazone & Metformin (SPIOMET) for adolescent girls and young adult women (AYAs) with polycystic ovary syndrome (PCOS).
**Prüfer:**	Professor Dr. med. Barbara Obermayer-Pietsch<br>MU Graz,Universitätsklinik für Innere Medizin, Abt. Endokrinol. und Diabetologie
**Sponsor:**	Fundació Sant Joan de Déu
Ansprechpartner:	Prof. Lourdes Ibáñez, 08950 Esplugues de Llobregat, C/ Santa Rosa, 39-57, 4a planta
**CRO:**	Optimapharm d.o.o.
Ansprechpartner:	MPharm Vladimir Vujovic, 10000 Zagreb, Ulica grada Vukovara 284
**Antragsteller:**	Optimapharm d.o.o.
Ansprechpartner:	MPharm Vladimir Vujovic, 10000 Zagreb, Ulica grada Vukovara 284

Die o.a. Studie wurde von der Ethikkommission erstmals in der Sitzung 04-21/22 am 17.01.2022 behandelt.
Die Ethikkommission ist zu folgendem Schluss gekommen:

Es besteht kein Einwand gegen die Durchführung der Studie in der vorliegenden Form.

Stimmberechtigte bzw. anwesende Mitglieder bei der Behandlung waren: Siehe beiliegende Liste vom 17.01.2022.

Kommissionsmitglieder, die für diesen Tagesordnungspunkt als befangen anzusehen waren und daher gemäß Geschäftsordnung an der Entscheidungsfindung und Abstimmung nicht teilgenommen haben: keine

Zur Beurteilung vorliegende Dokumente:

Dokumente eingegangen am 17.12.2021, begutachtet in der Sitzung 04-21/22 am 17.01.2022

✓	Cover Letter 01 Anschreiben 1	17.12.2021
✓	Antragsformular ECS	17.12.2021
	Originalprotokoll 10 SPIOMET4HEALTH_clinical study protocol_final 2.0_04Dec2021_signature BOP Med Uni Graz 2	04.12.2021
	Originalprotokoll 08 SPIOMET4HEALTH_clinical study protocol_final 2.0_04Dec2021_clean 2	04.12.2021
	Originalprotokoll 09 SPIOMET4HEALTH_Protocol Synopsis_final V2.0_04Dec2021_DE_translated 15Dec2021 2	04.12.2021
	Informed Consent Form 15.2 SPIOMET4HEALTH_Webpage_V2.0_DE_translated 26Oct2021 2	26.10.2021
	Informed Consent Form 13.1 SPIOMET4HEALTH_Austrian_PI for Adolescent Participants from 12 to 14 Years_1.0_11Nov2021_DE_translated 07Dec2021 1	07.12.2021
	Informed Consent Form 14.3 SPIOMET4HEALTH Brochure Lifestyle Intervention V2.0, 05Nov2021_DE_translated 07Dec2021 2	07.12.2021

Informed Consent Form 13.3 SPIOMET4HEALTH_Austria_ICF for Adult Participants_v1.0_11Nov2021_DE_translated 07Dec2021 1	07.12.2021
Informed Consent Form 14.10 Patient Engagement Task 5.4-flyer final-210701_MMM&Peggy_DE_translated 15Sep2021 1	15.09.2021
Informed Consent Form 14.5 SPIOMET4HEALTH_Patient Diary Description_final V1.0_29Sep2021_DE_translated 26Oct2021 1	26.10.2021
Informed Consent Form 15.1 SPIOMET4HEALTH Recruitment Campaign Plan V1.0, 27Oct2021_DE_translated 07Dec2021 1	07.12.2021
Informed Consent Form 14.4 Spiomet4health_Storyboard_V1_DE_translated 15Sep2021 1	15.09.2021
Informed Consent Form 13.4 SPIOMET4HEALTH_Austria_ICF for Parents_v1.0_11Nov2021_DE_translated 07Dec2021 1	07.12.2021
Informed Consent Form 13.2 SPIOMET4HEALTH_Austria_ICF for Adolescent Participants from 14 to 17 years_v1.0_11Nov2021_DE_translated 07Dec2021 1	07.12.2021
Informed Consent Form 14.7 SPIOMET4HEALTH_Material-Evaluation-Trial-participants_final_DE_translated 15Sep2021 1	15.09.2021
Informed Consent Form 14.8 SPIOMET4HEALTH_leaflet_DE_translated 15Sep2021 1	15.09.2021
Informed Consent Form 14.2 SCOFF + BEDS questionnaire_DE_V2.0_30Sep2021_translated 15Dec2021 2	15.12.2021
Informed Consent Form 14.1 SPIOMET4HEALTH_Emergency Card_final 1.0_DE_translated 15Sep2021 1	15.09.2021
Informed Consent Form 14.11 ede-q_questionnaire_DE_translated 15Sep2021 6	15.09.2021
Informed Consent Form 14.6 SPIOMET4HEALTH_Patient Diary and Tablet Acceptability_final V1.0_29Sep2021_DE_translated 26Oct2021 1	26.10.2021
Informed Consent Form 14.9 Questions LIP Evaluation_DE_translated 15Sep2021 1	15.09.2021
Conflict of Interest Erklärung 18 Erklarung von Interessenkonflikten EC Graz SPIOMET4HEALTH_signed BOP 07092021 1	07.09.2021
Case Report Form 11.1 SPIOMET4HEALTH_eCRF summary list of variables_v1_04Oct2021 1	04.10.2021
Versicherungsbestätigung Chubb European Group SE ATLSCA05765	03.11.2021
Versicherungsbestätigung Chubb European Group SE ATLSCA05765	29.11.2021
CV 20.1 CV_Elke Froehlich-Reiterer 1	13.10.2020
CV 19.1 CV Obermayer-Pietsch 2020 1	20.04.2020
CV 20.4 GCP_Jasser-Nitsche 1	04.02.2021
CV 20.3 CV_Hildegard Jasser-Nitsche 1	02.05.2021
CV 19.2 GCP_2020_Obermayer-Pietsch 1	10.01.2020
CV 20.2 GCP_Fröhlich-Reiterer 1	04.02.2021
EudraCT Formular (CT1) 04.4 2021-003177-58 AT 20211217 Validation Report 1	17.12.2021
EudraCT Formular (CT1) 04.1 EUDRACT Number 2021-003177-58 1	01.06.2021
EudraCT Formular (CT1) 04.2 2021-003177-58 AT 20211217 CTA PDF Form (1) 1	17.12.2021
Investigator's Brochure 12 SPIOMET-IB_v4_2021_12_16_signed 4	16.12.2021
SUSAR bzw. SAE 11.2 SPIOMET4HEATH_SAE report form_v2.0_02Nov2021 2	02.11.2021
Sonstiges: 16.2 Manual LIP_english_final_1.1_12Aug2021 1.1	12.08.2021
Sonstiges: 22 SPIOMET4HEALTH_Site list_final 1.0_25Oct2021_RM 1	25.10.2021
Sonstiges: 16.3 Documentation Sheets_V1.0_03Aug2021 1	03.08.2021
Sonstiges: 02 Antrag auf Erlass der EK Gebühr 1	17.12.2021
Sonstiges: 05 Scientific Advice EMA 2017 1	18.05.2017
Sonstiges: 03.2 Teil B 1	07.09.2021
Sonstiges: 16.1 SOP Eating Disorder_V1.0_24Aug2021 1	24.08.2021
Sonstiges: 06 002187-PIP01-17 Decision with annexes-1618582984814 1	16.04.2021
Sonstiges: 07 FSJD_SPIOMET4HEALTH_LoA_Optimapharm_final 29Sep2021_clean_signed (1) 1	01.10.2021
Sonstiges: 21 Grant Agreement-899671-SPIOMET4HEALTH 1	28.01.2021

Dokumente eingegangen am 22.12.2021, begutachtet in der Sitzung 04-21/22 am 17.01.2022

✓ Antragsformular ECS Unterschriftenseiten	20.12.2021

Medizinische Universität Graz, Auenbruggerplatz 2, A-8036 Graz. www.medunigraz.at
Rechtsform: Juristische Person öffentlichen Rechts gem. UG 2002. Information: Mitteilungsblatt der Universität. UID: ATU 575 111 79. Bankverbindung: Raiffeisen Landesbank Steiermark IBAN: AT443800000000049510, BIC: RZSTAT2G

Dokumente eingegangen am 15.02.2022 (in der nächsten Begutachtung mitbegutachtet)

✓	Cover Letter 01 SPIOMET4HEALTH_Austria_Cover Letter_EC_Initial Submission - Reply_v1.0_15Feb2022_DE 2	15.02.2022
✓	Cover Letter 01 Anschreiben 1	17.12.2021
✓	Antragsformular ECS 34-267 ex 21/22 inkl. Änderungen	15.02.2022
	Originalprotokoll 09 SPIOMET4HEALTH_Protocol Synopsis_final V2.0_04Dec2021_DE_translated 15Dec2021 2	04.12.2021
	Originalprotokoll 08 SPIOMET4HEALTH_clinical study protocol_final 2.0_04Dec2021_clean 2	04.12.2021
	Originalprotokoll 10 SPIOMET4HEALTH_clinical study protocol_final 2.0_04Dec2021_signature BOP Med Uni Graz 2	04.12.2021
	Informed Consent Form 13.3 SPIOMET4HEALTH_Austria_ICF for Adult Participants_v1.0_11Nov2021_DE_translated 07Dec2021 1	07.12.2021
✓	Informed Consent Form 14.11 ede-q_questionnaire_DE_translated 15Sep2021 6	15.09.2021
	Informed Consent Form 13.2 SPIOMET4HEALTH_Austria_ICF for Adolescent Participants from 14 to 17 years_v1.0_11Nov2021_DE_translated 07Dec2021 1	07.12.2021
✓	Informed Consent Form 14.7 SPIOMET4HEALTH_Material-Evaluation-Trial-participants_final_DE_translated 15Sep2021 1	15.09.2021
✓	Informed Consent Form 05.1 SPIOMET4HEALTH_Patient Diary Description_final 1.0_DE_14Feb2022 Clean 1	14.02.2022
✓	Informed Consent Form 05.2 SPIOMET4HEALTH_Questions LIP Evaluation_v2_14Jan2022_DE_translated 15Feb2022 TC 2	15.02.2022
	Informed Consent Form 04.4 SPIOMET4HEALTH_Austria_ICF for Parents_v2.0_20Jan2022_DE_14Feb2022 Clean 2	14.02.2022
✓	Informed Consent Form 14.3 SPIOMET4HEALTH Brochure Lifestyle Intervention V2.0, 05Nov2021_DE_translated 07Dec2021 2	07.12.2021
✓	Informed Consent Form 05.4 German sf-36v2 standard 2	28.02.2012
✓	Informed Consent Form 05.3 PCOSQ- G with validation 1	16.12.2017
	Informed Consent Form 04.2 SPIOMET4HEALTH_Austria_ICF for Adolescent Participants from 14 to 17 years_v2 26Jan2022_DE 14Feb2022 TC 2	14.02.2022
✓	Informed Consent Form 14.6 SPIOMET4HEALTH_Patient Diary and Tablet Acceptability_final V1.0_29Sep2021_DE_translated 26Oct2021 1	26.10.2021
	Informed Consent Form 04.1 SPIOMET4HEALTH_Austrian_ICF for Adolescent Participants from 12 to 13 years_v2_20Jan2022_DE_14Feb2022 TC 2	14.02.2022
✓	Informed Consent Form 05.2 SPIOMET4HEALTH_Questions LIP Evaluation_v2_14Jan2022_DE_translated 15Feb2022 Clean 2	15.02.2022
✓	Informed Consent Form 13.1 SPIOMET4HEALTH_Austrian_PI for Adolescent Participants from 12 to 14 Years_1.0_11Nov2021_DE_translated 07Dec2021 1	07.12.2021
✓	Informed Consent Form 14.10 Patient Engagement Task 5.4-flyer final-210701_MMM&Peggy_DE_translated 15Sep2021 1	15.09.2021
	Informed Consent Form 13.4 SPIOMET4HEALTH_Austria_ICF for Parents_v1.0_11Nov2021_DE_translated 07Dec2021 1	07.12.2021
✓	Informed Consent Form 14.4 Spiomet4health_Storyboard_V1_DE_translated 15Sep2021 1	15.09.2021
	Informed Consent Form 04.4 SPIOMET4HEALTH_Austria_ICF for Parents_v2.0_20Jan2022_DE_14Feb2022 TC 2	14.02.2022
✓	Informed Consent Form 14.8 SPIOMET4HEALTH_leaflet_DE_translated 15Sep2021 1	15.09.2021
	Informed Consent Form 04.1 SPIOMET4HEALTH_Austrian_ICF for Adolescent Participants from 12 to 13 years_v2_20Jan2022_DE_14Feb2022 Clean 2	14.02.2022
✓	Informed Consent Form 05.6 SPIOMET4HEALTH_pregnancy test tracker_final 1.0_20Jan2022_translated to GER on 14Feb2022 1	14.02.2022
	Informed Consent Form 04.2 SPIOMET4HEALTH_Austria_ICF for Adolescent Participants from 14 to 17 years_v2 26Jan2022_DE 14Feb2022 Clean 2	14.02.2022
✓	Informed Consent Form 15.1 SPIOMET4HEALTH Recruitment Campaign Plan V1.0, 27Oct2021_DE_translated 07Dec2021 1	07.12.2021
✓	Informed Consent Form 14.1 SPIOMET4HEALTH_Emergency Card_final 1.0_DE_translated 15Sep2021 1	15.09.2021
✓	Informed Consent Form 14.2 SCOFF + BEDS questionnaire_DE_V2.0_30Sep2021_translated 15Dec2021 2	15.12.2021
✓	Informed Consent Form 05.1 SPIOMET4HEALTH_Patient Diary Description_final 1.0_DE_14Feb2022 TC 1	14.02.2022

Medizinische Universität Graz, Auenbruggerplatz 2, A-8036 Graz. www.medunigraz.at

Rechtsform: Juristische Person öffentlichen Rechts gem. UG 2002. Information: Mitteilungsblatt der Universität. UID: ATU 575 111 79. Bankverbindung: Raiffeisen Landesbank Steiermark IBAN: AT443800000000049510, BIC: RZSTAT2G

	Informed Consent Form 04.3 SPIOMET4HEALTH_Austria_ICF for Adult Participants_v2.0_20Jan2022_DE_14Feb2022 Clean 2	14.02.2022
	Informed Consent Form 04.3 SPIOMET4HEALTH_Austria_ICF for Adult Participants_v2.0_20Jan2022_DE_14Feb2022 TC 2	14.02.2022
✓	Informed Consent Form 14.9 Questions LIP Evaluation_DE_translated 15Sep2021 1	15.09.2021
✓	Informed Consent Form 15.2 SPIOMET4HEALTH_Webpage_V2.0_DE_translated 26Oct2021 2	26.10.2021
✓	Informed Consent Form 05.5 German SF-36v2 Interview Script Standard 2	10.09.2012
✓	Informed Consent Form 14.5 SPIOMET4HEALTH_Patient Diary Description_final V1.0_29Sep2021_DE_translated 26Oct2021 1	26.10.2021
✓	Conflict of Interest Erklärung 18 Erklarung von Interessenkonflikten EC Graz SPIOMET4HEALTH_signed BOP 07092021 1	07.09.2021
✓	Case Report Form 11.1 SPIOMET4HEALTH_eCRF summary list of variables_v1_04Oct2021 1	04.10.2021
✓	Versicherungsbestätigung Chubb European Group SE ATLSCA05765	29.11.2021
✓	Versicherungsbestätigung Chubb European Group SE ATLSCA05765	03.11.2021
	Versicherungsbestätigung Chubb European Group SE ATLSCA05765	29.12.2021
✓	Versicherungsbestätigung Chubb European Group SE ATLSCA05765	03.11.2021
✓	CV 19.1 CV Obermayer-Pietsch 2020 1	20.04.2020
✓	CV 19.2 GCP_2020_Obermayer-Pietsch 1	10.01.2020
✓	CV 20.2 GCP_Fröhlich-Reiterer 1	04.02.2021
✓	CV 08.2 CV_Gumpold_2022 1	10.01.2022
✓	CV 08.1 CV_Cornelia_Missbrenner_2022 1	26.01.2022
✓	CV 20.1 CV_Elke Froehlich-Reiterer 1	13.10.2020
✓	CV 08.3 CV_Tandl_signed 1	27.01.2022
✓	CV 20.4 GCP_Jasser-Nitsche 1	04.02.2021
✓	CV 20.3 CV_Hildegard Jasser-Nitsche 1	02.05.2021
✓	EudraCT Formular (CT1) 04.2 2021-003177-58 AT 20211217 CTA PDF Form (1) 1	17.12.2021
✓	EudraCT Formular (CT1) 04.1 EUDRACT Number 2021-003177-58 1	01.06.2021
✓	EudraCT Formular (CT1) 04.4 2021-003177-58 AT 20211217 Validation Report 1	17.12.2021
✓	Investigator's Brochure 12 SPIOMET-IB_v4_2021_12_16_signed 4	16.12.2021
✓	SUSAR bzw. SAE 11.2 SPIOMET4HEATH_SAE report form_v2.0_02Nov2021 2	02.11.2021
✓	Sonstiges: 05 Scientific Advice EMA 2017 1	18.05.2017
✓	Sonstiges: 07 FSJD_SPIOMET4HEALTH_LoA_Optimapharm_final 29Sep2021_clean_signed (1) 1	01.10.2021
✓	Sonstiges: 02 Antrag auf Erlass der EK Gebühr 1	17.12.2021
✓	Sonstiges: 16.1 SOP Eating Disorder_V1.0_24Aug2021 1	24.08.2021
✓	Sonstiges: 06 002187-PIP01-17 Decision with annexes-1618582984814 1	16.04.2021
✓	Sonstiges: 21 Grant Agreement-899671-SPIOMET4HEALTH 1	28.01.2021
✓	Sonstiges: 16.2 Manual LIP_english_final_1.1_12Aug2021 1.1	12.08.2021
✓	Sonstiges: 03.2 Teil B 1	07.09.2021
✓	Sonstiges: 16.3 Documentation Sheets_V1.0_03Aug2021 1	03.08.2021
✓	Sonstiges: 22 SPIOMET4HEALTH_Site list_final 1.0_25Oct2021_RM 1	25.10.2021
✓	Sonstiges: 03 SPIOMET4HEALTH_Statement on financial aspect of the study_final 1.0_14Feb2022 1	14.02.2022

Dokumente eingegangen am 11.03.2022 (in der nächsten Begutachtung mitbegutachtet)

✓	Cover Letter	15.02.2022
	Informed Consent Form Kinder (12-13 Jahre) 2.0	14.02.2022
	Informed Consent Form Jugendliche (14-17 Jahre) 2.0	14.02.2022
	Informed Consent Form 2.0	14.02.2022
	Informed Consent Form Eltern 2.0	14.02.2022
✓	Fragebögen SF-36v2 Script 2012	
✓	Fragebögen LIP 2.0	15.02.2022
✓	Fragebögen SF-36v2 2012	
✓	Fragebögen PCOSQ- G undatiert	
✓	Zahlungsbeleg	01.02.2022
✓	Versicherungsbestätigung Chubb -Personenschaden mit Haftpflicht, Rechtsschutz und Bedingungen ATLSCA05765	03.11.2021
	Versicherungsbestätigung Chubb ATLSCA05765	29.12.2021
✓	CV Mitarbeiter Missbrenner	26.01.2022
✓	CV Mitarbeiter Gumpold	10.01.2022
✓	CV Mitarbeiter Tandl	27.01.2022
✓	Patienten-/Probandentagebuch 1.0	14.02.2022
✓	Sonstiges: Pregnancy Test Tracker 1.0	14.02.2022
✓	Sonstiges: GP Letter 2.0	14.12.2021
✓	Sonstiges: Stellungnahme zur Bearbeitungsmitteilung	14.02.2022

Dokumente eingegangen am 20.04.2022, begutachtet im 'expedited Review' am 12.05.2022

✓	Cover Letter	20.04.2022
✓	Originalprotokoll 3.0	16.03.2022
✓	Informed Consent Form Eltern 3.0	19.04.2022
✓	Informed Consent Form 3.0	19.04.2022
✓	Informed Consent Form Jungedliche (14-17 Jahre) 3.0	19.04.2022
✓	Informed Consent Form Kinder (12-13 Jahre) 3.0	19.04.2022
✓	Versicherungsbestätigung Chubb ATLSCA05765	11.04.2022
✓	Versicherungsbestätigung Chubb ATLSCA05765	03.11.2021
✓	Patienten-/Probandentagebuch 2.0	12.04.2022
✓	Sonstiges: mit Stellungnahme zur Bearbeitungsmitteilung (Biometrie)	20.04.2022
✓	Sonstiges: mit Stellungnahme zur Bearbeitungsmitteilung (Glukose)	20.04.2022
✓	Protocol Signature Page Obermayer-Pietsch	15.04.2022

Die Ethikkommission geht – rechtlich unverbindlich – davon aus, dass es sich um eine klinische Prüfung nach AMG handelt und macht darauf aufmerksam, dass vor Beginn der Prüfung ein ordnungsgemäßer Antrag auf Genehmigung an das Bundesamt für Sicherheit im Gesundheitswesen zu stellen ist.

Das Votum der Ethikkommission berührt in keiner Weise die alleinige Verantwortung der Prüferin / des Prüfers / der Prüfer für die ordnungsgemäße Durchführung der Studie unter Einhaltung aller einschlägiger gesetzlicher Bestimmungen und Richtlinien.

Weiters machen wir darauf aufmerksam, dass der Kommission unverzüglich zu melden sind:

- Abweichungen vom Protokoll aus Sicherheitsgründen oder Protokolländerungen

- Änderungen, die das Risiko der Teilnehmer/-innen erhöhen oder die Durchführung der Studie wesentlich beeinflussen

- Mutmaßliche unerwartete schwerwiegende Nebenwirkungen - SUSARs (AMG-Studien ab 1.5.2004) oder schwerwiegende unerwünschte Ereignisse - SAEs (andere Studien)

- Jegliche Information über sonstige Umstände, die die Sicherheit der Teilnehmer/-innen oder die Durchführung der Studie beeinträchtigen können

Begründung: Es handelt sich um eine relevante Fragestellung, die mit geeigneter Methodik

beantwortet werden soll. Die vom Antragsteller vorgenommene Bewertung des Nutzen/Risiko-Verhältnisses ist plausibel.

Dieses Votum gilt für ein Jahr ab dem Datum der Ausstellung. Bei längerer Studiendauer ist rechtzeitig vor Ablauf der Gültigkeit des Votums ein Zwischenbericht vorzulegen (Berichtsformular), um eine etwaige Verlängerung zu erlangen.

Graz, 12. Mai 2022

Univ.Prof.DI Dr.Josef Haas<br>Vorsitzender	Univ.Prof.Dr.Hans Peter Dimai<br>Stv. Vorsitzender

Achtung: Bitte bei allen das Projekt betreffende Schreiben oder telefonischen Anfragen die EK-Nummer angeben!

Medizinische Universität Graz, Auenbruggerplatz 2, A-8036 Graz. www.medunigraz.at
Rechtsform: Juristische Person öffentlichen Rechts gem. UG 2002. Information: Mitteilungsblatt der Universität. UID: ATU 575 111 79. Bankverbindung: Raiffeisen Landesbank Steiermark IBAN: AT443800000000049510, BIC: RZSTAT2G

Medizinische Universität Graz
Ethikkommission

Auenbruggerplatz 2, A-8036 Graz
ethikkommission@medunigraz.at
Tel.: +43 / 316 / 385-13928, Fax: -14348

Liste der stimmberechtigten bzw. anwesenden Mitglieder

am 17. Jänner 2022

Univ.Prof.DI Dr.Andrea Berghold
Univ.Prof.Dr. Hans P. Dimai
Univ.Prof.Dr.Thomas Griesbacher
Univ.Prof.DI Dr.Josef Haas
PD.Dr.Christian Fazekas
Dr. Aitak Farzi
Univ.Prof.Dr.Elisabeth Mahla
Univ.Prof.Dr.Friedrich Reiterer
DI Dr.Regina Riedl
Univ.Prof.Dr.Rudolf Stauber
Univ.Prof.Dr.Hermann Toplak
Elisabeth Trummer, BA
Ursula Vennemann
MMag.a Anna Weiß
Ing.Franz Deutschmann
Univ.Prof.Dr. Leopold Neuhold
Univ.Prof.Dr.Peter J. Schick
Univ.Prof.Dr. Michael Speicher
Ass.-Prof.Dr.jur. Armin-Bernhard Stolz
Univ.Prof.Dr.Kurt Weber
Univ.Prof.Dr.Andreas Zimmer

Beigezogene Fachärzte

Prof. PD DMBalic
PD DDr.Susanne Bengesser
Assoz. Prof. PD Dr.Jörg Lindenmann
Ao.Univ.-Prof. Dr.med.univ.Rainer Lipp
Univ.Prof.Dr.Wolfgang Schwinger
Assoz.Prof. PD Dr.Georg Singer
Univ.Prof.Dr.Peter Wolf

Medizinische Universität Graz, Auenbruggerplatz 2, A-8036 Graz. www.medunigraz.at
Rechtsform: Juristische Person öffentlichen Rechts gem. UG 2002. Information: Mitteilungsblatt der Universität. UID: ATU 575 111 79. Bankverbindung: Raiffeisen Landesbank Steiermark IBAN: AT443800000000049510, BIC: RZSTAT2G

CERTIFICATION OF TRANSLATION

Quote number: 2205-06332

Source document: ek0000421991.pdf

Full Title of Documents: ek0000421991_ translated into English on 2June2022.docx

Footer: ek0000421991_translated into English 2June2022.docx

Original Source Language of Document: German
Language Document is to be Translated into: English

Declaration of Translator:

I, Jan Hirš on behalf of Skrivanek s. r. o., hereby certify that the translation into English agrees with the text of the original document in German language, that there were no changes/corrections made to the original document neither the translation, and that it was translated by a certified translator fluent in both above mentioned languages.

I, Jan Hirš on behalf of Skrivanek s. r. o., also confirm that the above mentioned service has been rendered and that the translated document is to the best of my knowledge a true and faithful translation of the original document as provided by the client

With Best regards,

Medizinische Universität Graz
Ethikkommission

Auenbruggerplatz 2, A-8036 Graz
ethikkommission@medunigraz.at
Phone: +43 / 316 / 385-13928, Fax: -14348

VOTE

valid until 12/05/2023

EC number: 34-169 ex 21/22 **EudraCT no:** 2021-003177-58
1608-2021

Study title: A Phase II, randomised, multi-centric, multi-national clinical trial to evaluate the efficacy, tolerability, and safety of a fixed dose combination of Spironolactone, Pioglitazone & Metformin (SPIOMET) for adolescent girls and young adult women (AYAs) with polycystic ovary syndrome (PCOS).

Examiner: Professor Dr med. Barbara Obermayer-Pietsch
MU Graz. University Clinic for Internal Medicine, Dept. of Endocrinology and Diabetology

Sponsor: Fundació Sant Joan de Déu
Contact person: Prof. Lourdes Ibáñez, 08950 Esplugues de Llobregat, C/ Santa Rosa, 39-57, 4a planta

CRO: Optimapharm d.o.o.
Contact person: MPharm Vladimir Vujovic, 10000 Zagreb, Ulica grada Vukovara 284

Applicant: Optimapharm d.o.o.
Contact person: MPharm Vladimir Vujovic, 10000 Zagreb, Ulica grada Vukovara 284

The above study was first discussed by the Ethics Committee at the meeting 04-21/22 on 17/01/2022.
The Ethics Committee has come to the following conclusion:

There is no objection to conducting the study in its present form.

Members entitled to vote or who were present during the discussion were: see attached list of 17/01/2022.

Committee members who were considered biased for this agenda item and therefore, in accordance with the procedure, were excluded from the decision-making and voting: none.

Documents available for evaluation:

Documents received on 17/12/2021, reviewed at the meeting 04-21/22 on 17/01/2022.

✓ Cover Letter 01 Anschreiben 1	17/12/2021
✓ ECS application form	17/12/2021
Original protocol 10 SPIOMET4HEALTH_clinical study protocol_final 2.0_04Dec2021_signature BOP Med Uni Graz 2	04/12/2021
Original protocol 08 SPIOMET4HEALTH_clinical study protocol_final 2.0_04Dec2021_clean 2	04/12/2021
Original protocol 09 SPIOMET4HEALTH_Protocol Synopsis_final V2.0_04Dec2021_DE_translated 15Dec2021 2	04/12/2021
Informed Consent Form 15.2 SPIOMET4HEALTH_Webpage_V2.0_DE_translated 26Oct2021 2	26/10/2021
Informed Consent Form 13.1 SPIOMET4HEALTH_Austrian_PI for Adolescent Participants from 12 to 14 YearsJ .0_11 Nov2021_DE_translated 07Dec2021 1	07/12/2021
Informed Consent Form 14.3 SPIOMET4HEALTH Brochure Lifestyle Intervention V2.0, 05Nov2021_DE_translated 07Dec2021 2	07/12/2021

Medizinische Universität Graz, Auenbruggerplatz 2, A-8036 Graz, www.medunigraz.at
Legal form: Legal person under public law in accordance with the Universities Act 2002 Information: University bulletin VAT ID no: ATU 575 111 79 Bank details: Raiffeisen-
Landesbank Steiermark IBAN: AT443800000000049510, BIC: RZSTAT2G
ek0000421991_translated into English 2June2022.docx

Informed Consent Form 13.3 SPIOMET4HEALTH_Austria_ICF for Adult Participants_v1.0_11Nov2021_DE_translated 07Dec2021 1	07/12/2021
Informed Consent Form 14.10 Patient Engagement Task 5.4-flyer final-210701_MMM&Peggy_DE_translated 15Sep2021 1	15/09/2021
Informed Consent Form 14.5 SPIOMET4HEALTH_Patient Diary Descriptionjinal V1.0_29Sep2021_DE_translated 26Oct2021 1	26/10/2021
Informed Consent Form 15.1 SPIOMET4HEALTH Recruitment Campaign Plan V1.0, 27Oct2021_DE_translated 07Dec2021 1	07/12/2021
Informed Consent Form 14.4 Spiomet4health_Storyboard_V1_DE_translated 15Sep2021 1	15/09/2021
Informed Consent Form 13.4 SPIOMET4HEALTH_Austria_ICF for Parents_v1.0_11Nov2021_DE_translated 07Dec2021 1	07/12/2021
Informed Consent Form 13.2 SPIOMET4HEALTH_Austria_ICF for Adolescent Participants from 14to 17years_v1.0_11Nov2021_DE_translated 07Dec2021 1	07/12/2021
Informed Consent Form 14.7 SPIOMET4HEALTH_Material-Evaluation-Trial-participants_final_DE_translated 15Sep2021 1	15/09/2021
Informed Consent Form 14.8 SPIOMET4HEALTH_leaflet_DE_translated 15Sep2021 1	15/09/2021
Informed Consent Form 14.2 SCOFF + BEDS questionnaire_DE_V2.0_30Sep2021_translated 15Dec2021 2	15/12/2021
Informed Consent Form 14.1 SPIOMET4HEALTH_Emergency Cardjinal 1.0_DE_translated 15Sep2021 1	15/09/2021
Informed Consent Form 14.11 ede-q_questionnaire_DE_translated 15Sep2021 6	15/09/2021
Informed Consent Form 14.6 SPIOMET4HEALTH_Patient Diary and Tablet Acceptabilityjinal V1.0_29Sep2021_DE_translated 26Oct2021 1	26/10/2021
Informed Consent Form 14.9 Questions LIP Evaluation_DE_translated 15Sep2021 1	15/09/2021
Conflict of Interest Statement 18 Erklärung von Interessenkonflikten EC Graz SPIOMET4HEALTH_signed BOP 07092021 1	07/09/2021
Case Report Form 11.1 SPIOMET4HEALTH_eCRF summary list of variables_v1_04Oct2021 1	04/10/2021
Confirmation of insurance Chubb European Group SE ATLSCA05765	03/11/2021
Versicherungsbestätigung Chubb European Group SE ATLSCA05765	29/11/2021
CV 20.1 CV_Elke Froehlich-Reiterer 1	13/10/2020
CV 19.1 CV Obermayer-Pietsch 2020 1	20/04/2020
CV 20.4 GCP_Jasser-Nitsche 1	04/02/2021
CV 20.3 CVJHildegard Jasser-Nitsche 1	02/05/2021
CV 19.2 GCP_2020_Obermayer-Pietsch 1	10/01/2020
CV 20.2 GCP_Fröhlich-Reiterer 1	04/02/2021
EudraCT Formular (CT1) 04.4 2021-003177-58 AT 20211217 Validation Report 1	17/12/2021
EudraCT Formular (CT1) 04.1 EUDRACT Number 2021-003177-58 1	01/06/2021
EudraCT Form (CT1) 04.2 2021-003177-58 -20211217 AT 1 CTA PDF Form (1)	17/12/2021
Investigator's Brochure 12 SPIOMET-IB_v4_2021_12_16_signed 4	16/12/2021
SUSAR resp. SAE 11.2 SPIOMET4HEATH_SAE report form_v2.0_02Nov2021 2	02/11/2021
Other: 16.2 Manual LIP_english_finaM-1_12Aug2021 1.1	12/08/2021
Other: 22 SPIOMET4HEALTH_Site listjinal 1.0_25Oct2021_RM 1	25/10/2021
Other: 16.3 Documentation Sheets_V1.0_03Aug2021 1	03/08/2021
Other: 02 Application for waiver of the EC fee 1	17/12/2021
Other: 05 Scientific Advice EMA 2017 1	18/05/2017
Other: 03.2 Teil B 1	07/09/2021
Other: 16.1 SOP Eating Disorder_V1.0_24Aug2021 1	24/08/2021
Other: 06 002187-PIP01-17 Decision with annexes-1618582984814 1	16/04/2021
Other: 07 FSJD_SPIOMET4HEALTH_LoA_Optimapharm_final 29Sep2021_clean_signed (1) 1	01/10/2021
Other: 21 Grant Agreement-899671-SPIOMET4HEALTH 1	28/01/2021

Documents received on 22/12/2021, reviewed at the meeting 04-21/22 on 17/01/2022

✓ ECS Application form signature pages	20/12/2021

Medizinische Universität Graz, Auenbruggerplatz 2, A-8036 Graz, www.medunigraz.at
Legal form: Legal person under public law in accordance with the Universities Act 2002 Information: University bulletin VAT ID no: ATU 575 111 79 Bank details: Raiffeisen-
Landesbank Steiermark IBAN: AT443800000000049510, BIC: RZSTAT2G
ek0000421991_translated into English 2June2022.docx

Documents received on 15/02/2022 (assessment included in the next review)

- ✓ Cover Letter 01 SPIOMET4HEALTH_Austria_Cover Letter_ECJnitial Submission - 15/02/2022 Reply_v1.0_15Feb2022_DE 2
- ✓ Cover Letter 01 Anschreiben 1 17/12/2021
- ✓ Application form ECS 34-267 ex 21/22 incl. amendments 15/02/2022

	Original protocol 09 SPIOMET4HEALTH_Protocol Synopsis_final V2.0_04Dec2021 JDEJranslated 15Dec2021 2	04/12/2021
	Original protocol 08 SPIOMET4HEALTH_clinical study protocoLfinal 2.0_04Dec2021_clean 2	04/12/2021
	Original protocol 10 SPIOMET4HEALTH_clinical study protocol_final 2.0_04Dec2021_signature BOP Med Uni Graz 2	04/12/2021
	Informed Consent Form 13.3 SPIOMET4HEALTH_Austria_ICF for Adult Participants_v1.0_11Nov2021_DE_translated 07Dec2021 1	07/12/2021
✓	Informed Consent Form 14.11 ede-q_questionnaire_DE_translated 15Sep2021 6	15/09/2021
	Informed Consent Form 13.2 SPIOMET4HEALTH_Austria_ICF for Adolescent Participants from 14 to 17 years_v1.0_11 Nov2021_DE_translated 07Dec2021 1	07/12/2021
✓	Informed Consent Form 14.7 SPIOMET4HEALTH_Material-Evaluation-Trial-participants_final_DE_translated 15Sep2021 1	15/09/2021
	Informed Consent Form 05.1 SPIOMET4HEALTH_Patient Diary Description_final 1.0_DE_14Feb2022 Clean 1	14/02/2022
✓	Informed Consent Form 05.2 SPIOMET4HEALTH_Questions LIP Evaluation_v2_14Jan2022_DE_translated 15Feb2022 TC 2	15/02/2022
	Informed Consent Form 04.4 SPIOMET4HEALTH_Austria_ICF for Parents_v2.0_20Jan2022_DE_14Feb2022 Clean 2	14/02/2022
✓	Informed Consent Form 14.3 SPIOMET4HEALTH Brochure Lifestyle Intervention V2.0, 05Nov2021_DE_translated 07Dec2021 2	07/12/2021
✓	Informed Consent Form 05.4 German sf-36v2 Standard 2	28/02/2012
✓	Informed Consent Form 05.3 PCOSQ-G with validation 1	16/12/2017
	Informed Consent Form 04.2 SPIOMET4HEALTH_Austria_ICF for Adolescent Participants from 14 to 17 years_v2 26Jan2022_DE 14Feb2022 TC 2	14/02/2022
✓	Informed Consent Form 14.6 SPIOMET4HEALTH_Patient Diary and Tablet Acceptabilityjinal V1.0_29Sep2021_DEJranslated 26Oct2021 1	26/10/2021
	Informed Consent Form 04.1 SPIOMET4HEALTH_Austrian_ICF for Adolescent Participants from 12 to 13 years_v2_20Jan2022_DE_14Feb2022 TC 2	14/02/2022
✓	Informed Consent Form 05.2 SPIOMET4HEALTH_Questions LIP Evaluation_v2_14Jan2022_DE_translated 15Feb2022 Clean 2	15/02/2022
✓	Informed Consent Form 13.1 SPIOMET4HEALTH_Austrian_PI for Adolescent Participants from 12to 14 Years_1.0_11Nov2021_DE_translated 07Dec2021 1	07/12/2021
✓	Informed Consent Form 14.10 Patient Engagement Task 5.4-flyer final-210701_MMM&Peggy_DE_translated 15Sep2021 1	15/09/2021
	Informed Consent Form 13.4 SPIOMET4HEALTH_Austria_ICF for Parents_v1.0_11Nov2021JDE_translated 07Dec2021 1	07/12/2021
✓	Informed Consent Form 14.4Spiomet4health_Storyboard_V1_DE_translated 15Sep2021 1	15/09/2021
	Informed Consent Form 04.4 SPIOMET4HEALTH_Austria_ICF for Parents__v2.0_20Jan2022_DE_14Feb2022 TC 2	14/02/2022
✓	Informed Consent Form 14.8 SPIOMET4HEALTH_leaflet_DE_translated 15Sep2021 1	15/09/2021
	Informed Consent Form 04.1 SPIOMET4HEALTH_Austrian_ICF for Adolescent Participants from 12 to 13 years_v2_20Jan2022_DE_14Feb2022 Clean 2	14/02/2022
✓	Informed Consent Form 05.6 SPIOMET4HEALTH_pregnancy test trackerjinal 1.0_20Jan2022Jranslated to GER on 14Feb2022 1	14/02/2022
	Informed Consent Form 04.2 SPIOMET4HEALTH_Austria_ICF for Adolescent Participants from 14to 17 years_v2 26Jan2022_DE 14Feb2022 Clean 2	14/02/2022
✓	Informed Consent Form 15.1 SPIOMET4HEALTH Recruitment Campaign Plan V1.0, 27Oct2021_DE_translated 07Dec2021 1	07/12/2021
✓	Informed Consent Form 14.1 SPIOMET4HEALTH_Emergency Cardjinal 1.0_DE_translated15Sep2021 1	15/09/2021
✓	Informed Consent Form 14.2 SCOFF + BEDS questionnaire_DE_V2.0_30Sep2021_translated 15Dec2021 2	15/12/2021
✓	Informed Consent Form 05.1 SPIOMET4HEALTH_Patient Diary Descriptionjinal 1.0 DE 14Feb2022TC1	14/02/2022

Medizinische Universität Graz, Auenbruggerplatz 2, A-8036 Graz, www.medunigraz.at

Legal form: Legal person under public law in accordance with the Universities Act 2002 Information: University bulletin VAT ID no: ATU 575 111 79 Bank details: Raiffeisen-
Landesbank Steiermark IBAN: AT443800000000049510, BIC: RZSTAT2G

ek0000421991_translated into English 2June2022.docx

✓	Informed Consent Form 04.3 SPIOMET4HEALTH„Austria_ICFfor Adult Participants_v2.0_20Jan2022_DE_14Feb2022 Clean 2	14/02/2022
	Informed Consent Form 04.3 SPIOMET4HEALTH_Austria_ICF for Adult Participants_v2.0_20Jan2022_DEJ4Feb2022 TC 2	14/02/2022
✓	Informed Consent Form 14.9 Questions LIP Evaluation_DE_translated 15Sep2021 1	15/09/2021
✓	Informed Consent Form 15.2 SPIOMET4HEALTH_Webpage_V2.0_DE_translated 26Oct2021 2	26/10/2021
✓	•Informed Consent Form 05.5 German SF-36v2 Interview Script Standard 2	10/09/2012
✓	Informed Consent Form 14.5 SPIOMET4HEALTH_Patient Diary Descriptionjinal V1.0_29Sep2021_DE_translated 26Oct2021 1	26/10/2021
✓	Conflict of Interest Statement 18 Erklärung von Interessenkonflikten EC Graz SPIOMET4HEALTH_signed BOP 07092021 1	07/09/2021
✓	Case Report Form 11.1 SPIOMET4HEALTH_eCRF summary list of variables_v1_04Oct2021 1	04/10/2021
✓	Versicherungsbestätigung Chubb European Group SE ATLSCA05765	29/11/2021
✓	Confirmation of insurance Chubb European Group SE ATLSCA05765	03/11/2021
	Confirmation of insurance Chubb European Group SE ATLSCA05765	29/12/2021
✓	Confirmation of insurance Chubb European Group SE ATLSCA05765	03/11/2021
✓	CV 19.1 CV Obermayer-Pietsch 2020 1	20/04/2020
✓	CV19.2GCP_2020_Obermayer-Pietsch 1	10/01/2020
✓	CV 20.2 GCP_Fröhlich-Reiterer 1	04/02/2021
✓	CV 08.2 CV_Gumpold_2022 1	10/01/2022
✓	CV 08.1 CV_CorneliaJv1issbrenner_2022 1	26/01/2022
✓	CV 20.1 CV_Elke Froehlich-Reiterer 1	13/10/2020
✓	CV 08.3 CV_Tandl_signed 1	27/01/2022
✓	CV 20.4 GCP_Jasser-Nitsche 1	04/02/2021
✓	CV 20.3 CV_Hildegard Jasser-Nitsche 1	02/05/2021
✓	EudraCT Formular (CT1) 04.2 2021-003177-58 AT 20211217 CTA PDF Form (1) 1	17/12/2021
✓	EudraCT Formular (CT1) 04.1 EUDRACT Number 2021 -003177-58 1	01/06/2021
✓	EudraCT Formular (CT1) 04.4 2021-003177-58 AT 20211217 Validation Report 1	17/12/2021
✓	Investigator's Brochure 12 SPIOMET-IB_v4_2021_12_16_signed 4	16/12/2021
✓	SUSAR resp. SAE 11.2 SPIOMET4HEATH_SAE report form_v2.0_02Nov2021 2	02/11/2021
✓	Other: 05 Scientific Advice EMA 2017 1	18/05/2017
✓	Other: 07 FSJD_SPIOMET4HEALTH_LoA_Optimapharm_final 29Sep2021_clean_signed (1) 1	01/10/2021
✓	Other: 02 Application for waiver of the EC fee 1	17/12/2021
✓	Other: 16.1 SOP Eating Disorder_V1.0_24Aug2021 1	24/08/2021
✓	Other: 06 002187-PIP01-17 Decision with annexes-1618582984814 1	16/04/2021
✓	Other: 21 Grant Agreement-899671-SPIOMET4HEALTH 1	28/01/2021
✓	Other: 16.2 Manual LIP_english_finaM.1_12Aug2021 1.1	12/08/2021
✓	Other: 03.2 Teil B 1	07/09/2021
✓	Other: 16.3 Documentation Sheets_V1.0_03Aug2021 1	03/08/2021
✓	Other: 22 SPIOMET4HEALTH_Site list_final 1.0_25Oct2021_RM 1	25/10/2021
✓	Other: 03 SPIOMET4HEALTH_Statement on financial aspect of the studyjinal 1.0 14Feb2022 1	14/02/2022

Medizinische Universität Graz, Auenbruggerplatz 2, A-8036 Graz, www.medunigraz.at

Legal form: Legal person under public law in accordance with the Universities Act 2002 Information: University bulletin VAT ID no: ATU 575 111 79 Bank details: Raiffeisen-
Landesbank Steiermark IBAN: AT443800000000049510, BIC: RZSTAT2G
ek0000421991_translated into English 2June2022.docx

Documents received on 11/03/2022 (assessment included in the next review)

✓ Cover Letter	15/02/2022
Informed Consent Form Children (12-13 years) 2.0	14/02/2022
Informed Consent Form Youth (14-17 years) 2.0	14/02/2022
Informed Consent Form 2.0	14/02/2022
Informed Consent Form Parents 2.0	14/02/2022
✓ Questionnaires SF-36v2 Script 2012	
✓ Questionnaires LIP 2.0	15/02/2022
✓ Questionnaires SF-36v2 2012	
✓ Questionnaires PCOSQ- G undated	
✓ Payment receipt	01/02/2022
✓ Confirmation of insurance Chubb -Personal injury with liability, legal protection 03/11/2021 and conditions ATLSCA05765	
✓ Confirmation of insurance Chubb ATLSCA05765	29/12/2021
✓ CV Staff member Missbrenner	26/01/2022
✓ CV Staff member Gumpold	10/01/2022
✓ CV Staff member Tandl	27/01/2022
✓ Patient/proband diary 1.0 14/02/2022	
✓ Other: Pregnancy Test Tracker 1.0	14/02/2022
✓ Other: GP Letter 2.0	14/12/2021
✓ Other: Statement on the processing notification 14/02/2022	

Documents received on 20/04/2022, reviewed at the 'expedited Review' meeting on 12/05/2022

✓ Cover Letter	20/04/2022
✓ Original protocol 3.0 16/03/2022	
✓ Informed Consent Form Parents 3.0 19/04/2022	
✓ Informed Consent Form 3.0	19/04/2022
✓ Informed Consent Form Youth (14-17 years) 3.0	19/04/2022
✓ Informed Consent Form Children (12-13 years) 3.0	19/04/2022
✓ Confirmation of insurance Chubb ATLSCA05765	11/04/2022
✓ Confirmation of insurance Chubb ATLSCA05765	03/11/2021
✓ Patient/proband diary 2.0	12/04/2022
✓ Other: with statement on the processing notification (biometrics)	20/04/2022
✓ Other: with statement on the processing notification (biometrics) 20/04/2022	
✓ Protocol Signatuře Page Obermayer-Pietsch	15/04/2022

The Ethics Committee assumes – without any legal obligation – that this is a clinical trial according to the AMG and stresses that a proper request for authorisation must be submitted to the Austrian Federal Office for Safety in Health Care before the trial begins.

The vote of the Ethics Committee in no way affects the sole responsibility of the examiner(s) for the proper conduct of the study in compliance with all relevant legal provisions and guidelines.

Furthermore, we draw your attention to the fact that the Commission must be notified immediately about:

- Deviations from the protocol for security reasons or protocol amendments

- Changes that increase the risk to the participants or significantly affect the conduct of the study

- Suspected unexpected serious adverse events - SUSARs (AMG studies as of 01/05/2004) or serious adverse events - SAEs (other studies)

- Any information about other circumstances that may affect the safety of the participants or the conduct of the study

Rationale: This is a relevant question that should be answered using appropriate methodology. The applicant's assessment of the benefit/risk ratio is plausible.

Medizinische Universität Graz, Auenbruggerplatz 2, A-8036 Graz, www.medunigraz.at
Legal form: Legal person under public law in accordance with the Universities Act 2002 Information: University bulletin VAT ID no: ATU 575 111 79 Bank details: Raiffeisen-
Landesbank Steiermark IBAN: AT443800000000049510, BIC: RZSTAT2G

This vote is valid for one year from the date of issue. In the case of a longer duration of the study, an interim report must be submitted in good time before the expiry of the validity of the vote (report form) in order to obtain a possible extension.

Graz, 12 May 2022

Univ. Prof.i.R. Dr Josef Haas<br>Chairman	Univ. Prof. Dr Hans Peter Dimai<br>Dep. Chairman

Note: Please quote the EC number in all letters or phone enquiries concerning the project!

Medizinische Universität Graz, Auenbruggerplatz 2, A-8036 Graz, www.medunigraz.at

Legal form: Legal person under public law in accordance with the Universities Act 2002 Information: University bulletin VAT ID no: ATU 575 111 79 Bank details: Raiffeisen-
Landesbank Steiermark IBAN: AT443800000000049510, BIC: RZSTAT2G

ek0000421991_translated into English 2June2022.docx

Medizinische Universität Graz
Ethics Committee

Auenbruggerplatz 2, A-8036 Graz
ethikkommission@medunigraz.at
Phone: +43 / 316 / 385-13928, Fax: -14348

List of members entitled to vote or be present

on 17 January 2022

Univ. Prof. DI Dr Andrea Berghold
Univ. Prof. Dr Hans P. Dimai
Univ. Prof. Dr Thomas Griesbacher
Univ. Prof. DI Dr Josef Haas
PD. Dr Christian Fazekas
Dr Aitak Farzi
Univ. Prof. Dr Elisabeth Mahla
Univ. Prof. Dr Friedrich Reiterer
DI Dr Regina Riedl
Univ. Prof. Dr Rudolf Stauber
Univ. Prof. Dr Hermann Toplak
Elisabeth Trümmer, BA
Ursula Vennemann
MMag.a Anna Weiss
Ing. Franz Deutschmann
Univ. Prof. Dr Leopold Neuhold
Univ. Prof. Dr Peter J. Schick
Univ. Prof. Dr Michael Speicher
Ass.-Prof. Dr jur. Armin-Bernhard Stolz
Univ. Prof. Dr Kurt Weber
Univ. Prof. Dr Andreas Zimmer

Associated medical specialists

Prof. PD DMBalic
PD DDr Susanne Bengesser
Assoc. Prof. PD Dr Jörg Lindenmann
Ao. Univ. Prof. Dr med. univ. Rainer Lipp
Univ. Prof. Dr Wolfgang Schwinger
Assoc. Prof. PD Dr Georg Singer
Univ. Prof. Dr Peter Wolf

Medizinische Universität Graz, Auenbruggerplatz 2, A-8036 Graz, www.medunigraz.at
Legal form: Legal person under public law in accordance with the Universities Act 2002 Information: University bulletin VAT ID no: ATU 575 111 79 Bank details: Raiffeisen-
Landesbank Steiermark IBAN: AT443800000000049510, BIC: RZSTAT2G

T.C.
İSTANBUL ÜNİVERSİTESİ
İSTANBUL TIP FAKÜLTESİ
KLİNİK ARAŞTIRMALAR ETİK KURULU

Sayı : 2011-KAEK-57 – 722 **Tarih : 21.04.2022**

Konu : Klinik Araştırma (SPIOMET4HEALTH) hk.

Monitör Medikal Araştırma ve Danışmanlık Ltd. Şti.
Cumhuriyet Mah. Hacıahmet Silahşör Cad, Yeniyol Sk. Now Bomonti No:2, Kat:7
Ofis No:58 P.K.34440 Şişli/İstanbul
Tel: (212) 234 12 60 Fax: (212) 234 19 53

İlgi : 21.03.2022 tarihli yazınız

Koordinatörlüğünü ve sorumlu araştırıcılığını İstanbul Üniversitesi İstanbul Tıp Fakültesi Çocuk Sağlığı ve Hastalıkları Anabilim Dalı Öğretim Üyesi Prof. Dr. Fatma Feyza DARENDELİLER' in üstlendiği 2022/137 dosya numaralı "Polikistik over sendromu (PCOS) olan ergen ve genç yetişkin kadınlarda (AYA) sabit doz Spironolakton, Pioglitazon ve Metformin (SPIOMET) kombinasyonu etkinliği, toleransı ve güvenliliğini değerlendirmek için Faz II, randomize, çok merkezli, çok uluslu klinik çalışma" başlıklı araştırmasına ait; Klinik Araştırmalarda Önemli Değişiklik Başvuru Formu, Bilgilendirilmiş Gönüllü Olur Formu (Ergen, Ebeveyn ve Yetişkin) (10.03.2022 tarihli, Versiyon 1.0, Türkçe), Sigorta Zeyilnamesi (Poliçe No: TRLSCA40621, Zeyil No: 1, Başlangıç Tarihi: 01.04.2022 - Bitiş Tarihi: 01.04.2027, Tanzim Tarihi: 01.04.2022), Sigorta Sertifikası (Poliçe No: TRLSCA40621, Başlangıç Tarihi: 01.04.2022 - Bitiş Tarihi: 01.04.2027, Tanzim Tarihi: 14.03.2022), Sigorta Sertifikası (Poliçe No: TRLSCA40621, Başlangıç Tarihi: 01.04.2022 - Bitiş Tarihi: 01.04.2025, Tanzim Tarihi: 14.01.2022), Sigorta Poliçesi (Poliçe No: TRLSCA40621, Başlangıç Tarihi: 01.04.2022 - Bitiş Tarihi: 01.04.2025, Tanzim Tarihi: 01.04.2022), kurulumuzun 08/04/2022 tarihli 07 sayılı toplantısında değerlendirilmiş ve uygun bulunmuştur.

Yazımızın bir örneğinin çalışma koordinatörüne ve/veya sorumlu araştırıcıya, destekleyiciye ve diğer merkezlere iletilmesi hususunda bilginizi ve gereğini rica ederim.

Prof. Dr. A.Yağız ÜRESİN

İstanbul Tıp Fakültesi Klinik Araştırmalar

Etik Kurul Başkanı

Eki: "Covid-19 pandemi tedbirleri doğrultusunda online toplantı yapılmış olup, toplantıya 11 üye katılımı ve oy birliği ile karar verilmiştir"

CERTIFICATION OF TRANSLATION

Quote number: 2205-00396

Source document: SPIOMET4HEALTH_TUR_EC approval_21Apr2022.pdf

Full Title of Documents: SPIOMET4HEALTH_TUR_EC approval_21Apr2022_EN_translated 13May2022.docx

Footer: SPIOMET4HEALTH_TUR_EC approval_21Apr2022, translated into English on 13May2022

Original Source Language of Document: Turkish
Language Document is to be Translated into: English

Declaration of Translator:

I, Jan Hirš on behalf of Skrivanek s. r. o., hereby certify that the translation into English agrees with the text of the original document in Turkish language, that there were no changes/corrections made to the original document neither the translation, and that it was translated by a certified translator fluent in both above mentioned languages.

I, Jan Hirš on behalf of Skrivanek s. r. o., also confirm that the above mentioned service has been rendered and that the translated document is to the best of my knowledge a true and faithful translation of the original document as provided by the client

With Best regards,

REPUBLIC OF TURKEY
ISTANBUL UNIVERSITY
ISTANBUL FACULTY OF MEDICINE
CLINICAL RESEARCH ETHICS COMMITTEE

Issue : 2011-KAEK-57 - 722 **Date : 21.04.2022**

Subject : About the Clinical Trial (SPIOMET4HEALTH)

Monitör Medikal Araştırma ve Danışmanlık Ltd. Şti.
Cumhuriyet Mah. Hacıahmet Silahşör Cad, Yeniyol Sk. Now Bomonti No:2, Kat:7
Ofis No:58 P.K.34440 Şişli/Istanbul
Phone: (212) 234 12 60 Fax: (212) 234 19 53

Re.: Your letter dated 21.03.2022

Application Form for Significant Changes in Clinical Trials, and the Informed Consent Form (Adolescent, Parent and Adult) (dated 10.03.2022, Version 1.0, Turkish), Insurance Addendum (Policy No: TRLSCA40621, Addendum No: 1, Start Date: 01.04.2022 - End Date: 01.04.2027, Issue Date: 01.04.2022), Insurance Certificate (Policy No: TRLSCA40621, Start Date: 01.04.2022 - End Date: 01.04.2027, Issue Date: 14.03.2022), Insurance Certificate (Policy No: TRLSCA40621, Start Date: 01.04.2022 - End Date: 01.04.2025, Issue Date: 14.01.2022), Insurance Policy (Policy No: TRLSCA40621, Start Date: 01.04.2022 - End Date: 01.04.2025, Issue Date: 01.04.2022) for the trial "A Phase II, randomised, multi–centric, multi-national clinical trial to evaluate the efficacy, tolerability and safety of a fixed dose combination of Spironolactone/Pioglitazone/Metformin (SPIOMET) for adolescent girls and young adult women (AYAs) with polycystic ovary syndrome (PCOS)" with file number 2022/137, whose coordinator and principal investigator is Prof. Dr. Fatma Feyza DARENDELİLER, Faculty Member of Istanbul University, Istanbul Faculty of Medicine, Department of Child Health and Diseases, was assessed during meeting 07 of our Committee, held on 08.04.2022 and found compliant.

I kindly request that a copy of our letter be sent to the coordinator and/or the principal investigator, the sponsor and other sites.

Prof. Dr. A.Yağız ÜRESİN

Istanbul Faculty of Medicine Clinical

Research Ethics

Committee Chairman

Attachment "In accordance with the Covid 19 pandemic measures, an online meeting was held and the meeting was attended by 11 members who made an unanimous decision."
